# Nanodroplet Array Platform for Integrated Synthesis and Screening of MEK Inhibitors: a Miniaturized Approach to Early Drug Discovery

**DOI:** 10.1002/anie.202507586

**Published:** 2025-08-18

**Authors:** Maximilian Seifermann, Julius Höpfner, Liana Bauer, Divya Varadharajan, Stefan Schmidt, Björn Fröhlich, Benjamin Wellenhofer, Charlotte Luchena, Carsten Hopf, Anna A. Popova, Pavel A. Levkin

**Affiliations:** ^1^ Institute of Biological and Chemical Systems‐Functional Molecular Systems (IBCS‐FMS), Karlsruhe Institute of Technology (KIT) Hermann‐von Helmholtz‐Platz 1 Eggenstein‐Leopoldshafen 76344 Germany; ^2^ Scivalon, Bruckenäcker 9 Stuttgart 70565 Germany; ^3^ Center for Mass Spectrometry and Optical Spectroscopy (CeMOS) Technische Hochschule Mannheim Paul‐Wittsack‐Straße 10 Mannheim 68163 Germany; ^4^ Medical Faculty Heidelberg University Im Neuenheimer Feld 280 Heidelberg 69117 Germany; ^5^ Mannheim Center for Translational Neuroscience (MCTN), Medical Faculty Mannheim Heidelberg University Theodor Kutzer‐Ufer 1–3 Mannheim 68167 Germany; ^6^ Institute of Organic Chemistry Karlsruhe Institute of Technology Fritz‐Haber‐Weg 6 Karlsruhe 76131 Germany

**Keywords:** high‐throughput, MALDI‐MSI, miniaturized plattform, MEK inhibition, solid‐phase synthesis, screening

## Abstract

Early‐stage drug discovery relies on high‐throughput screenings, which are costly and time‐intensive, limiting access for academic laboratories and small companies. A key bottleneck is the lack of miniaturization and the separation of compound synthesis from screening. We present a nanoliter droplet array platform integrating synthesis, characterization, and cell‐based screening of 325 MEK (mitogen‐activated protein kinase kinase) inhibitors, targeting the MAPK/ERK (mitogen‐activated protein kinase/extracellular signal‐regulated kinase) pathway, implicated in colorectal and pancreatic cancer. The platform enables on‐chip synthesis, MALDI‐MSI (matrix‐assisted laser desorption/ionization‐mass spectrometry imaging) characterization, and cell‐based screening within 200 nL droplets containing 20 nmol starting material (∼4 ng final compound), and only 300 cells per droplet. Screening identified 46 compounds with higher cytotoxicity than mirdametinib, a clinically approved MEK inhibitor. Molecular docking revealed a shared allosteric binding mechanism, indicating non‐competitive ATP inhibition. Synthesis and screening of all 325 compounds were completed within 7 days, requiring <10 mg of reactants, <250 µL solvent, and ∼100 µL of cell suspension (∼100,000 cells in total). Our results demonstrate that integrating miniaturized combinatorial synthesis and biological screening in a single platform can accelerate early‐stage drug discovery while reducing cost and resource use.

## Graphical abstract

We present a nanodroplet array platform that integrates solid‐phase synthesis, MALDI‐MS analysis, and cell‐based screening. Using nanoliter droplets, we synthesized and tested 325 potential MEK inhibitors and identified 46 active compounds. This miniaturized approach combines chemistry and biology on a single chip, reducing time, cost, and material use in early‐stage drug discovery.

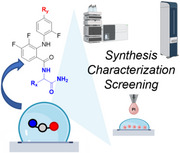

## Introduction

The development of new drugs is a complex, time‐intensive, and a costly process, typically requiring 5–20 years and $1–2 billion in investment.^[^ ^1^ ^]^ Early drug discovery alone can cost up to $1 billion and is hampered by high failure rates, which significantly inflate costs.^[^ ^2^ ^]^ This process, dominated by high‐throughput screening (HTS), is accessible primarily to large pharmaceutical companies due to the substantial expense of compound libraries and infrastructure. Traditionally, compound libraries are synthesized one at a time, stored as frozen stock solutions, and transferred into microtiter plates for biological screening. This workflow results in high costs, considerable waste, and inefficiencies due to the mismatch between chemical synthesis conducted at milliliter scales and biological assays performed at microliter scales. Current HTS methods also face challenges in liquid handling, parallelization, and miniaturization, limiting throughput and increasing environmental impact.^[^ ^3^ ^]^ Addressing these limitations is essential for reducing costs, accelerating drug discovery, and democratizing access for smaller research labs and biotech companies. Additionally, improvements in HTS could benefit related fields such as antibiotics discovery and functional materials development.

**Figure 1:**
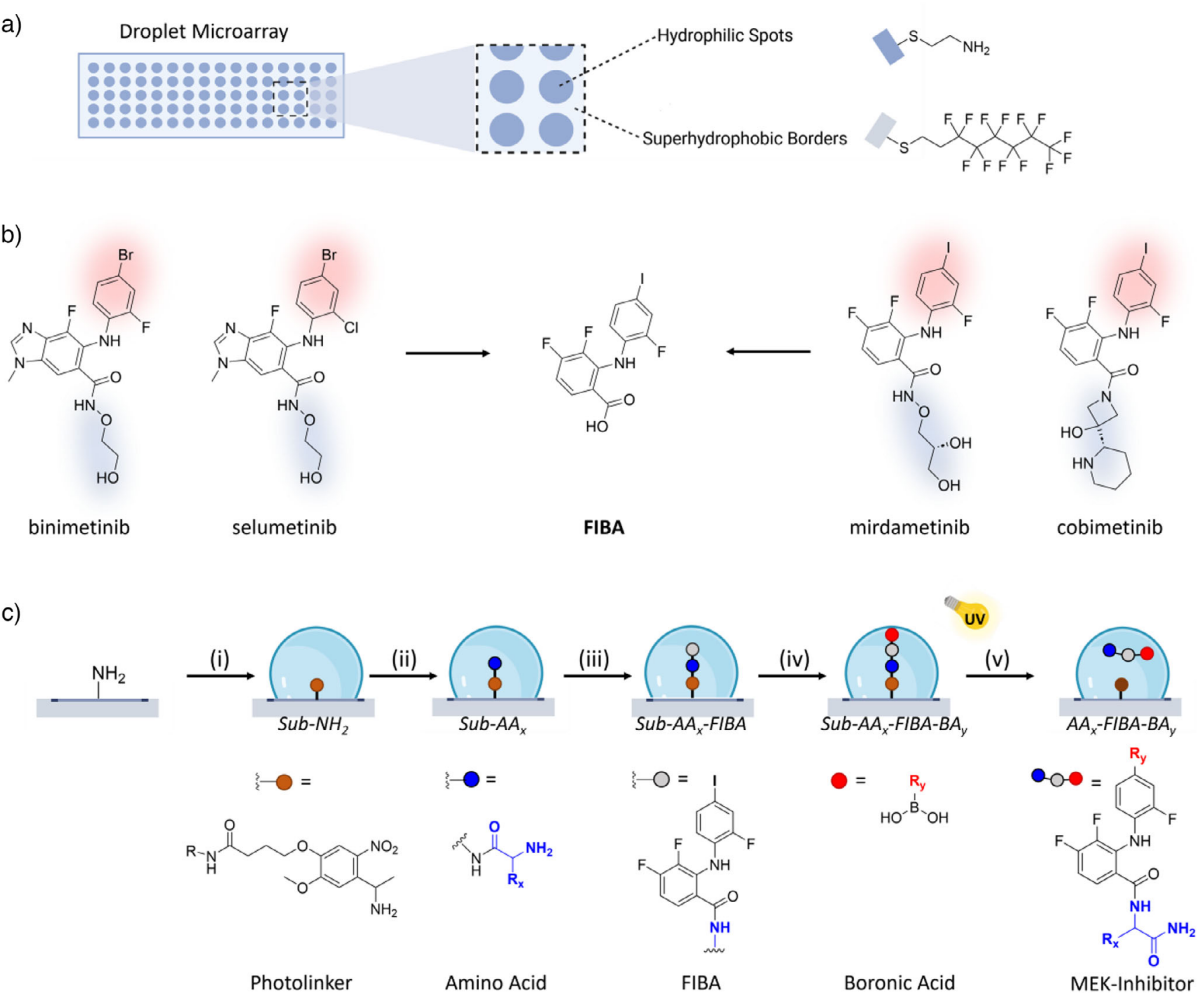
Miniaturized synthesis of MEK‐inhibitors using Nanodroplet Arrays. a) Schematic depiction of the droplet microarray presenting a pattern of hydrophilic spots on a superhydrophobic background. b) Chemical structures of the MEK‐inhibitors binimetinib, selumetinib, mirdametinib and cobimetinib, where the carbonyl side chain and the halogenated aromatic fragments are highlighted in blue and red, respectively, and the non‐highlighted part resembling the core molecule FIBA that was used for the library synthesis. c) Overview of the workflow to synthesize the MEK inhibitors on the surface of the nanodroplet array: i) attachment of the Fmoc‐photolinker (FPL) to the amino‐modified hydrophilic spot with subsequent deprotection of the amine protective group, ii) coupling of the first building block, amino acid AA_x_, onto the photolinker, iii) coupling of the core molecule FIBA onto AA_x_, iv) Suzuki coupling of the second building block, boronic acid BA_y_, onto the iodine moiety of FIBA, v) photocleavage of the final MEK‐inhibitor compound from the substrate by UV irradiation.

Efforts to miniaturize and integrate chemical synthesis with biological screening have led to development of technologies such as microtiter plate‐based synthesis, DNA‐, oligosaccharide‐, and peptide‐microarrays (SPOT synthesis), virtual library screenings, droplet‐based microfluidics, fragment based screenings, microarray technologies, and AI models for drug discovery.^[^ ^4^, ^5^, ^6^, ^7^, ^8^, ^9^, ^10^, ^11^, ^12^ ^]^ These innovations have improved automation and expanded the chemical space but fall short of achieving a fully integrated high‐throughput platform for early drug discovery. To address this, direct‐to‐biology (D2B) screening approaches are gaining traction enabling chemical synthesis and biological evaluation on the same platform to streamline workflows and reduce resource consumption.^[^ ^13^, ^14^, ^15^, ^16^ ^]^ In this context, droplet microarray (DMA) technology represents a transformative platform for high‐throughput, miniaturized drug discovery, wherein an array of hydrophilic spots on a superhydrophobic surface enables precise manipulation of thousands of nanoliter droplets in an open, wall‐less configuration (Figure 1a).^[^ ^17^, ^18^, ^19^ ^]^ Previously, we demonstrated the capability of this technology for synthesizing PROTAC‐like molecules and testing their activity on HT‐29 colon cancer cells.^[^ ^20^ ^]^ We have also shown the synthesis of a lipid library and on‐DMA testing with cells for searching new lipid‐based cell transfection reagents.^[^ ^21^ ^]^ Developing this technology, herein we use *Nanodroplet Array*s to synthesize and pre‐screen a large library and select potential drug compounds against the MAPK/ERK (mitogen‐activated protein kinase/extracellular signal‐regulated kinase) pathway which is dysregulated in one‐third of all human cancers.^[^ ^22^ ^]^ This pathway, involving key proteins such as ERK, rat sarcoma GTPase (Ras), rapidly accelerated fibrosarcoma kinase (Raf), and mitogen‐activated protein kinase kinase (MEK) contributes to cell proliferation, chemoresistance, and other tumor‐related phenotypes.^[^ ^23^ ^]^ MEK, a downstream kinase in this pathway, is particularly attractive for therapeutic intervention due to its role in mediating various dysregulations.^[^ ^24^, ^25^ ^]^ Despite its promise, only five FDA‐approved MEK inhibitors are available—trametinib, cobimetinib, selumetinib, and binimetinib, and, most recently approved in February 2025 for the treatment of neurofibromatosis type 1 (NF‐1), mirdametinib (Figure 1b).^[^ ^26^, ^27^ ^]^ The morbidity of half of the NF‐1 patients is impacted due to the formation of plexiform neurofibromas (PNs), a benign nerve tumor which is conventionally eliminated by surgery.^[^ ^28^ ^]^ Only selumetinib and mirdametinib are currently approved by FDA to treat inoperable NF‐1 associated PNs, with mirdametinib being the only one to treat both adults and children.^[^ ^29^ ^]^ While several other drugs are under clinical trial, most MEK inhibitors are not efficient enough as single‐drug treatment and are hindered by acquired resistance mechanisms.^[^ ^30^ ^]^ This highlights the need for more potent and selective MEK inhibitors and consequently, an accessible high‐throughput chemical and biological screening method. Addressing this combined need, we synthesized and analyzed 325 potential MEK‐inhibitor compounds while subsequently testing their activity against HT‐29 cells on one platform in a modular approach using Nanodroplet Arrays. Three consecutive steps were performed to achieve this – **Step 1**: Preparation of Nanodroplet Array surface and synthesis of a library of 325 MEK‐inhibitor compounds on the surface (schematic overview as shown in Figure 1c), **Step 2**: Further miniaturization and on‐chip analysis using MALDI‐MSI, and **Step 3**: On‐chip screening of the final library for cytotoxicity using HT‐29 cells. The library of MEK‐inhibitors were prepared on the Nanodroplet Array surface by chemically modifying a core molecule “3,4‐difluoro‐2‐(2‐fluoro‐4‐iodophenylamino)benzoicacid” (**FIBA**) that was attached to the surface via a photocleavable linker **“**4‐{4‐[1‐(9‐Fluorenylmethyloxycarbonylamino)ethyl]‐2‐methoxy‐5‐nitrophenoxy}butanic acid” (Fmoc‐photolinker, FPL). This approach for library synthesis is modular allowing for unlimited drug compound synthesis as the linker can be modified with various amino acids containing carboxylic acids via amidation and the **FIBA** core molecule by Suzuki coupling on the iodo‐moiety. To prove the successful on‐surface synthesis, the drug compounds were analyzed using MALDI‐MSI showing an >85% success rate in the on‐chip synthesis method. As further biological testing was also done on the substrate, it shows the scalability of the Nanodroplet Array to screen and pre‐select MEK‐inhibitors that are crucial for early‐stage drug discovery.

This approach is not only useful for one type of drug compound screening but can easily be extended toward the synthesis of other drug compounds leading to significant advancement in early‐stage drug discovery. This high‐throughput Nanodroplet Array minimizes reagent use, reduces waste, and enhances efficiency compared to traditional HTS workflows. Ultimately, it not only accelerates the identification of promising compounds but can also potentially democratize access to high‐throughput drug discovery for academic and smaller industrial labs.

## Results and Discussion

Comparing the chemical structures of the currently approved MEK‐inhibitors, two significant similarities amongst them can be identified: a halogenated aniline fragment and a substituted carbonyl. While the carbonyl group is mostly needed for binding the Lys97 residue inside MEK, the aromatic fragment binds to the hydrophobic pocket containing Leu115, Leu118, Val127, and Met143.^[^ ^31^ ^]^ As the structural orientation of the drug molecules affects the binding affinity at the active sites, altering the structure of the drug molecule itself can also affect these interactions in turn. Capitalizing on this, for the experimental design we derivatized mirdametinib, approved for the treatment of neurofibromatosis type I, and cobimetinib, approved as treatment against RAF‐mutant melanomas as well as histiocytic neoplasms, as lead compounds.^[^ ^32^, ^33^, ^34^ ^]^ We chose **FIBA** as the core molecule for further modification as it closely resembles the core structure of mirdametinib and cobimetinib (Figure 1b). Using a linear synthetic reaction cascade, we modified both the carbonyl and iodo aniline moiety directly on the Nanodroplet Array as a solid support. The substrate was first prepared as described previously on standard glass slides coated with a nanoporous poly(2‐hydroxyethyl methacrylate‐*co*‐ethylene dimethacrylate) p(HEMA‐*co*‐EDMA) polymer.^[^ ^18^, ^19^ ^]^ The p(HEMA‐co‐EDMA) surface was esterified with 4‐pentynoic acid followed by the UV‐induced thiol‐yne click reaction sequentially with 1H,1H’,2H,2H’‐perfluorodecanethiol and cysteamine hydrochloride to form hydrophilic (HL) round spots of controlled size (such as 900 µm diameter) surrounded by superhydrophobic (SH) borders, respectively. The amino‐functionalized spots (Figure 1a, blue HL spots) provided a solid‐phase functionality for further synthesis of MEK‐inhibitors. Fmoc‐protected photolabile linker FPL that can easily be cleaved off using UV irradiation was covalently attached to the amines on the HL spots as an anchor point to the Nanodroplet Array surface [*Photolinker‐modified substrate* (*Sub*) shown in Figure 1c]. After the attachment and deprotection of the photolabile linker [*Sub‐NH_2_* shown in Figure 1c, (i)] 13 different naturally occurring amino acids (AAs) were attached in a subsequent step leading to the first intermediates [**Intermediate 1** = *Sub*‐*AA_x_* shown in Figure 1c, (ii)]. As various amino acids can be attached to the same linker, we used this modular approach to obtain an intermediate library of MEK‐inhibitor compounds. The core structure **FIBA** was then attached to *Sub*‐*AA_x_* surface (formation of *Sub‐AA_x_‐FIBA*) followed by attachment of 25 structurally diverse boronic acids (BAs) leading to the formation of *Sub*‐*AA_x_*‐*FIBA*‐*BA_y_* [Figure 1c, (iv)] whereby “*x*” and “*y*” are the modular amino acid and boronic acid entities (see Table 1 for list of amino acids and boronic acids used). The combination of the AA_x_ and BA_y_ moieties with **FIBA** in the MEK‐inhibitor compounds **1 – 325** are represented in the Supporting Information (SI), Table S1. The final potential drug compounds were obtained by amidation and Suzuki coupling on the free amino and iodine groups decorated on the Nanodroplet Array surface, respectively [Figure 1c, (v)]. To prove the formation of a library of MEK‐inhibitor molecules, they were directly analyzed on‐chip in Step 2 using MALDI‐MSI.

**Table 1:** Overview of the 13 amino acids (AA_x_) and 25 boronic acids (BA_y_) used as building blocks to synthesize the library of 325 MEK‐inhibitor compounds AA_X_‐FIBA‐BA_y_.

Amino acid (AA_x_)			Boronic acid (BA_y_)						MEK‐inhibitor compound	
Name	X	Structure	Name	Y	Structure	Name	Y	Structure	#	AA_x_‐FIBA‐BA_y_
**L‐Val**	**1**	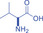	**(2‒fluoro‒4‐methylphenyl) boronic acid**	**1**	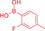	**p‒tolyl boronic acid**	**14**	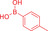	**1–25**	**AA_1_‐FIBA‐BA_1‐25_**
**L‐Ala**	**2**	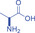	**(4‒methoxy‒2‐methylphenyl) boronic acid**	**2**	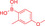	**(3‒(trifluoro methyl)phenyl) boronic acid**	**15**	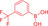	**26–50**	**AA_2_‐FIBA‐BA_1‐25_**
**ß‐Ala**	**3**	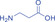	**(4‒carbamoyl phenyl)boronic acid**	**3**	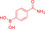	**(3,4,5‒trifluoro phenyl)boronic acid**	**16**	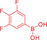	**51–75**	**AA_3_‐FIBA‐BA_1‐25_**
**L‐Arg (Pmc)**	**4**	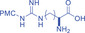	**(3‒isopropyl phenyl)boronic acid**	**4**	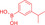	**(4‒ (2‒methoxy ethoxy)phenyl) boronic acid**	**17**	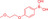	**76–100**	**AA_4_‐FIBA‐BA_1‐25_**
**L‐Asn**	**5**	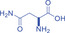	**(3,5‐dimethoxy phenyl)boronic acid**	**5**	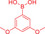	**Phenylboronic acid**	**18**	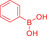	**101–125**	**AA_5_‐FIBA‐BA_1‐25_**
**L‐Gln**	**6**	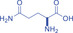	**(3,4‒difluoro phenyl)boronic acid**	**6**	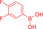	**(2‒butoxy‒5‐methylphenyl) boronic acid**	**19**	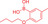	**126–150**	**AA_6_‐FIBA‐BA_1‐25_**
**L‐Glu (OtBu)**	**7**	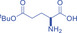	**(4‐propylphenyl) boronic acid**	**7**	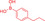	**(2,4‒dimethoxy phenyl)boronic acid**	**20**	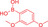	**151–175**	**AA_7_‐FIBA‐BA_1‐25_**
**L‐His(Trt)**	**8**	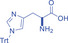	**(3‒fluoro‒4‐methoxyphenyl) boronic acid**	**8**	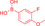	**benzo[d][1,3] dioxol‐5yl boronic acid**	**21**	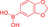	**177–200**	**AA_8_‐FIBA‐BA_1‐25_**
**Phe‐Phe**	**9**	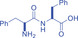	**(3‒chloro‒5‐fluorophenyl) boronic acid**	**9**	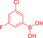	**(4‒ (trifluoro methyl)phenyl) boronic acid**	**22**	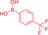	**201–225**	**AA_9_‐FIBA‐BA_1‐25_**
**L‐Leu**	**10**	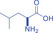	**(3‒hydroxy phenyl)boronic acid**	**10**	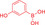	**(4‒hydroxy phenyl)boronic acid**	**23**	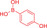	**226–250**	**AA_10_‐FIBA‐BA_1‐25_**
**L‐Phe**	**11**	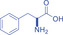	**(3‐ethoxyphenyl)boronic acid**	**11**	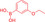	**(4‒hydroxy phenyl)boronic acid**	**24**	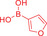	**251–275**	**AA_11_‐FIBA‐BA_1‐25_**
**L‐Ile**	**12**	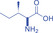	**(4‒isopropoxy phenyl)boronic acid**	**12**	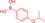	**(6‒hydroxy naphthalen‐2‐yl) boronic acid**	**25**	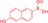	**276–300**	**AA_12_‐FIBA‐BA_1‐25_**
**L‐Ser**	**13**	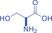	**(4‐chlorophenyl) boronic acid**	**13**	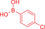				**301–325**	**AA_13_‐FIBA‐BA_1‐25_**

**Figure 2:**
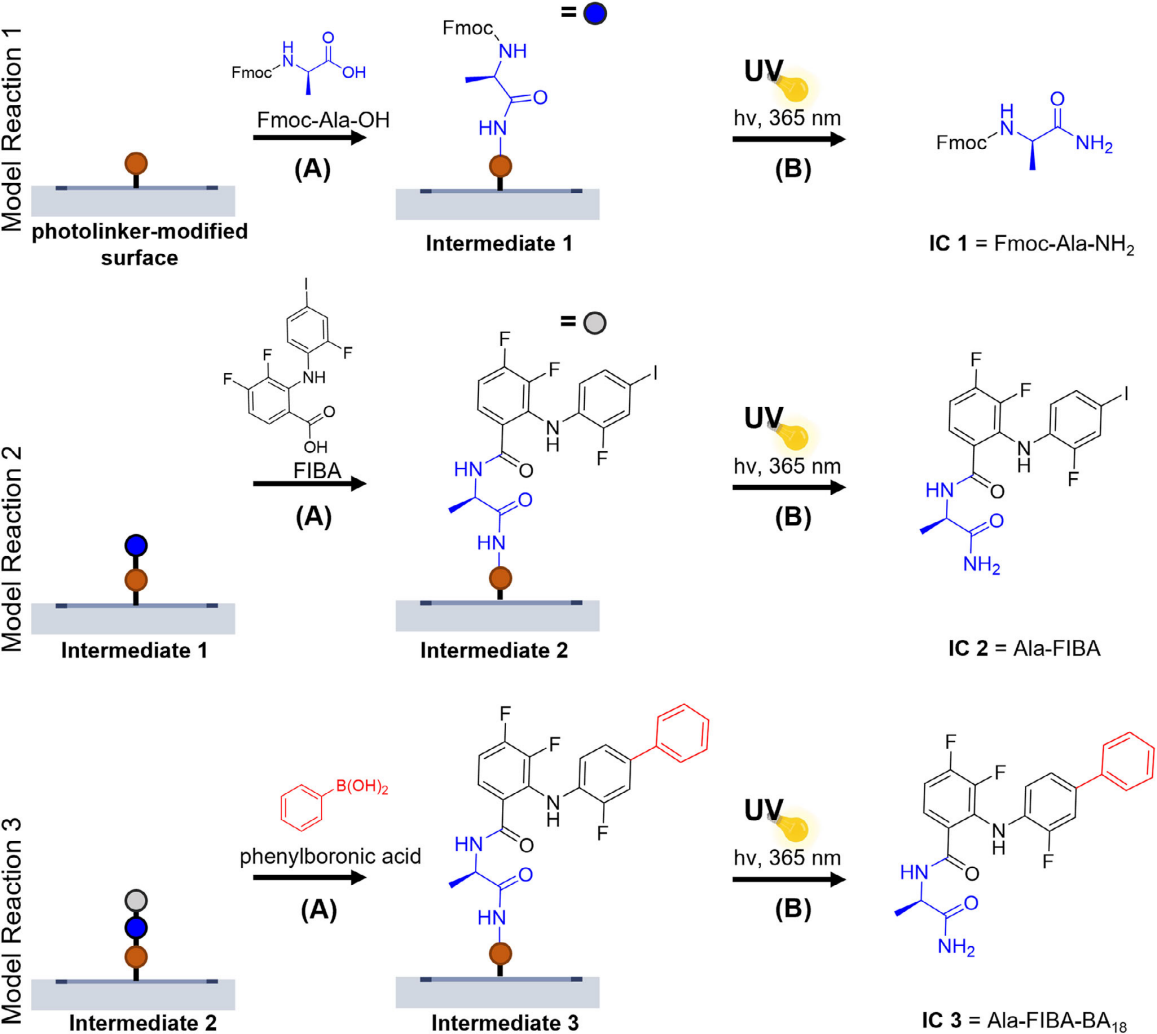
Model reactions 1–3 on the substrate to optimize reaction conditions for the library synthesis. Model reaction 1: a) Fmoc‐Ala‐OH (Fmoc‐AA_2_) was coupled onto the photolinker using coupling reagents hydroxybenzotriazole (HOBt) and N,N’‐diisopropylcarbodiimide (DIC) to form the surface Intermediate 1. b) The immobilized Intermediate compound 1 (IC 1  =  Fmoc‐Ala‐NH_2_) was released from the surface by UV irradiation at 365 nm at various irradiation times. Model reaction 2: a) Amidation of the core structure FIBA to the deprotected amino acid AA_2_ [coupling conditions as in Model reaction 1 (a)] to form the surface Intermediate 2. b) Intermediate compound 2 (IC 2 = Ala‐FIBA) was released by UV irradiation for 40 min at 365 nm using the conditions as in Model Reaction 1. Model Reaction 3: a) Phenylboronic acid (BA_18_) was attached to 2‐fluoro‐4‐iodophenyl moiety of FIBA by Suzuki coupling to form the surface Intermediate 3. b) The intermediate compound 3 (IC 3  =  Ala‐FIBA‐BA_18_) was released by UV irradiation as in Model reaction 1.

To optimize the series of reactions described in Figure 1, a model droplet array surface was used with larger spot diameters and loading volumes, namely 2.8 mm diameter of 80 HL spots and sample volumes of 3–5 µL per spot. With this, sufficient amounts of intermediate and final products were synthesized, making their chemical analysis and reaction optimization feasible using conventional analytical methods. Consequently, the reaction performance was characterized using a model workflow with known reactants, where 3 intermediates **AA_2_‐NH_2_**, **AA_2_‐FIBA**, **AA_2_‐FIBA‐BA_18_** were sequentially cleaved off from *Sub‐AA_x_* (*x *= 2, and *AA_2_* = Alanine or Ala), *Sub‐Ala‐FIBA*, and *Sub‐Ala‐FIBA‐BA_y_* (y = 18, and *BA_18_* = phenylboronic acid) and analyzed. The intermediate surfaces are **Intermediates 1–3** while the intermediate compounds released from the surface are represented as **IC 1–3** in Figure 2.

**Figure 3:**
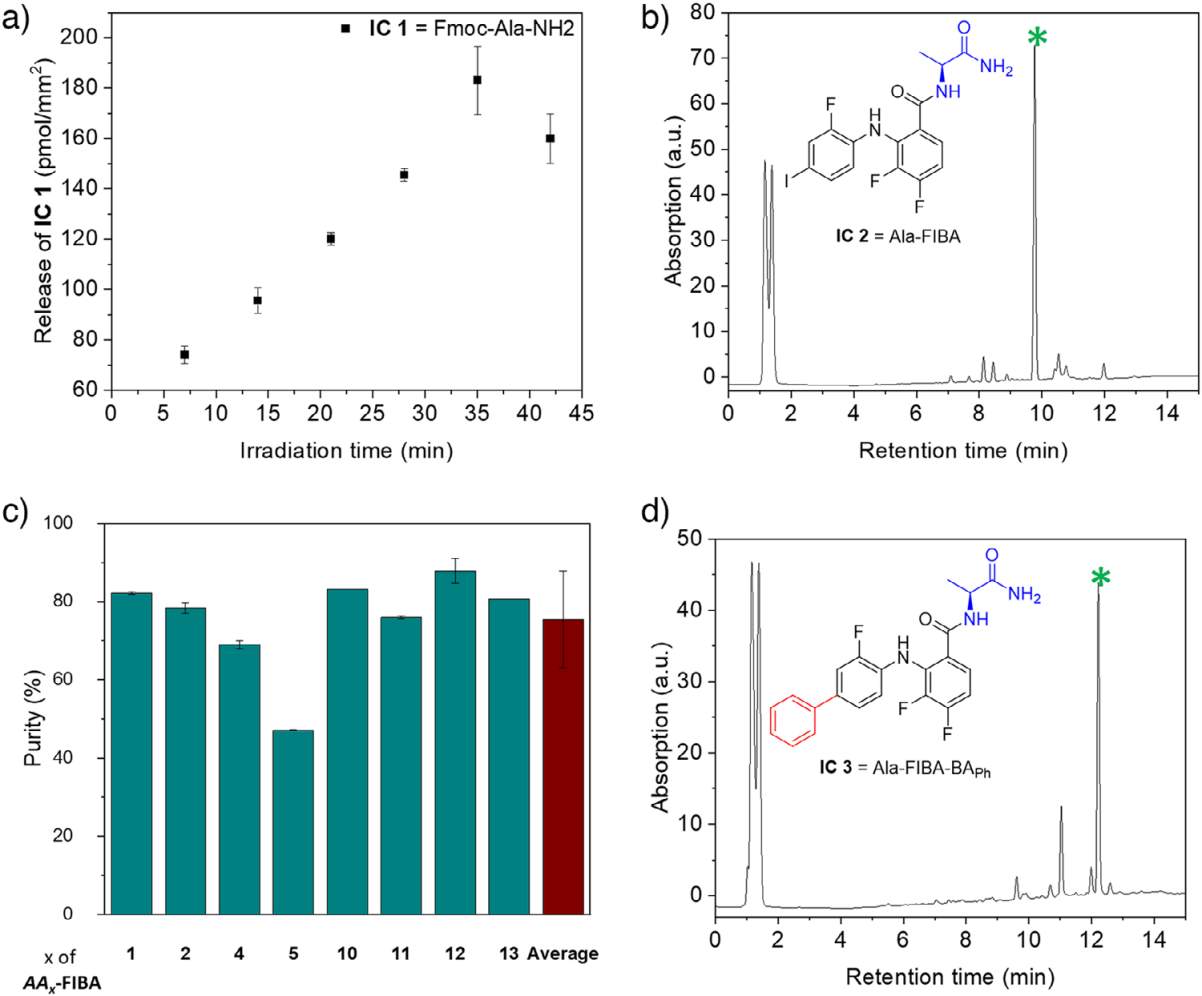
Analysis of the compounds formed in the model reactions. a) Amount of released IC 1 (Fmoc‐Ala‐NH_2_) in pmol mm^−2^ at various irradiation times from 5 to 45 min measured via LC‐MS. b) LC‐MS chromatogram of IC 2 (Ala‐FIBA). The marked peak in green corresponds to the main product peak. The integration of the signals was used for the determination of the purity of IC 2. c) The purity of eight IC 2 compounds was analyzed by LC‐MS and ranged from 44% to 84%, with an average purity of 76 ± 12%. The purity is depicted based on the amino acid (AA_x_) used. d) LC‐MS chromatogram of IC 3 (Ala‐FIBA‐BA_Ph_). The marked peak in green corresponds to the main product peak. The integration of the signals was used for the determination of purity and conversion rate of IC 3. Measurements were performed in triplicates and calculated as mean, error bars show the standard deviation.

Crucial steps in this workflow were quantification and optimization of controlled product release at every reaction step after the cleavage of every intermediate compound (**IC 1–3**) from the substrate. Firstly, to assess the model amino acid (alanine) loading (Figure 2, **Intermediate 1,** *Sub‐AA_2_*) and its release kinetics from the surface forming **IC 1**, the *Sub*‐*AA_2_* surface was washed and irradiated with UVA light (365 nm) for varying durations. We then collected the released compounds in dimethylformamide (DMF) and water to analyze the combined solutions using liquid chromatography‐mass spectrometry (LC‐MS) and quantified the amount through a calibration curve (Figures S1, S2). The release of the compounds plateaued after 30–35 min revealing a maximum Fmoc‐alanine loading capacity of 160 pmol mm^‐^^2^ (Figure 3a). This maximum loading was used for further quantification of product release after the subsequent reaction steps, regardless of the used amino acid on the *Sub‐NH_2_* surface. Secondly, **FIBA** was attached to the deprotected **Intermediate 1** surface (S*ub*‐*AA_2_*) under similar conditions leading to the formation of **IC 2** (Figure 2, Model reaction 2). To achieve a maximum release, **IC 2** was released by irradiation with UV light for 40 min as optimized in the first model reaction. The purity of **IC 2** was calculated as 78% by integration of the UV spectrum signals in the LC‐MS measurement, where the product signal integral was divided by the sum of all integral values (Figures 3b, S3, SI, Table S2). We determined the amount of **IC 2** released by calibrating the UV absorption in the LC‐MS measurements with the same compound prepared and purified in a round bottom flask (Calibration curve in SI, Figure S4). Using the calibration curve, we calculated a product release of 122 ± 16 pmol mm^−2^ in two independent experiments which translated to a conversion rate of 75 ± 10% at a loading capacity of 160 pmol mm^−2^ of alanine amide. To quantify the purity of **Intermediate 2** compounds, we selected 8 Fmoc‐protected amino acids and coupled each one of them to the core motif **FIBA** to obtain the first intermediate library (**Intermediate 2**, as shown in Figure 2). After cleavage of these intermediates from the surface leading to IC **AA_x_‐FIBA**, we calculated their purities using the aforementioned method to be 76 ± 12% on average (Figure 3c). Subsequently, **Intermediate 2** was coupled with BA_18_ using previously established conditions to obtain **IC 3**.^[^ ^35^ ^]^ The reaction was successful on the substrate at 83% conversion rate of the Suzuki coupling reaction (Figure 3d, integration and calibration in SI Figures S5, S6).

Expanding this to obtain a larger library of **Intermediate 3** MEK‐inhibitors, each of these 8 **Intermediate 2** (*Sub‐AA_x_‐FIBA*) compounds were coupled by Suzuki‐coupling with 2 boronic acids (*BA*_18_, *BA*_20_) leading to 16 **Intermediate 3** (*Sub‐AA_x_‐FIBA‐BA_18,20_*) compounds on the substrate (for respective **Intermediate 2**, see Figure 3c). After cleavage from the substrate, the yield of the **Intermediate 3** compounds was quantified by their conversion rate of the Suzuki coupling reaction. The MEK‐inhibitor compounds were the main peak in the LC‐MS chromatograms and integrating them as described previously (calculation shown in SI, Table S3) gave an overall purity of 56 ± 11%. Although the purity was not high, we assessed this to be sufficient for a primary biological screening meant only for pre‐selecting MEK‐inhibitor compounds. The median conversion rate of the final Suzuki coupling was 86 ± 10%, when the **AA_x_‐FIBA** loading is 122 pmol mm^−2^ (final amount = 104.92 pmol mm^−2^, Table S3). Therefore, the overall yield for the two reaction steps (**Intermediate 2** and **3**) to obtain **IC 3** compounds valued 65% (i.e., 65% of maximum amino acid loading described previously to be 160 pmol mm^−2^). In summary, model reactions 1–3 were used to set the following reaction conditions for the library synthesis of MEK inhibitors: i) amino acid loading at 160 pmol mm^−^^2^ for the formation of **Intermediate 1;** ii) all intermediates and the MEK‐inhibitors will be cleaved off from the substrate by irradiation with UVA light at 365 nm for 40 min to achieve maximum product concentration.

**Figure 4:**
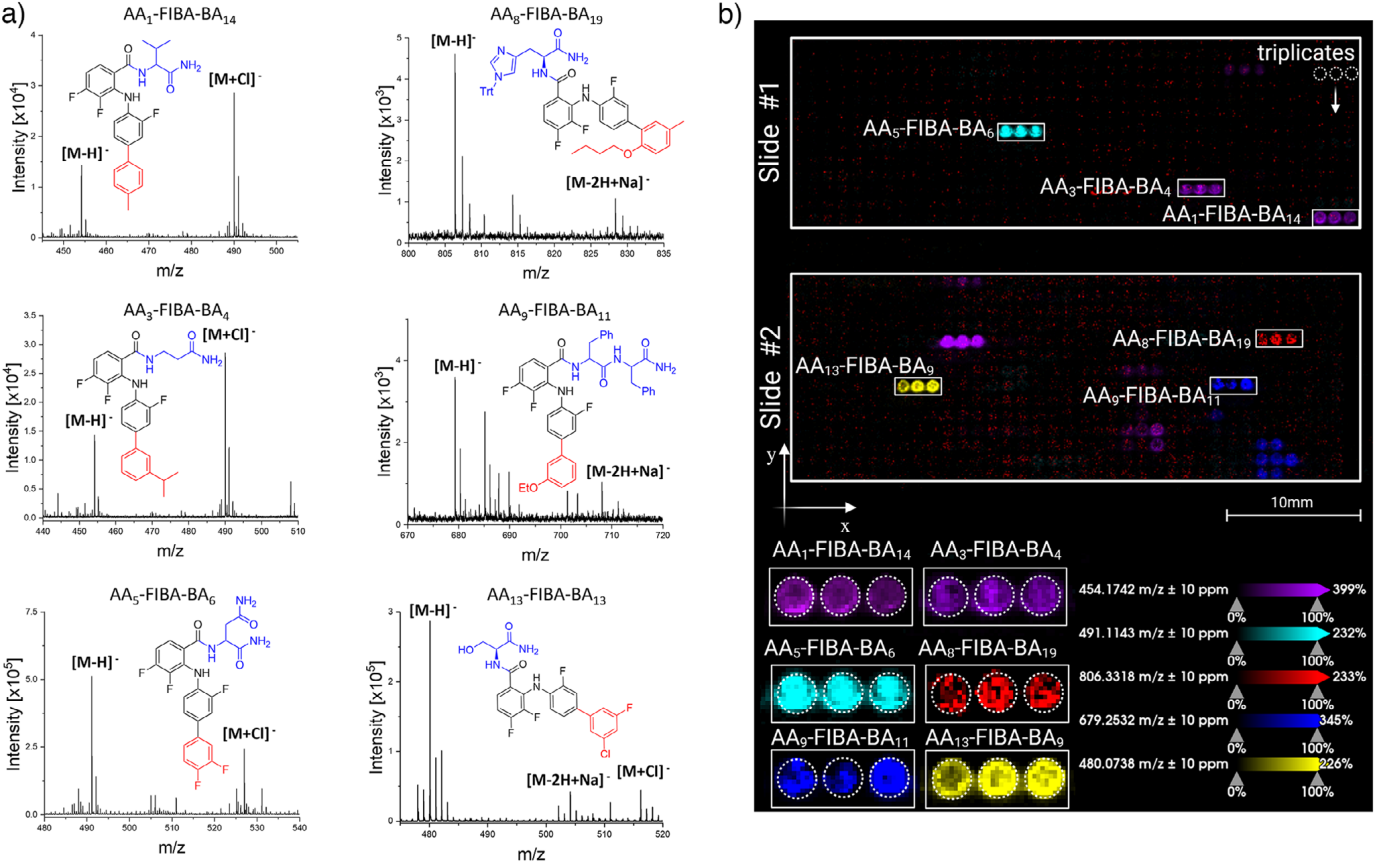
MALDI‐MSI analysis of the MEK‐inhibitors library on a standard MALDI target plate and on the ITO Nanodroplet Array substrate. a) Exemplary mass spectra obtained from compound screening after transferring the synthesized product on a ground steel target plate and subsequent analysis by MALDI MS. Respective structures and the location of the [M‐H]^−^ peak and the [M+Cl]^−^ adduct are indicated. Compounds were synthesized on 1.4 mm spots of a droplet array substrate. b) Direct MALDI‐MS imaging of two ITO‐modified Nanodroplet Array slides #1 and #2 with 900 µm spots on which solid‐phase synthesis of the MEK‐inhibitor compounds was carried out. Ion intensity of the [M‐H]^−^ ion for eight different compounds (arbitrarily chosen) are indicated by different color bars in the overview ion image. Compounds were synthesized in triplicates. The intensity scale shows the signal intensity.

The calculated conversion rate to obtain the final MEK‐inhibitor compounds would translate to a concentration of 110–250 µM on the droplet array with 320 spots of 1.4 mm diameter and an assay volume of 1 µL. However, as primary biological screenings are typically performed with drug compounds in the low‐micromolar range, we optimized the final product release from the substrate.^[^ ^36^ ^]^ The amount of released product can be controlled by either reducing the reagent concentrations, the irradiation time or the initial concentration of photolinker. Thus, to reduce the final amount, **Intermediates 2** and **3** were prepared again on a droplet array platform with 2.8 mm sized spots with varying concentrations of the FPL solution between 0.3 and 100 mM and irradiation for 40 min (SI, Figure S7). The release of **IC 2** saturated after substrate modification with 2.5 mM concentrated FPL solution, while a rapid decrease in its release was observed using lower concentrations of the photolinker. By using 0.3 mM FPL, the final loading of **IC 3** would still translate to an assay concentration of 55 µM on 1.4 mm sized droplet arrays. Since the optimal conditions for biological screening were not achieved yet, we tested a lower concentration of 0.1  and 0.3 mM FPL while varying the irradiation time. Using the lower concentration of FPL, we saw that **IC 2** release saturated in less than 6 min, while it took 18 minutes using 0.3 mM FPL (SI, Figure S8). At 6 min, 12 pmol mm^−2^ of **IC 2** was released, corresponding to a calculated concentration of approximately 10 µM of **IC 3** based on the previous conversion of the Suzuki coupling. To validate these calculated results, **IC 3** was synthesized in triplicates on *Sub‐NH_2_* using 0.1 mM FPL and analyzed by LC‐MS after cleavage. The absolute amount of **IC 3** released was calculated against a calibration curve made by serial dilution of pure **IC 3** in a round bottom flask (SI, Figure S6) to be 6.31 ± 0.14 pmol mm^−2^ on the substrate, that is, 9.71 ± 0.22 µM on droplet array spots of 1.4 mm diameter used for the biological screening. With this optimization of the chosen reaction parameters described above, the release of desired assay concentrations of **IC 3** on **Intermediate 3** for biological screening was achieved. In order to validate the feasibility of compound identification with further miniaturization where the sensitivity of LC‐MS is exceeded, a library of 320 **AA*_x_*‐FIBA‐BA*_y_*** compounds was synthesized on the Nanodroplet Array platform with 1.4 mm sized spots in two independent repetitions. The library was reduced to 320 compounds due to the limitation of the maximum number of reaction spots achievable on a single chip. Here, 300  and 400 nL reaction volumes for the formation of **Intermediate 2** (*Sub‐AA_x_*) and **Intermediate 3** (*Sub‐AA_x_‐FIBA‐BA_y_*) were used, respectively. The compounds **AA_x_‐FIBA‐BA_y_** were cleaved off, redissolved in a mixture of DMF and water (2:1, 1 µL spot^−1^) and manually transferred to a ground steel target plate for MALDI analysis (procedure as described in experimental section). We successfully identified 297 of 320 compounds (93%) as shown in Table 2 (exemplary spectra in Figure 4a). Additional MS/MS measurements of selected compounds and fragment analysis support product formation of the target structures on the surface (SI, Figures S9–S13).

**Table 2:** Summary of the total number of identified final products analyzed with MALDI‐MS on a ground steel MALDI target plate after synthesis has been carried out on spots of 1.4 mm diameter in duplicates including manual transfer after cleavage versus direct analysis with MALDI‐MSI on the ITO‐modified chips where synthesis has been carried out on spots with 900 µm diameter in triplicates.

AA_x_	Structure	Found on MALDI plate	Found on chip	BA_y_	Structure	Found on MALDI plate	Found on chip	BA_y_	Structure	Found on MALDI plate	Found on chip
**AA_1_**	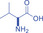	21	22	**BA**_1_	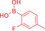	13	12	**BA**_14_	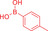	13	12
**AA_2_**	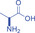	22	24	**BA**_**2**_	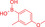	12	11	**BA**_**15**_	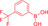	13	6
**AA_3_**	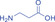	24	22	**BA**_**3**_	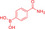	11	11	**BA**_**16**_	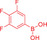	13	13
**AA_4_**	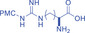	14	18	**BA**_**4**_	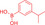	13	12	**BA**_**17**_	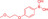	10	13
**AA_5_**	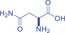	23	24	**BA**_**5**_	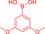	12	12	**BA**_**18**_	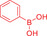	7	13
**AA_6_**	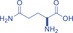	25	24	**BA**_**6**_	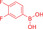	12	12	**BA**_**19**_	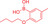	13	13
**AA_7_**	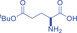	25	24	**BA**_**7**_	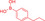	13	13	**BA**_**20**_	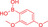	13	12
**AA_8_**	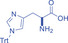	25	18	**BA**_**8**_	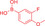	13	13	**BA**_**21**_	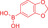	13	13
**AA_9_**	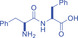	25	22	**BA**_**9**_	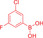	13	13	**BA**_**22**_	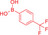	13	13
**AA_10_**	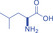	25	20	**BA**_**10**_	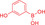	12	13	**BA**_**23**_	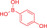	11	12
**AA_11_**	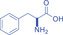	25	22	**BA**_**11**_	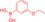	13	13	**BA**_**24**_	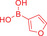	8	12
**AA_12_**	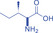	24	24	**BA**_**12**_	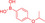	13	10	**BA**_**25**_	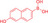	10	1
**AA_13_**	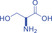	24	25	**BA**_**13**_	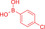	12	12	Total rate		297 (93%)	285 (89%)

After successful identification, the goal was to achieve Step 2, the qualitative analysis of MEK‐inhibitor formation directly on the same substrate where they were synthesized to further facilitate the high‐throughput analysis. For this, ITO‐modified (Indium Tin Oxide) Nanodroplet Array substrates with 1152 spots of 900 µm diameter to synthesize the total library of 325 MEK‐inhibitor compounds in triplicates using only 100 nM and 200 nL reaction volume per spot for the intermediate steps. After the compounds were cleaved off, the matrix was dispensed directly on the substrate and used for MALDI‐MSI on a timsTOF quadrupole‐time‐of‐flight mass spectrometer. For the first time, we proved the successful integration of synthesis and analysis on the same substrate sequentially by directly comparing the MALDI spectra of the final compounds from the integrated platform to that obtained by manual transfer onto ground steel plates. We clearly identified 285 (89%) of 325 compounds using the integrated approach (Table 2). Additionally, the confinement of the compounds to their respective reaction spots could be visualized in both glass substrates together containing the 325 compounds in triplicates (Figure 4b). The intensity of the primary product ions of arbitrarily selected eight compounds are highlighted in Figure 4b (Slide #1 and Slide #2). This proves that the formation and identification of the final compound was successful even on 900 µm sized spots and could be contained within the borders of each reaction spot. While the total rate of identified compounds is quite similar using both approaches, we observed a low identification rate of specific compounds containing the building block **BA_15_** and **BA_25_**_._ In comparison to the conventional measurement on the ground steel plate, the sensitivity of the measurement was reduced on the ITO‐modified substrate. Nonetheless, with a total rate of 89%, this approach proved the ability to integrate the synthesis and analysis using MALDI‐MSI directly on one substrate, showcasing the potential of the all‐in‐one platform (overview of which compounds were identified successfully in SI, Figure S14). The synthesis and characterization of a large library of MEK‐inhibitors on the surface (**Intermediate 3**, *Sub‐AA_x_‐FIBA‐BA_y_*) was therefore successfully finalized and thus the library was prepared for biological screening against HT‐29 cells (Step 3).

The total number is shown in dependence of the used building block where each AA is used in 25 and each BA in 13 different compounds. The total rate refers to the fraction of identified compounds from the complete library.

**Figure 5:**
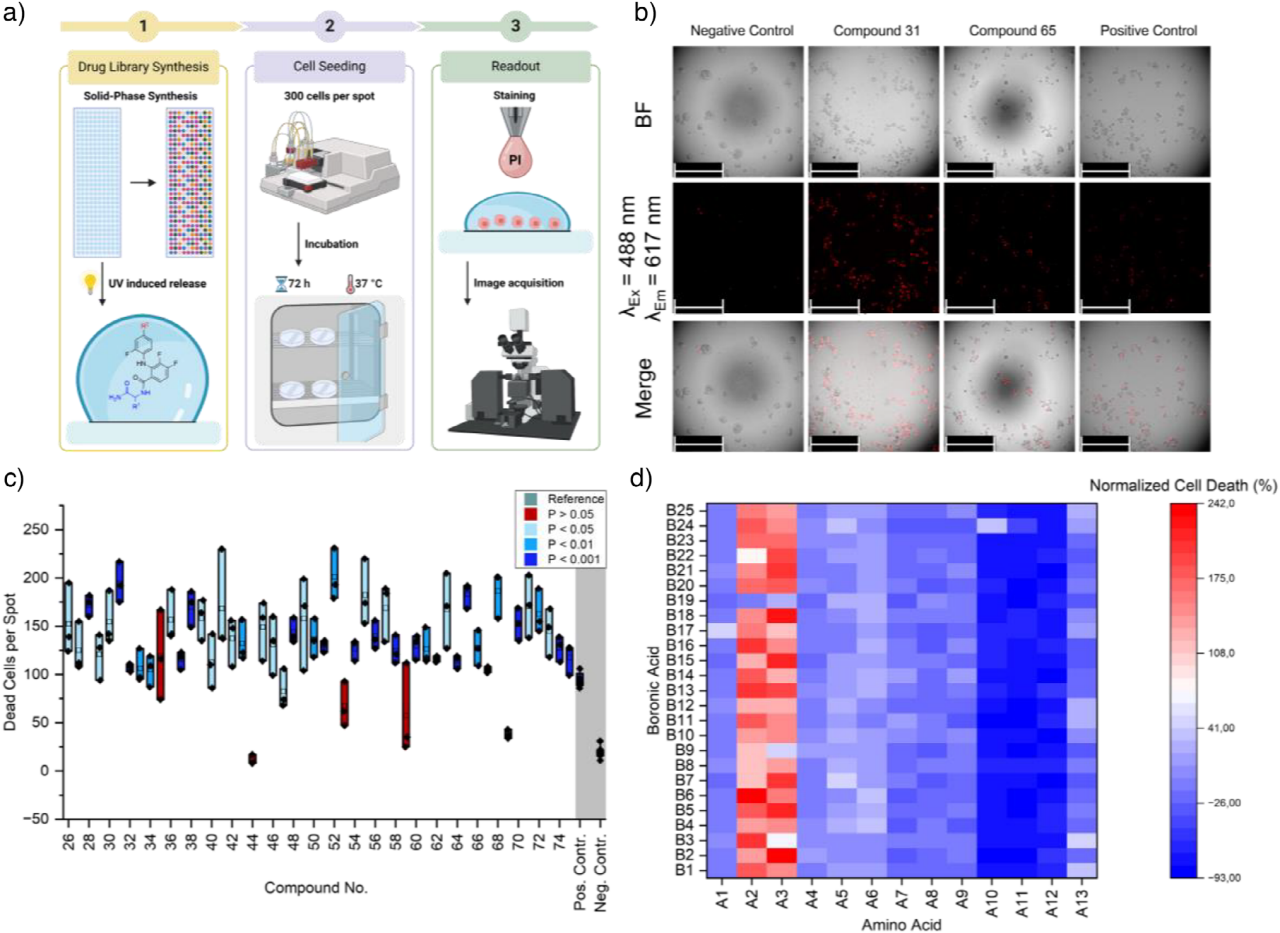
Biological screening of the synthesized library of MEK‐inhibitor compounds on the Nanodroplet Array. a) Schematic workflow of the screening, 1) the library is synthesized on the droplet microarray (with spots d = 1.4 mm) and released by UV irradiation. 2) 300 cells are seeded per spot and the slides are placed into a petri dish to incubate the cells for 72 h at 37 °C. 3) The cells are stained using propidium iodine (PI) and readout by fluorescence microscopy. b) Representative images acquired in the screening. The cells are imaged using the bright field microscope and excited at 488 nm. Dead cells emit at 617 nm. By merging the images, the amount of dead cells per spot is analyzed. The scale bar represents 250 µm. c) Box plot of the screening results showing the absolute number of dead cells per spot of three replicates of compounds 26–74 (see Figure S15 for the structures) with comparison to the positive control mirdametinib and negative control of vehicle‐treated cells. Colors indicate significance of difference between samples and negative control. Significance was calculated using unpaired, two‐tailed *t*‐test. d) Heat map of the normalized cell death using different combinations of boronic acid or amino acid building blocks. The cell death rate was normalized to the amount of dead cells using the positive control, mirdametinib.

Applying similar screening conditions as described in a previous report, the library of compounds was synthesized on the Nanodroplet Array platform containing 320 spots with 1.4 mm diameter to screen them directly on the substrate with HT‐29 cells (human colon cancer susceptible to MEK‐inhibition) for their cytotoxic effect (Figure 5a).^[^ ^20^ ^]^ The library was split in five parts and prepared on different slides with three replicates for each compound and five replicates further for positive and negative control. The synthesis conditions were chosen according to the previous optimization of the surface loading to achieve an approximate assay concentration of 10 µM required for primary screenings. Ten micrometer mirdametinib was chosen as the positive control, while cells incubated in medium without MEK‐inhibitor compounds was used as vehicle control. Due to the optimized seeding conditions from the previous report, 300 cells per spot were seeded onto the photocleaved compounds. After incubation for 72 h, the dead cells were stained with propidium iodine and quantified using fluorescence microscopy (Figure 5b). Interestingly, most of the 46 MEK‐inhibitor compounds containing either alanine (AA_2_) or ß‐alanine (AA_3_) as the amino acid building block showed significantly higher cell death compared to that of the positive control (Figure 5c, overview of structures and complete results in SI Figures S15–S17). Among all compounds, **AA_2,3_‐FIBA‐BA_x_** were the most active showing three times the average number of dead cells per spot compared to that of the positive control (Figure 5d).

**Figure 6:**
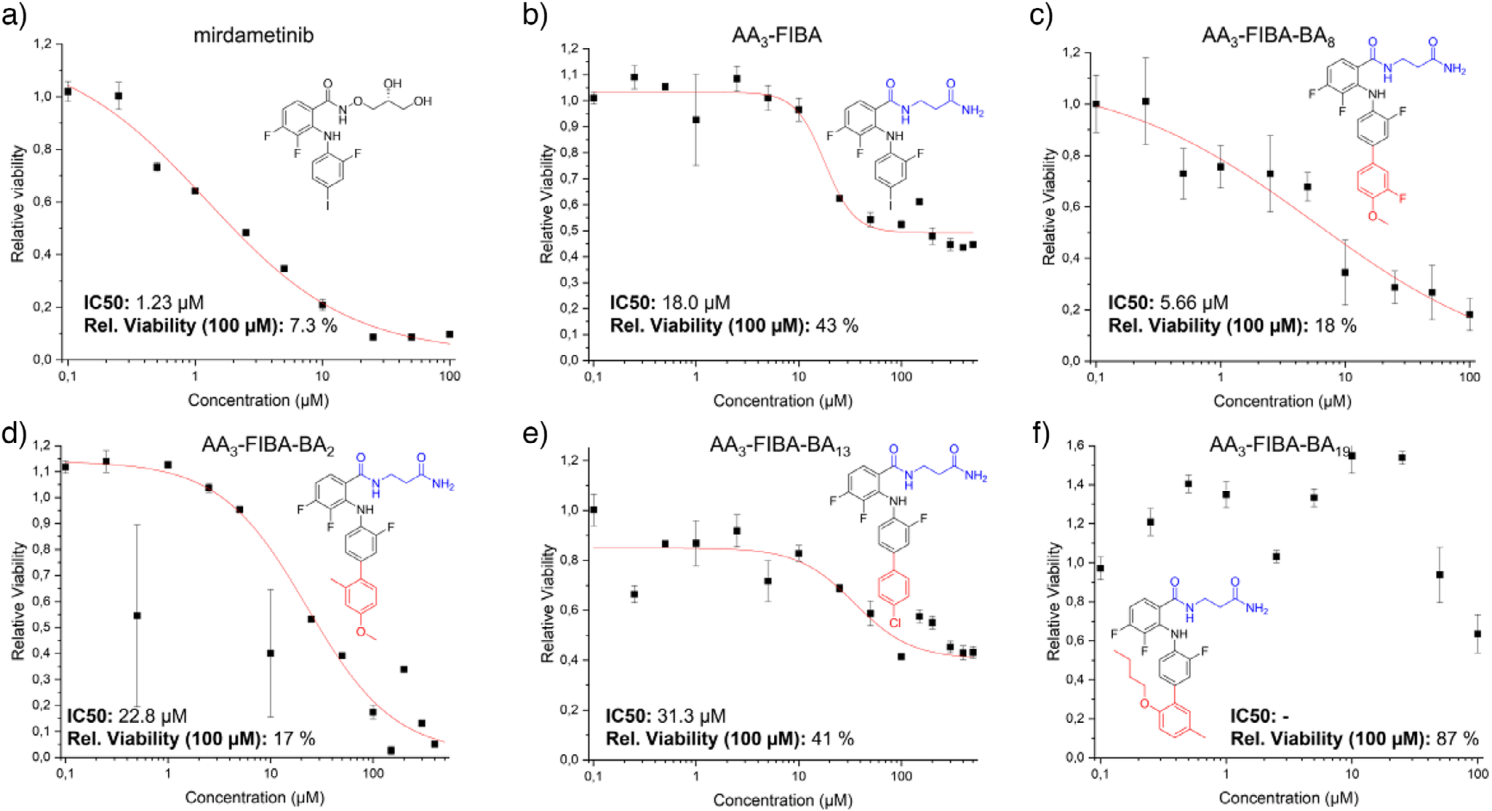
Determination of the potency of selected hit compounds. Results of the Cell TiterGlo assay showing the relative viability based on the concentration of different compounds measured in 384 well plates: a) mirdametinib, b) the IC 2 AA_3_‐FIBA, c) AA_3_‐FIBA‐BA_8_ (compound 58)_,_ d) AA_3_‐FIBA‐BA_2_ (compound 52), e) AA_3_‐FIBA‐BA_13_ (compound 63), f) AA_3_‐FIBA‐BA_19_ (compound 69). Values are the average of three replicates, error bars show the standard deviation. The IC_50_ values were calculated using the AAT Bioquest IC50 tool and the graphs were fitted using the obtained parameters.^[^ ^37^ ^]^

Notably, the relatively high variability observed across the three replicates limits the ability to assess the absolute potency. However, it is important to emphasize that each individual experiment encompassed the complete on‐chip workflow from compound synthesis, UV‐induced photocleavage, followed by the addition of cells via a liquid dispenser, incubation and staining for the viability analysis. While each step introduces potential sources of variability, the differences in cell viability between identified hit compounds and negative control were statistically significant (*p* < 0.05), demonstrating the platform's effectiveness for identifying active candidates in primary HTS. To validate the activity and assess the potency and efficiency compared to the positive control, seven compounds containing ß‐alanine were synthesized separately and tested on HT‐29 cells in a well plate. To keep the test conditions comparable to the conditions applied on the Nanodroplet Array substrate, the compounds were already added to each well before the initial seeding of the cells (Figure 5). Dose response measurements were performed using 100 nM to 100  or 500 µM concentration of mirdametinib, **AA_3_‐FIBA**, and the final MEK‐inhibitor compounds based on their solubility. The effect of the drug on cell viability was measured using the CellTiter‐Glo assay. The obtained measurements were normalized to vehicle‐treated cells, and the results were fitted to a dose‐response curve using the online tool from AAT Bioquest.^[^ ^37^ ^]^ The obtained IC_50_ values and observed relative viability at a concentration of 100 µM were then compared to the positive control mirdametinib (Figure 6a, fitting parameters in SI, Table S4).

The dose‐response curve of **AA_3_‐FIBA** (Figure 6b) showed an elevated IC_50_ value and relative viability compared to mirdametinib which confirms the influence of the carbonyl moiety on the activity. Taking a closer look at the chemical structures, **AA_3_‐FIBA‐BA_8_** (Figure 6c) and **AA_3_‐FIBA‐BA_2_** (Figure 6d) have both been modified similarly at the aromatic ring, but **AA_3_‐FIBA‐BA_8_** showed relatively low IC_50_ of 5.66 µM and **AA_3_‐FIBA‐BA_2_**—a reduced viability up to 17%. We observed that **AA_3_‐FIBA‐BA_13_** (Figure 6e) showed a drastically decreased potency with 41% relative viability at 100 µM concentrated drug treatment. Apart from the 4 active compounds chosen from the on‐chip screening to test for cell viability, we additionally synthesized and tested **AA_3_‐FIBA‐BA_19_** (Figure 6f) and confirmed the previous screening results on its inactivity. The results of the assay have shown a relative high viability throughout the tested range with 64% at the highest compound concentration tested. From these tests, we show that **AA_3_‐FIBA‐BA_8_** has comparable activity to that of the positive control and that the on‐chip screening can be used to identify potential MEK‐inhibitor compounds at scale. We hypothesize that the relatively reduced activity compared to that of compound screening performed on‐chip may stem from the incomplete conversion of the Suzuki coupling and lower purity of the on‐chip synthesized MEK inhibitors. Due to the incomplete reaction, each sample includes a mixture of the **Intermediate 1** and **2** compounds as well as covalently bound or adsorbed side products. This may lead to variations of active drug compounds per unit weight compared to the purified final compound synthesized in‐flask.

To further analyze the mechanism of action of the potential MEK‐inhibitors, molecular docking analysis for two selected compounds with the most promising biological assay results, **AA_3_‐FIBA‐BA_2_** and **‐BA_8_**, were carried out to predict ligand interaction with MEK1 and was compared to the interaction of mirdametinib and MEK1 by both calculated and published X‐ray diffraction (XRD) data.^[^ ^31^, ^38^ ^]^ The molecular docking results are shown in SI Figure S18. Firstly, we observed similar results for the proposed ligand interaction of mirdametinib with MEK1 through the calculations as compared to published XRD data, supporting the calculation method chosen. According to this, mirdametinib binds in the allosteric pocket in three binding regions containing the residues Lys97, Leu115, Leu118, Val127, Met143, Leu215, Ile216, and Met219. The proximity to these amino acids and the molecule's orientation is well reflected in the 2D projection of the binding situation. The library compounds **AA_3_‐FIBA‐BA_2_** and **‐BA_8_** were calculated to dock the binding pocket similar to mirdametinib, however, the simulation has shown a distinctively different orientation with partially altered interactions toward the residues on the inside. Hence, based on these results, we hypothesize that our library of synthesized compounds binds the target by the same mechanism as mirdametinib, namely as an allosteric non‐competitive ATP inhibitor. While most of the FDA‐approved kinase inhibitors are dominated by ATP‐competitive mechanisms, allosteric inhibitors offer a higher selectivity and are more resilient to the development of chemoresistance, for example because of point mutations.^[^ ^39^ ^]^ Additionally, the slightly changed orientation of the discovered compounds suggests that they might possess different tolerance for gained structural changes.

In conclusion, this study demonstrates a high‐throughput, scalable approach for synthesis and cell‐based screening small‐molecule drug candidates using a nanodroplet array platform. Compared to traditional microtiter plate (MTP)‐based screening technologies, such as 384‐ or 1536‐well plates, our nanodroplet array platform demonstrates several key advantages in resource conservation and efficiency as well as analytical compatibility. Conventional MTP systems typically require reaction and assay volumes ranging from 10 µL (1536‐well) to 30 µL (384 low‐volume well), which significantly increases the consumption of reagents, solvents, and cells.^[^ ^40^ ^]^ In contrast, our surface platform operates with wall‐less droplets allowing a miniaturized scale of 200 nL per spot, reducing volume and material requirements. While MTPs are compatible with LC‐MS and ESI‐MS for analysis, they are generally not compatible with MALDI‐MS, which limits their ability to perform spatially resolved, label‐free compound verification on‐chip and requires transfer of samples. Additionally, MTP have a fixed formats while the nanodroplet arrays allow for greater modularity in layout design and parallelization of workflows. These features enable a process integration with broad applicability across different stages of early drug discovery pipeline. Here, we synthesized 325 novel compounds via solid‐phase synthesis, characterized key intermediates by LC‐MS, and confirmed final product formation directly on the substrate using MALDI‐MSI. Additional on‐chip screening using HT‐29 colon cancer cells identified 46 promising inhibitors with higher cytotoxicity than FDA‐approved mirdametinib. A highlight of this platform is its efficient resource utilization by avoiding physical solid borders used in high‐throughput MTPs where miniaturization below 5 µL is challenged through adhesive forces.^[^ ^41^ ^]^ Using our platform into the nanoliter range, only 30 µg reactant per spot and <10 mg total reactants for the entire compound library were required. Moreover, on‐chip synthesis, analysis, and screening were seamlessly integrated, reducing the process time from more than 8 weeks [traditional Design Make Test Analysis (DMTA) methods] to just 7 days. The reaction pathway exhibited broad tolerance for various building blocks, producing compounds with sufficient purity for biological evaluation.

Thus, the nanodroplet array is a modular and scalable platform that enables both rapid synthesis and screening of potential inhibitors with minimal sample consumption, making it adaptable to larger libraries (>100 000 compounds) and diverse biological assays. Notably, the integration of synthesis with cell‐based screening in a miniaturized format eliminates unnecessary steps such as compound transfer from flasks to vials, storage in expensive facilities, and subsequent transfer to microtiter plates, thereby further reducing the environmental impact of the early drug discovery pipeline.

## Experimental Section

### General

All commercial reagents were used without further purification. All solvents in reagent grade or HPLC grade were used without purification. The glass slides in the size of 76 × 26 × 1 mm were purchased from Schott Nexterion (Jena, Germany).

### NMR Measurements

^1^H and ^13^C NMR spectra were recorded on Bruker Ascend DRX 400 NMR spectrometer at 400 MHz for ^1^H NMR and 101 MHz for ^13^C NMR at 298 K. Chemical shifts (ppm) are reported relative to TMS or the solvent peak.

### Low‐Volume Dispensing

Liquid dispensing below 1 µL was performed using a Gyger Certus Flex liquid dispenser equipped with an eight‐channel head. For dispensing of reagent solutions, Gyger SMLD 300 G microvalves (Ø 0.1/0.03 mm) with filter were used. Dispensing of cell suspensions was performed with Gyger SMLD 300 G microvalves (Ø 0.15/0.06 mm, no filter).

### Mass Spectrometry

LC‐MS measurements were performed on an Agilent 1260 Infinity II system consisting of a quaternary pump (GB7111B), autosampler (G7129A, 100 µL sample loop), a temperature‐controlled column oven (G7114A) and a variable UV‐VIS detector (G7114 A, VWD, flow cell G7114A 018, d = 10 mm, V = 14 µL). Separation was performed on a C18 HPLC‐column (Agilent Poroshell 120 EC‐C18 4.6x100 mm, 2.7 µm) operating at 40 °C. A gradient of ACN:H_2_O 10:90 – 80:20 *v/v* (additive 10 mmol L^−1^ NH_4_CH_3_CO_2_) at a flow rate of 1 mL·min^−1^ during 15 min was used as the eluting solvent. The flow was directed into an Agilent MSD (G6136BA, AP‐ESI ion source). The instrument was calibrated in the m/z range 118–2121 in the positive mode and 113–2233 in the negative using a premixed calibration solution (Agilent). The following parameters were used: spray chamber flow: 12 L min^−1^; drying gas temperature: 350 K, Capillary Voltage: 3000 V, Fragmentor Voltage: 100 V. All above Spectra were processed using ACDLabs Spectrus (2019.2.2). Exemplary LC‐MS spectra of library compounds 20, 243, 295, and 320 are depicted in Figure S19.

On‐Chip MALDI‐MS imaging was performed using a timsTOF fleX mass spectrometer (Bruker Daltonics GmbH, Bremen, Germany). To this end, 42.0 nmol of N‐(1‐naphthyl) ethylenediamine dihydrochloride (NEDC) matrix was deposited automatically on each 900 µm spot followed by MALDI MSI. MALDI MSI measurements were performed in negative ion mode with a lateral step size and laser spot size of 100 µm each, 400 laser shots per pixel and a repetition rate of 10 000 Hz, with a laser intensity of 75%. The “focus pre TOF” parameters “prepulse storage” and “transfer time” were set to 10 and 110 µs, respectively. The collision cell RF was set to 25 000.0 Vpp, with a collision cell energy offset of ‐10 V. Funnel 0 RF was set to 200 Vpp and Funnel 1 Rf to 350 Vpp. Prior to MSI data acquisition, external mass calibration was performed via the electrospray ionization source using ESI‐Low Concentration Tuning Mix (Agilent Technologies, Santa Clara, USA) and an enhanced quadratic calibration model. Ion images obtained via MALDI‐MS imaging were analyzed with SCiLS Lab (Bruker, USA). Exemplary mass spectra are depicted in Figures S20–S24.

### MALDI‐TOF‐MS on Ground Steel Target Plates

The measurement was performed on a rapifleX MALDI TOF system (Bruker Daltonics GmbH, Bremen, Germany). The tested compounds were synthesized in duplicates, extracted from the *poly*(Hema‐*co*‐Edma) slides using 1 µL of DMF/Water (2:1) per spot, and transferred onto a 384 ground steel MALDI target plate (Bruker Daltonics GmbH, Bremen, Germany). NEDC matrix [7 mg mL^−1^ NEDC in 70% MeOH:H_2_O (v:v)] was sprayed onto the target plate using an HTX M5 sprayer (HTX Tech., Chapel Hill, North Carolina, USA). Mass spectra were recorded in reflector negative ion mode covering a mass range of m/z 250 to m/z 1000. The laser power was set to 40% with a repetition rate of 10 kHz. The laser field size was set to diameter of 104 µm (Smartbeam M5 defocus in “MS dried droplet” mode). For each spot, 4000 shots were accumulated using “random walk” with 50 laser shots per raster spot. Mass calibration was performed using clusters of red phosphorus by a quadratic correction.

### MS/MS Measurements

Measurements were performed on a timsTOF fleX mass spectrometer equipped with a smartbeam 3D 10 kHz laser and TimsControl 4.1/5.1 and flexImaging v7.2/v7.4 software (Bruker Daltonics GmbH, Bremen, Germanz). Data was acquired in negative ion mode with 4000 random laser shots per spot (spot size of 2000 µm) in *imaging 20 µm* mode. For the m/z range of 80–1100, the *Ion Transfer parameters* were as follows: MALDI Plate Offset ‐170 V, Deflection 1 Delta 0 V, Funnel 1 RF 419 Vpp, isCID Energy ‐0.0 V, Funnel 2 RF 350 Vpp, and Multipole RF 100 Vpp. The *Focus Pre‐TOF parameters* were set as follows: transfer Time 85 µs, and Pre Pulse Storage 10 µs. The *collision cell parameters* were set to a collision RF of 750 Vpp and collision energies of 35, 35, 40, 45, and 45 V for m/z 472.16 (Compound 1, AA_1_‐FIBA‐BA_1_), 523.12 (Compound 122, AA_5_‐FIBA‐BA_22_), 580.17 (Compound 166, AA_7_‐FIBA‐BA_16_), 788.25 (Compound 190, AA_8_‐FIBA‐BA_15_), and 683.23 (Compound 208, AA_9_‐FIBA‐BA_8_), respectively. An isolation window of m/z ± 1 was used, with the quadrupole Ion Energy set to 5 eV. Liquid dispensing at or below 1 µL was performed using a Gyger Certus Flex liquid dispenser equipped with an eight‐channel head. Recorded spectra were further compared to determine fragmentation patterns.^[^ ^42^ ^]^

### UV‐Induced Reactions

UVA‐ or UVC intensities of all lamps were measured with a UV‐Meter (Dr. Hönle AG, Gräfelfing, Germany) using an UVA‐ or UVC‐sensor respectively. For photolithographic manufacture of patterned slides through Thiol‐yne reaction a UVA Cube 2000 (Dr. Hönle AG, Gilching, Germany) was used. Photoinduced cleavage of compounds off the solid‐phase was done using an Biolink BLX UV chamber (Witec, Sursee, Switzerland) at 365 nm. The UV absorbance was measured by The SpectraMax iD3‐4170 Multi‐Mode Microplate Readers.

### Preparation of the Nanodroplet Array

The preparation and attachment of the photolinker were done as described previously.^[^ ^20^ ^]^ *Modification*: An uncoated Nexterion glass slide (Schott AG, Mainz, Germany) was activated by a UV/Ozone cleaner (Jetlight Co. Inc., Irvine, California, USA) for 10 min. Then, 150 µL of modification mixture [20% 3‐(trimethoxysilyl)propyl methacrylate in ethanol] was applied on the slide and covered with another activated glass slide. The solution was evenly distributed between these two slides and all the bubbles were removed. The slides were left to react for 30 min. These functionalization steps were repeated two times, followed by washing with acetone and drying with nitrogen gun.

### Polymerization

Nanoporous poly(2‐hydroxyethyl methacrylate‐*co*‐ethylene dimethacrylate (HEMA‐co‐EDMA) polymerization mixture was prepared with following components: 24 wt% 2‐hydroxyethyl methacrylate (HEMA), 16 wt% ethylene dimethacrylate (EDMA), 12 wt% 1‐decanol, 48 wt% cyclohexanol, and 0.4 wt% 2,2‐dimethoxy‐2‐phenylacetophenone (DMPAP). Thirty microliter of polymerization mixture were pipetted on a fluorinated glass slide, placed with a modified glass slide on top allowing the mixture to spread without formation of air bubbles. The sandwich‐like slides were put under the UV lamp (260 nm wavelength) and irradiated for 20 min with 10 mW cm^−2^ intensity. After that, the slides were separated and washed with ethanol, followed by immersing the substrate in ethanol for several hours.

### Esterification

The esterification solution was prepared in a 50 mL falcon tube by mixing 45 mL of acetone, 56.0 mg of 4‐(dimethylamino)pyridine and 111.6 mg of 4‐pentynoic acid. The slides with poly(HEMA‐*co*‐EDMA) surfaces were immersed in the solution and cooled at ‐20 °C for 15 min. Then, 180 µL of N,N'‐diisopropylcarbodiimide was added to the solution, and the tube was fixed on a shaker for overnight at 200 rpm. After esterification, the slide was immersed in ethanol for 2 h to remove unreacted chemicals.

### Patterning

In a dark room, 200 µL of the perfluorodecanethiol solution (10% 1*H*,1*H*,2*H*,2*H*‐perfluorodecanethiol in 2‐propanol) was pipetted on the alkyne substrate and covered with a photomask (round spots with diameter of 900 µm, 18 x 64 spots per slide, round spots with diameter of 1.4 mm, 10 x 32 spots per slide; or round spots with diameter of 2.8 mm, 5 x 16 spots per slide). After irradiation with UV light at 10 mW cm^−2^ for 3 min, the substrate was washed and dried. Then, 200 µL of the hydrophilic thiol solution (15 wt% cysteamine HCl in a 1:1 water:ethanol solution) was pipetted on the substrate and covered with a fluorinated quartz slide. Next, it was again irradiated with 260 nm UV light at for 3 min, washed with water/ethanol and dried with an air gun.

ITO‐coated glass slides (Diamonds Coatings, West Midlands, UK) were activated first, by submerging in a solution of hydrogen peroxide in water (30%, v/v) for 45 min. The slides were afterwards washed with water and acetone, dried with nitrogen flow and then submerged in a solution of 3‐(Trimethoxysilyl)propylmethacrylate (3%, v/v) in toluene. The mixture was heated to 80 °C overnight, the slides were removed from the solution and washed with acetone. After drying, the slides were used like normal glass slides for the preparation of polymer films.

### Photolinker Modification

Fmoc‐Photolinker at certain concentration, diisopropyl carbodiimide (DIC, 10%, v/v) and 1‐hydroxybenzotriazol (HOBT, same concentration as Fmoc‐Photolinker) were dissolved in N‐methyl‐2‐pyrrolidone (NMP) to prepare the photolinker solution. To immobilize the linker onto the surface of the hydrophilic spots, certain volume (300 nL for 1.4 mm pattern and 3 µL for 2.8 mm pattern, 150 nL for 900 µm pattern) of photolinker solution was dispensed. Then, the slides were placed in a Petri dish and protected from light. The reaction was carried out overnight at rt, followed by washing with ethanol/acetone, and drying with nitrogen flow. The unreacted free amines on the surface were capped with 10% vol pyridine in acetic anhydride for 10 min at rt. The slides were again washed and dried. To deprotect the photolinker, the whole slide was submerged in a solution of piperidine in DMF (20%, v/v) for 30 min. Afterwards, the slide was washed with acetone and used directly.

### Coupling of the Amino Acid onto the Photolinker

The hydrophilic spots of a Nanodroplet Array modified with photolinker solution were treated with a solution of Fmoc‐amino acid and HOBT (equimolar amount) in a mixture of NMP/DIC (9:1, v/v) (150 nL for 900 µm spots, 300 nL for 1.4 mm spots, 3 µL for 2.8 mm spots). The reaction proceeded overnight, then the solution was washed off with acetone and the slide was dried with nitrogen flow. Slides modified with surface bound aminoacids were submerged in a solution of piperidine in DMF (20%, v/v) for 1 h. Afterwards, the slides were washed with acetone and dried under nitrogen flow.

### Coupling of the Core Structure FIBA onto the Amino Acid

The hydrophilic spots of a Nanodroplet Array modified with deprotected aminoacids were treated with a solution of 3,4‐Difluoro‐2‐(2‐fluoro‐4‐iodophenylamino)benzoic acid (**4**) and HOBT (100 mM, respectively) in a mixture of NMP/DIC (9:1, v/v) (150 nL for 900 µm spots, 300 nL for 1.4 mm spots, 3 µL for 2.8 mm spots). The reaction proceeded overnight, then the solution was washed off with acetone and the slide was dried with nitrogen flow.

### Suzuki‐Coupling on the Core Molecule FIBA

To the hydrophilic spots of a Nanodroplet Array modified with aromatic iodides, a solution of sodium tetrachloropalladat(II) (0.2 M) in water (1.5 µL on 2.8 mm pattern, 60 nL on 1.4 mm pattern, 40 nL on 900 µm pattern) and a solution of *N,N*‐diisopropyl‐dibenzoylphosphinate (0.5 M) in NMP (1.5 µL on 2.8 mm pattern, 60 nL on 1.4 mm pattern, 40 nL on 900 µm pattern) were added and preincubated to form the catalytic active species for 15 min at rt. Afterwards, a solution of boronic acid (0.5 M) in NMP (3.0 µL on 2.8 mm pattern, 120 nL on 1.4 mm pattern, 80 nL on 900 µm pattern) and aqueous sodium carbonate solution (sat.) (2.0 µL on 2.8 mm pattern, 60 nL on 1.4 mm pattern, 40 nL on 900 µm pattern) are added and the reaction is conducted at rt over night. The slide is afterwards washed with water and ethanol, then submerged in a solution of potassium cyanide (0.1 M) in a mixture of DMSO/water (1:1, v:v) for 3 h. The slide is again washed with water and ethanol and then dried under nitrogen flow.

### Cleavage and Analysis of Compounds

To release the compounds off the solid phase for chemical analysis, DI water is added to each spot of the Nanodroplet Array (5 µL for 2.8 mm pattern, 150 nL for 1.4 mm pattern, 100 nL for 900 µm pattern) and the slide is enclosed in a petri dish. The slide is then irradiated using a Biolink BLX UV chamber (Witec, Sursee, Switzerland) at 365 nm (2.19 mW cm^−2^) for different times. Afterwards, the water is evaporated and the sample can be extracted. To the irradiated 2.8 mm spots of a Nanodroplet Array, 5 µL DMF were added and the liquid was collected. Then, 5 µL DI‐water were added to these spots, to dissolve any hydrophilic compounds, and combined with the organic phase. For every measurement, five repetitions of those 10 µL samples were collected into one, then 10 µL of this solution was injected for LC‐MS measurements. Standard compounds were prepared as stock solution (10 mM) in DMSO or acetonitrile and were then serial diluted with water to get different concentrations for the calibration. Calibration curves were recorded using the same HPLC‐ and MS‐methods as for the Nanodroplet Array samples and ranged from 10 to 10 000 µM.

### Cell‐Based Experiments

Dulbecco's Modified Eagle Medium (DMEM), fetal bovine serum (FBS), and penicillin‐streptomycin (P/S) were purchased from Gibco, Life Technologies Inc. Automated fluorescence microscopy of stained cells was performed with a Leica Thunder 3D Imager (Leica Microsystems, Germany). Cell images were analyzed using ImageJ.

The cell experiments were conducted with the adherent colon‐cancer cell line HT‐29 obtained from Prof. Veronieque Orian‐Rousseau (IBCS‐FMS, KIT). The cells were cultured in DMEM supplemented with 10% FBS and 1% penicillin/streptomycin (Pen/Strep) in a humidified incubator at 37 °C with 5% CO_2_ airflow.

### Biological Screening on the Nanodroplet Array

Library synthesis was carried out on Nanodroplet Arrays with 1.4 mm spots. After final synthesis step, the slide was washed with acetone and water, disinfected in 70% ethanol and then dried for 20 min under sterile conditions. It was placed in a petri dish filled with 2 mL PBS in the bottom. The cleavage medium (DMEM without phenolred, 1% P/S, no FBS, supplemented with 0.1% DMSO) was dispensed (700 nL for negative control, 350 nL for positive control and samples). The slide was irradiated in the closed petri dish with UVA light for 40 min to assure full cleavage. Then, 350 nL of a solution of mirdametinib in cleavage medium (28.6 µm) was added to the positive control spots. A humidifying pad soaked with 5 mL PBS was added to the lid, and the slide was incubated at 37 °C overnight. The cell suspension was prepared in the printing medium (DMEM without phenolred, with 30 % FBS and 1% P/S) at a concentration of 1*10^6^ cells per mL. Then, 300 nL of cell suspension were added to each spot using a Gyger Certus Flex. The slides were incubated for 72 h at 37 °C. Afterwards, 100 nL of a solution containing propidium iodide in PBS (10 µg mL^−1^) were added, the slide was incubate at 37 °C for 15 min, then the slides were imaged in brightfield and PI fluorescence channel using a Leica Thunder 3D Imager. All samples were produced in triplicates, positive controls (cells treated with 10 µM Mirdametinib) and negative controls (untreated cells) were produced with five replicates each.

### Cell Based Assay in 384‐Well Plate

For the assay, 15 µL of the tested compounds, as well as positive controls in medium [DMEM/DMSO 699:1 (v/v), +0.1% P/S] in different concentrations between 0.1 and 100 µm were added to the respective wells. Subsequently, 10 000 HT‐29 cells in medium (DMEM, +30% FBS, +0.1% P/S) were added to each well containing compounds, yielding a total volume of 25 µL per well. The plate was incubated at 37 °C for 72 h. Afterward, the plate was allowed to equilibrate to room temperature for 30 min. Subsequently, 25 µL of Cell TiterGlo reagent was added to each well, and the plate was incubated at room temperature for 10 min before being analyzed for luminescence using a CLARIOstar Plus plate reader.

### Determination of IC50

To obtain information about potency and efficacy of the tested compounds, obtained values were fitted using a four‐parameter logistic regression mode to the function:

$$y=Min+\frac{Max-Min}{1+{\left(\frac{x}{IC_{50}}\right)}^{Hill\;Coefficient}}$$

with y, the relative viability; x, the concentration; Max, the upper plateau value for the relative viability; Min, the lower plateau value for the relative viability; IC50, the position of the turning point; Hill Coefficient, a measure of the steepness of the curve.^[^ ^37^ ^]^

### Docking Simulations

The binding analysis was done using SwissDock.ch.^[^ ^38^ ^]^ Processing of the generated data and creation of 2D interaction‐maps was done using BIOVIA Discovery Studio Visualizer (v. 24.1.0.23298).

### Synthesis of Standard Compounds

(S)‐N‐(1‐amino‐1‐oxopropan‐2‐yl)‐3,4‐difluoro‐2‐((2‐fluoro‐4‐iodophenyl)amino)benzamide (**IC 2**): 500 mg 3,4‐Difluoro‐2‐(2‐fluoro‐4‐iodophenylamino)benzoic acid (763 µmol, 1.00 Eq.) and 238 mg alanine amide hydrochloride (1140 µmol, 1.50 Eq.) were dissolved in 5000 µL DMSO. 258 mg 1‐hydroxybenzotriazol (1140 µmol, 1.50 Eq.), 366 mg N‐(3‐Dimethylaminopropyl)‐N′‐ethyl‐carbodiimid hydrochloride (1140 µmol, 1.50 Eq.) and 420 µL N‐Methylmorpholine (385 mg, 2290 µmol, 3.00 Eq.) were added and the mixture was stirred overnight. The product was precipitated by addition of water, filtered, washed with water and then dried under vacuum to obtain the desired product **IC 2** as white solid (590 mg, 1.27 mmol, quant.). **^1^H‐NMR** (400 MHz, DMSO‐d_6_) δ [ppm]: 9.09 (s, 1H, N*H*Ar), 8.72 (d, 1H, CON*H*), 7.68–7.63 (m, 1H, H_Ar_), 7.61–7.55 (m, 1H, H_Ar_), 7.45 (s, 1H, NH_2_), 7.05 (s, 1H, NH_2_), 7.40–7.35 (m, 1H, H_Ar_), 7.25–7.16 (m, 1H, H_Ar_), 6.72–6.64 (m, 1H, H_Ar_), 4.36–4.29 (m, 1H, C*H*), 1.28 (d, 3H, CH_3_). **^13^C‐NMR** (101 MHz, DMSO‐d_6_) δ [ppm]: 174.43, 168.8, 154.06, 152.1, 133.56, 132.02, 131.45, 125.68, 124.22, 124.04, 122.96, 120.47, 110.17, 82.64, 49.24, 18.22. **ESI‐MS**: calc. for [M + H]^+^: 464.008; found: 464.008. The respective spectra are depicted in Figures S25–S27.

(S)‐N‐(1‐amino‐1‐oxopropan‐2‐yl)‐3,4‐difluoro‐2‐((3‐fluoro‐[1,1′‐biphenyl]‐4‐yl)amino)benzamide (**IC 3**): A solution of 2.5 mg palladium(II)acetate (0.002 mmol, 0.05 Äq.) and 12.0 mg triphenylphosphine (0.009 mmol, 0.25 Äq.) in 5 mL THF/water (9:1, v/v) was stirred for 10 min under argon atmosphere. 25.3 mg phenylboronic acid (0.208, 1.20 Eq.), 80.0 mg precursor **IC 2** (0.173 mmol, 1.00 Eq), and 72.0 mg potassium carbonate (0.519 mmol, 3.00 Eq.) were added carefully, afterwards the mixture was stirred under reflux for 16 h. The solvent was removed under reduced pressure and the crude is purified via column chromatography (Silica, DCM → DCM:MeOH, v/v 9:1) to obtain target compound as beige powder (28.5 mg, 80%).**^1^H‐NMR** (400 MHz, DMSO‐d_6_) δ [ppm]: 9.25 (s, 1H, N*H*Ar), 8.79 (d, 1H, CON*H*), 7.75–7.68 (m, 3H, H_Ar_), 7.53–7.40 (m, 5H, H_Ar_), 7.30–7.18 (m, 1H, H_Ar_), 7.09 (s, 1H, NH_2_), 7.04–6.95 (m, 1H, H_Ar_), 4.45–4.35 (m, 1H, C*H*), 1.35 (d, 3H, CH_3_). **^13^C‐NMR** (101 MHz, DMSO‐d_6_) δ [ppm]: 174.21, 169.98, 154.82, 152.42, 139.13, 134.25, 130.38, 129.4, 127.7, 126.7, 125.8, 122.49, 119.0, 113.87, 113.67, 109.93, 109.7, 49.26, 18.22. **ESI‐MS**: calc. for [M + H]^+^: 414.143; found: 414.142. The respective spectra are depicted in Figures S28–S30.

### Individual Synthesis of Hit‐Compounds

N‐(3‐amino‐3‐oxopropyl)‐3,4‐difluoro‐2‐((2‐fluoro‐4‐iodophenyl)amino)benzamide (**AA_3_‐FIBA**): 500 mg 3,4‐Difluoro‐2‐(2‐fluoro‐4‐iodophenylamino)benzoic acid (763 µmol, 1.00 Eq.) and 238 mg beta‐alanine amide hydrochloride (1140 µmol, 1.50 Eq.) were dissolved in 5000 µL DMSO. 258 mg 1‐hydroxybenzotriazol (1140 µmol, 1.50 Eq.), 366 mg N‐(3‐Dimethylaminopropyl)‐N′‐ethyl‐carbodiimid hydrochloride (1140 µmol, 1.50 Eq.), and 420 µL N‐Methylmorpholine (385 mg, 2290 µmol, 3.00 Eq.) were added and the mixture was stirred overnight. The product was precipitated by addition of water, filtered, washed with water, and then dried under vacuum to obtain the desired product as white solid (592 mg, 1.27 mmol, quant.). **^1^H‐NMR** (400 MHz, d_6_‐DMSO) δ [ppm]: 9.32 (s, 1H, NH_Ar_), 8.77 (t, 1H, CON*H*), 7.64–7.51 (m, 2H, H_Ar_), 7.45–7.30 (m, 2H, H_Ar_), 7.25–7.12 (m, 1H, H_Ar_), 6.83 (s, 2H, NH_2_), 6.76–6.66 (m, 1H, H_Ar_), 3.44–3.36 (m, 2H, CH_2_), 2.32 (t, 2H, CH_2_). **^13^C‐NMR** (101 MHz, d_6_‐DMSO) δ [ppm]: 172.81, 167.28, 154.41, 151.94, 133.66, 132.42, 131.15, 125.25, 124.24, 124.04, 122.31, 120.70, 110.14, 82.83, 36.46, 35.00. **ESI‐MS** calc. for [M + H]^+^ 464.008, found: 463.950; The respective spectra are depicted in Figures S31–S33.

General procedure for the Suzuki‐reaction of compound **AA_3_‐FIBA**: A solution of 2.5 mg palladium(II)acetate (0.002 mmol, 0.05 Äq.) and 12.0 mg triphenylphosphine (0.009 mmol, 0.25 Eq.) in 5 mL THF/water (9:1, v/v) was stirred for 10 min under argon atmosphere. 0.208 mmol of a boronic acid (1.20 Eq.), 80.0 mg precursor **AA_3_‐FIBA** (0.173 mmol, 1.00 Eq) and 72.0 mg potassium carbonate (0.519 mmol, 3.00 Eq.) were added carefully, afterwards the mixture was stirred under reflux for 16 h. The solvent was removed under reduced pressure and the crude is purified via column chromatography (Silica, DCM → DCM:MeOH, v/v 9:1) to obtain target compound as beige powder with varying yield.

N‐(3‐amino‐3‐oxopropyl)‐3,4‐difluoro‐2‐((3‐fluoro‐4′‐methoxy‐2′‐methyl‐[1,1′‐biphenyl]‐4‐yl)amino)benzamide (compound **52**): 4‐Methoxy‐2‐methylphenylboronic acid was used as boronic acid. Yield: 71%. **^1^H‐NMR** (400 MHz, d_6_‐DMSO) δ [ppm]: 9.45 (s, 1H, NH_Ar_), 8.83 (t, 1H, CON*H*), 7.64–7.55 (m, 1H, H_Ar_), 7.40 (s, 2H, NH_2_), 7.25–7.16 (m, 3H, H_Ar_), 7.09–7.02 (m, 1H, H_Ar_), 7.01–6.93 (m, 1H, H_Ar_), 6.92–6.84 (m, 3H, H_Ar_), 3.82 (s, 3H, OCH_3_), 3.50–3.43 (m, 2H, CH_2_), 2.39 (t, 2H, CH_2_), 2.29 (s, 3, CH_3_). **^13^C‐NMR** (101 MHz, d_6_‐DMSO) δ [ppm]: 172.83, 167.51, 158.93, 151.75, 136.69, 135.40, 132.91, 131.08, 129.24, 125.58, 121.72, 118.59, 116.45, 116.19, 111.92, 109.54, 55.54, 36.50, 35.05, 20.95. **ESI‐MS** calc. for [M + H] 458.133, found: 458.132. The respective spectra are depicted in Figures S34–S36.

N‐(3‐amino‐3‐oxopropyl)‐3,4‐difluoro‐2‐((3‐fluoro‐3′‐isopropyl‐[1,1′‐biphenyl]‐4‐yl)amino)benzamide (compound **54**): 3‐Isopropylphenylboronic acid was used as boronic acid. Yield: 62%. **^1^H‐NMR** (400 MHz, d_6_‐Aceton) δ [ppm]: 9.40 (s, 1H, NH_Ar_), 8.00 (s, 1H, CONH), 7.51–7.44 (m, 1H, H_Ar_), 7.40 (s, 1H, H_Ar_), 7.36–7.29 (m, 2H, H_Ar_), 7.28–7.19 (m, 2H, H_Ar_), 7.13–7.07 (m, 1H, H_Ar_), 6.92–6.84 (m, 2H, NH_2_), 6.78 (s, 1H, H_Ar_), 6.17 (s, 1H, H_Ar_), 3.51–3.44 (m, 2H, CH_2_), 2.89–2.81 (m, 1H, CH), 2.38 (t, 2H, CH_2_), 1.15 (d, 6H, CH_3_). **^13^C‐NMR** (101 MHz, d_6_‐Aceton) δ [ppm]: 172.98, 167.41, 155.08, 153.95, 152.68, 149.47, 144.18, 141.71, 139.51, 135.45, 133.56, 130.01, 128.87, 127.13, 125.25, 124.62, 123.97, 118.87, 113.48, 113.29, 108.83, 108.65, 36.08, 34.07, 23.42. **ESI‐MS** calc. for [M + H]+ 456.190, found: 456.150. The respective spectra are depicted in Figures S37–S39.

N‐(3‐amino‐3‐oxopropyl)‐2‐((3,3′‐difluoro‐4′‐methoxy‐[1,1′‐biphenyl]‐4‐yl)amino)‐3,4‐difluorobenzamide (compound **58**): 3‐Fluor‐4‐methoxyphenylboronic acid was used as starting material. Yield: 62%. **^1^H‐NMR** (400 MHz, d_6_‐DMSO) δ [ppm]: 9.42 (s, 1H, NH_Ar_), 8.79 (s, 1H, CONH), 7.58 (s, 1H, H_Ar_), 7.55 (s, 2H, NH_2_), 7.49–7.44 (m, 1H, H_Ar_), 7.40–7.32 (m, 2H, H_Ar_), 7.25–7.11 (m, 2H, H_Ar_), 6.98–6.92 (m, 1H, H_Ar_), 6.83 (s, 1H, H_Ar_), 3.87 (s, 3H, CH_3_), 3.45–3.38 (m, 2H, CH_2_), 2.34 (t, 2H, CH_2_). **^13^C‐NMR** (101 MHz, d_6_‐DMSO) δ [ppm]: 172.84, 167.45, 154.90, 153.46, 152.50, 151.04, 147.00, 146.89, 141.68, 132.95, 132.20, 125.20, 122.71, 122.46, 121.83, 119.20, 114.69, 114.21, 114.37, 56.55, 36.48, 35.04. **ESI‐MS** calc. for [M + H]+ 462.144, found: 462.050. The respective spectra are depicted in Figures S40–S42.

N‐(3‐amino‐3‐oxopropyl)‐2‐((4′‐chloro‐3‐fluoro‐[1,1′‐biphenyl]‐4‐yl)amino)‐3,4‐difluorobenzamide (compound **63**): 4‐Chlorophenylboronic acid was used as boronic acid. Yield: 52%. **^1^H‐NMR** (400 MHz, d_6_‐DMSO) δ [ppm]: 9.45 (s, 1H, NH_Ar_), 8.81 (t, 1H, CONH), 7.88–7.75 (m, 1H, H_Ar_), 7.72 (d, 2H, NH_2_), 7.65–7.55 (m, 2H, H_Ar_), 7.51 (d, 2H, H_Ar_), 7.43 (m, 1H, H_Ar_), 7.37 (s, 1H, H_Ar_), 7.26–7.18 (m, 1H, H_Ar_), 7.03–6.96 (m, 1H, H_Ar_), 3.47–3.42 (m, 2H, CH_2_), 2.36 (t, 2H, CH_2_). **^13^C‐NMR** (101 MHz, d_6_‐DMSO) δ [ppm]: 172.82, 167.40, 154.77, 152.37, 137.96, 135.81, 135.10, 132.88, 132.51, 130.99, 129.31, 128.42, 125.25, 122.90, 119.07, 118.62, 117.73, 113.70, 109.93, 36.48, 35.02, 30.07. **ESI‐MS** calc. for [M + H]+ 448.104, found: 448.000. The respective spectra are depicted in Figures S43–S45.

N‐(3‐amino‐3‐oxopropyl)‐2‐((2′‐butoxy‐3‐fluoro‐5′‐methyl‐[1,1′‐biphenyl]‐4‐yl)amino)‐3,4‐difluorobenzamide (compound **69**): 2‐Butoxy‐5‐methylphenylboronic acid was used as boronic acid. Yield: 52%. **^1^H‐NMR** (400 MHz, d_6_‐DMSO) δ [ppm]: 9.42 (s, 1H, NH_2_), 8.79 (m, 1H, CONH), 7.59–7.53 (m, 1H, H_Ar_), 7.40–7.33 (m, 2H, NH_2_), 7.24–7.07 (m, 4H, H_Ar_), 7.00–6.88 (m, 2H, H_Ar_), 6.83 (s, 1H, H_Ar_), 3,95 (t, 2H, CH_2_), 3.47–3.39 (m, 2H, CH_2_), 2.35 (t, 2H, CH_2_), 2.28 (s, 3H, CH_3_), 1.68–1.59 (m, 2H, CH_2_), 1.45–1.33 (m, 2H, CH_2_), 0.89 (t, 3H, CH_3_). **^13^C‐NMR** (101 MHz, d_6_‐DMSO) δ [ppm]: 172.84, 167.53, 153.84, 151.60, 133.20, 132.53, 131.10, 129.93, 129.47, 128.53, 125.42, 125.25, 125.16121.63, 118.34, 116.45, 116.25, 113.35, 109.51, 109.33, 68.15, 36.50, 35.05, 31.25, 20.55, 19.26, 14.06. **ESI‐MS** calc. for [M + H]+ 500.216, found: 500.150. The respective spectra are depicted in Figures S46–S48.

N‐(3‐amino‐3‐oxopropyl)‐3,4‐difluoro‐2‐((3‐fluoro‐4′‐hydroxy‐[1,1′‐biphenyl]‐4‐yl)amino)benzamide (compound **73**): 4‐Hydroxyphenylboronic acid was used as boronic acid. Yield: 20%. **^1^H‐NMR** (400 MHz, d_6_‐DMSO) δ [ppm]: 9.52 (s, 1H, NH_Ar_), 9.40 (s, 1H, OH_Ar_) 8.78 (t, 1H, CONH), 7.55 (t, 1H, H_Ar_), 7.49 (s, 1H, H_Ar_), 7.47 (s, 2H, NH_2_), 7.35 (s, 1H, H_Ar_), 7.32–7.28 (m, 1H, H_Ar_), 7.18–7.11 (m, 1H, H_Ar_), 6.98–6.9 (m, 1H, H_Ar_), 6.85–6.8 (m, 3H, H_Ar_), 3.4 (m, 2H, CH_2_), 2.34 (t, 2H, CH_2_). **^13^C‐NMR** (101 MHz, d_6_‐DMSO) δ [ppm]: 172.83, 167.53, 157.49, 155.09, 152.69, 135.01, 133.36, 129.95, 129.14, 127.81, 121.99, 121.46, 119.53, 116.17, 113.09, 112.89, 109.32, 109.15, 36.48, 35.04. **ESI‐MS** calc. for [M + H]+ 430.138, found: 430.050. The respective spectra are depicted in Figures S49–S51.

## Conflict of Interests

The authors declare no conflict of interest.

## Supporting information



Supporting information

## Data Availability

The data that support the findings of this study are available from the corresponding author upon reasonable request.
