# Supplementary material for: Nanodroplet Array Platform for Integrated Synthesis and Screening of MEK Inhibitors: a Miniaturized Approach to Early Drug Discovery
Source: Angew Chem Int Ed Engl. 2025 Aug 18;64(48):e202507586. doi: 10.1002/anie.202507586 (PMC12643352; doi:10.1002/anie.202507586)
Supplement: Supplementary file 1 — Supporting information [file ANIE-64-e202507586-s001.docx]

**Supporting Information**

**Nanodroplet Array Platform for Integrated Synthesis and Screening of MEK Inhibitors: A Miniaturized Approach to Early Drug Discovery**

**M. Seifermann^§[1]^, J. Höpfner^§[1]^, L. Bauer^§[1]^, D. Varadharajan^[2]^, S. Schmidt^[3]^, B. Fröhlich^[3]^, B. Wellenhofer^[1]^, C. Luchena^[1]^, C. Hopf^[3][4][5]^, A. A. Popova^[1]^, P. A. Levkin^[1][6]^***

[1] Institute of Biological and Chemical Systems-Functional Molecular Systems (IBCS-FMS), Karlsruhe Institute of Technology (KIT), Hermann-von Helmholtz-Platz 1, Eggenstein-Leopoldshafen, 76344, Germany

[2] Scivalon, Bruckenäcker 9, Stuttgart, 70565, Germany

[3] Center for Mass Spectrometry and Optical Spectroscopy (CeMOS), Technische Hochschule Mannheim, Paul-Wittsack-Straße 10, Mannheim, 68163, Germany

[4] Medical Faculty, Heidelberg University, Im Neuenheimer Feld 280, Heidelberg, 69117, Germany

[5] Mannheim Center for Translational Neuroscience (MCTN), Medical Faculty Mannheim, Heidelberg University, Theodor Kutzer-Ufer 1-3, Mannheim, 68167, Germany

[6] Institute of Organic Chemistry, Karlsruhe Institute of Technology, Fritz-Haber-Weg 6, Karlsruhe, 76131, Germany

# **Overview and annotation of synthesized library compounds**

**Table S1**. Overview of all 325 MEK-inhibitor compounds using various combinations of amino acid and boronic acid as the modular building blocks.


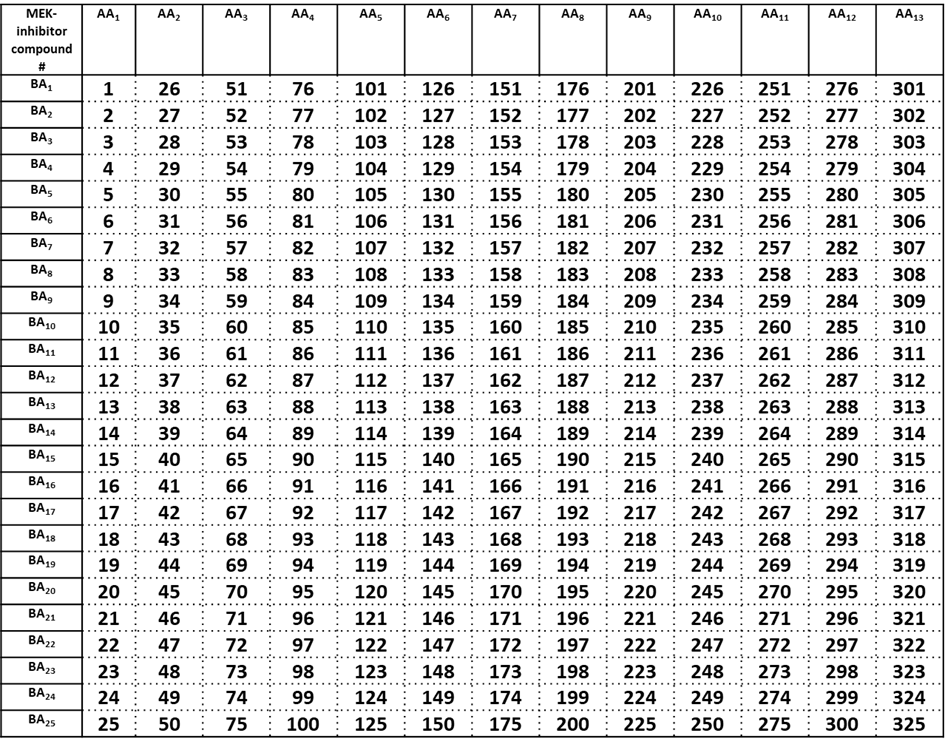


# **Characterization of molecular synthesis on the Nanodroplet Array**

# **Determination of product release of IC 1 (Fmoc-AA_2_-NH_2_)**

The product release of **IC 1** was determined by integrating the absorbance peak in the LC­‑MS measurements. The product release was determined in dependence of the irradiation time. To quantify the product, a concentration range of Fmoc‑Ala‑OH was measured to calibrate the absorbance peak area.

*

**Figure S1.** LC-MS spectra of Fmoc-Ala-NH_2_ released from the surface at various irradiation durations at 365 nm with zoom into the absorption peak at a retention time of 8 – 9 min. The green asterisk shows the main product peak.

**Figure S2.** Calibration curve of Fmoc-Ala-OH to determine the respective Fmoc-Ala-NH_2_ concentration using the integrated area of the product peak in the UV chromatogram of the LC-MS measurement. The data set was fitted linearly with y = (7450,3 ± 15,2)x, where x is the concentration in µM and y the integrated absorption area at 254 nm in %.

# **Purity analysis and quantification of IC 2**

The purity of all compounds synthesized on the Nanodroplet Array was analyzed by measuring LC-MS. The UV chromatogram at 254 nm was extracted and all signals above the threshold were integrated. The main peak integral was divided by the total sum of peak integrals with exception of the injection solvent peak.

**Figure S3.** Extracted UV chromatogram at 254 nm of **IC 2** after synthesis on the solid phase Nanodroplet Array substrate and cleavage by UV irradiation at 365 nm. The integrated area of the peaks above the threshold with exception of the injection solvent peak were used to determine the purity.

$$Purity \left( \% \right)=\frac{Area (Product)}{Area \left( Product \right)+Area (Impurity)}$$

**Table S2.** Calculation of the product purity by dividing the product peak area with the sum of the total peak area of impurities and main product peak area.

| Sample | Product Peak Area | Total Peak Area of Impurities | Sum of Peak Area | Purity (%) |
| --- | --- | --- | --- | --- |
| AA_2_-FIBA (IC 2) | 368253 | 96283,93 | 464537 | 0,792731 |

The amount of **IC 2** was quantified by using a calibration curve for the absorption of Ala-FIBA which was synthesized in flask.

**Figure S4.** Calibration curve of Ala-FIBA synthesized in flask to determine the respective **IC 2** concentration and loading using the integrated area of the product peak in the UV chromatogram of the LC-MS measurement. The data set was fitted linearly with y = (4595,2 ± 53,5)x, where x is the concentration in µM and y the integrated absorption area at 254 nm in %.

# **Purity analysis and conversion rate of IC 3 (AA_2_-FIBA-BA_18_)**

The conversion rate was analyzed with LC-MS similar to the purity determination in 2.2. The main peak integral was divided by the sum of the main product peak integral and starting material peak integral.

**Figure S5.** Extracted UV chromatogram at 254 nm of **IC 3** after synthesis on the solid phase of the Nanodroplet Array substrate and cleavage by UV irradiation at 365 nm. The integrated area of the peaks above the threshold with exception of the injection solvent peak were used to determine the purity.

$$Conversion rate \left( \% \right)=\frac{Pure product area in \%}{Pure product area in \%+Starting material area in \%}*100$$


**Figure S6.** Calibration curve of **IC 3** synthesized in a round bottom flask.

**Table S3.** Calculation of the conversion rate in % to obtain the different **IC 3**  by dividing the pure product area with the sum of the pure product and starting material area. The areas were given in % and the pure product area was calculated by dividing the main area with the sum of all impurity areas.


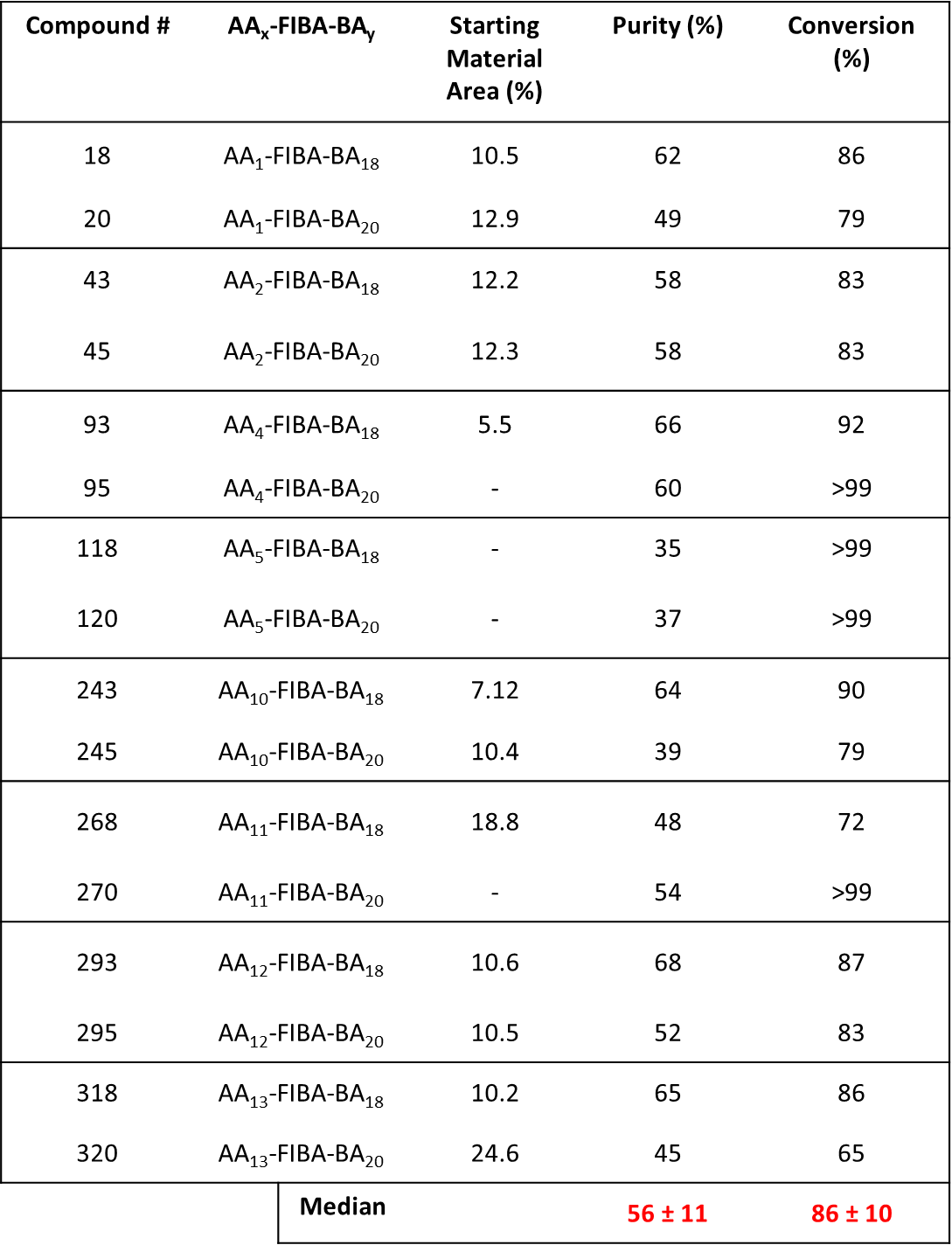


# **Determination of suitable assay concentration**

The assay concentration was determined by measuring the release of **IC 2** and calculating the assay concentration of **IC 3** on 1.4 mm spot size with an assay volume of 1 µL.

**Figure S7.** Measured release of compound **IC 2** as function of the photolinker solution concentration together with the respective achievable assay concentration of **IC 3** based on the average conversion rates for the Suzuki-reaction.

**Figure S8.** Measured release of compound **IC 2** synthesized using 0.1 and 0.3 mM photolinker concentration with varying irradiation times. The corresponding assay concentration for **IC 3** was calculated as well, the release of **IC 2** plateaued after 6 min using 0.1 mM FPL-modification solution and after 18 min using 0.3 mM, respectively.

# **Mass spectrometry**

# **Fragmentation analysis with MS/MS**


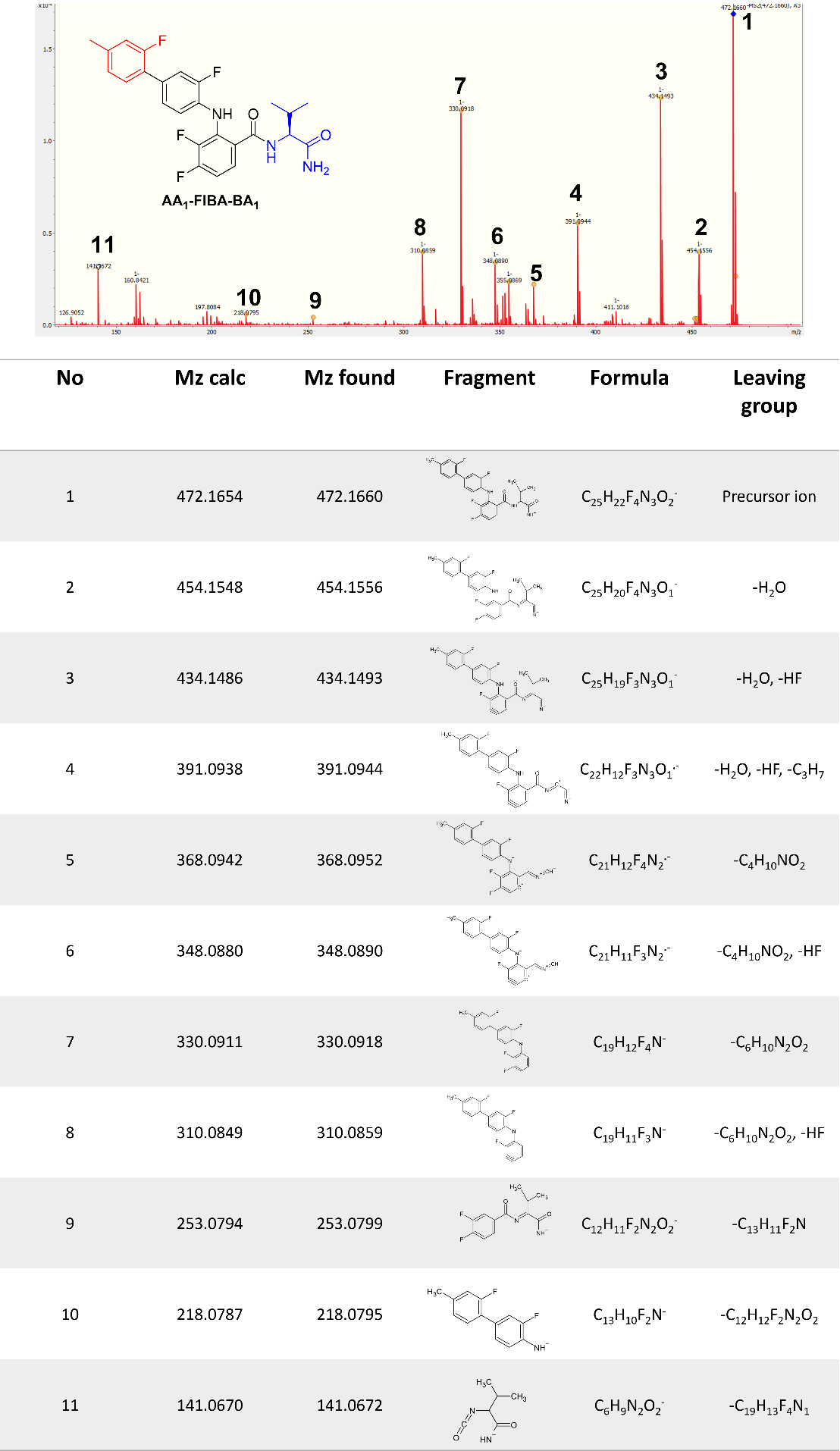


**Figure S9.** MS/MS spectrum obtained from isolation and fragmentation of the [M-H]^-^ ion of a compound **1**, **AA_1_‑FIBA-BA_1_**, (top) with full fragment analysis (bottom).


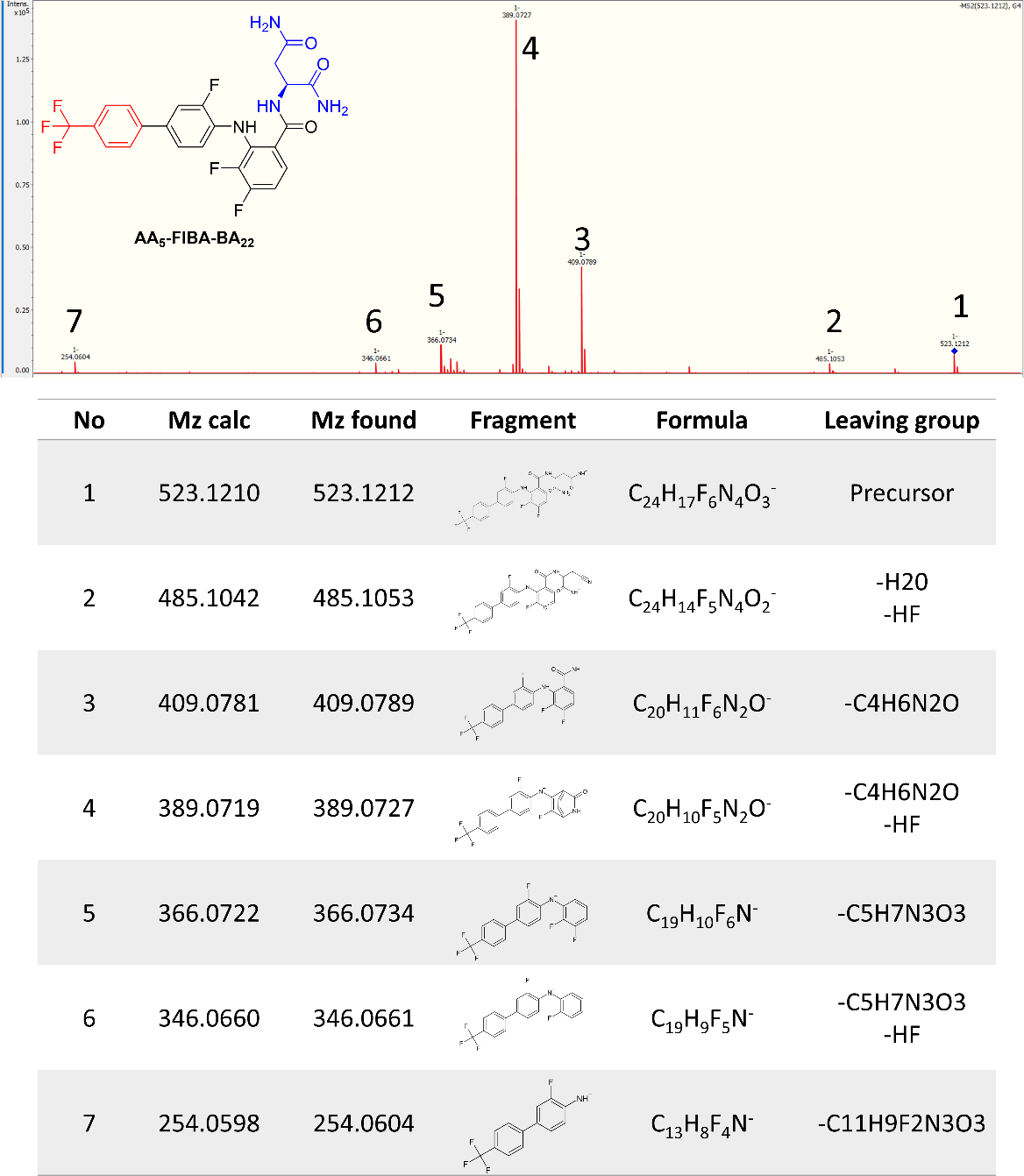


**Figure S10.** MS/MS spectrum obtained from isolation and fragmentation of the [M-H]^-^ ion of a compound **122**, **AA_5_‑FIBA‑BA_22_**, (top) with full fragment analysis (bottom).


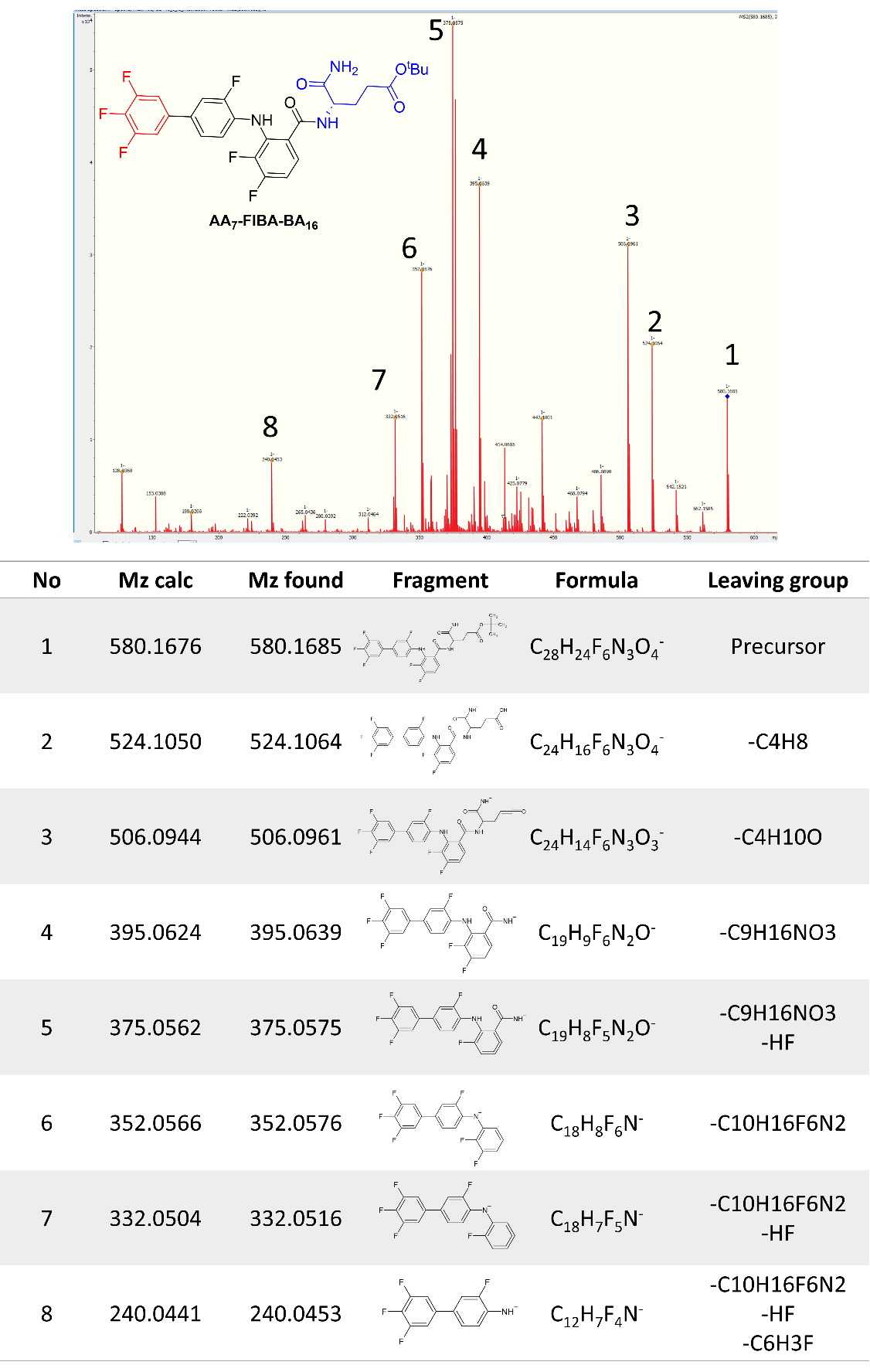


**Figure S11.** MS/MS spectrum obtained from isolation and fragmentation of the [M-H]^-^ ion of a compound **166**, **AA_7_‑FIBA‑BA_16_**, (top) with full fragment analysis (bottom).


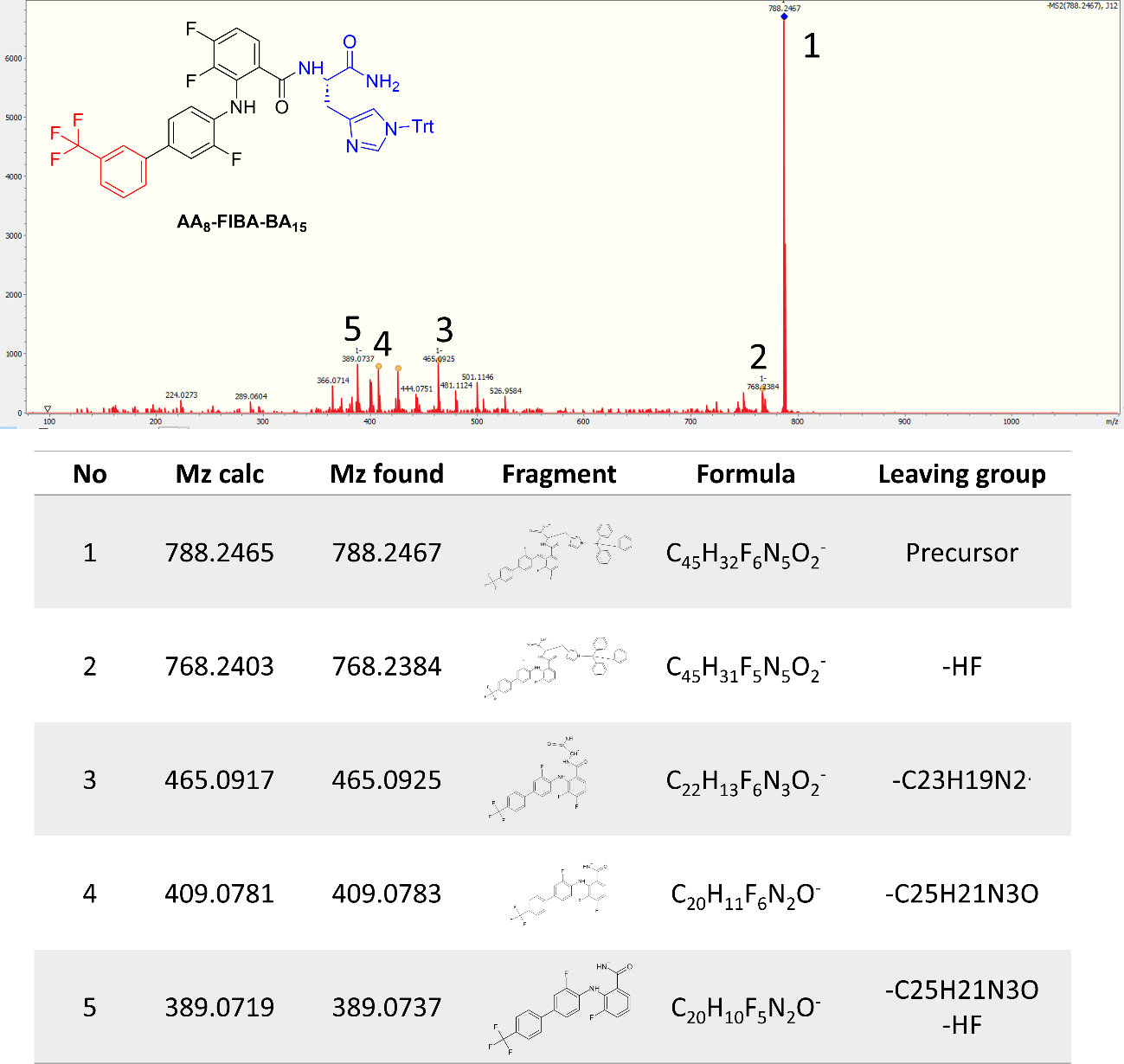


**Figure S12.** MS/MS spectrum obtained from isolation and fragmentation of the [M-H]^-^ ion of a compound **190**, **AA_8_‑FIBA‑BA_15_**, (top) with full fragment analysis (bottom).


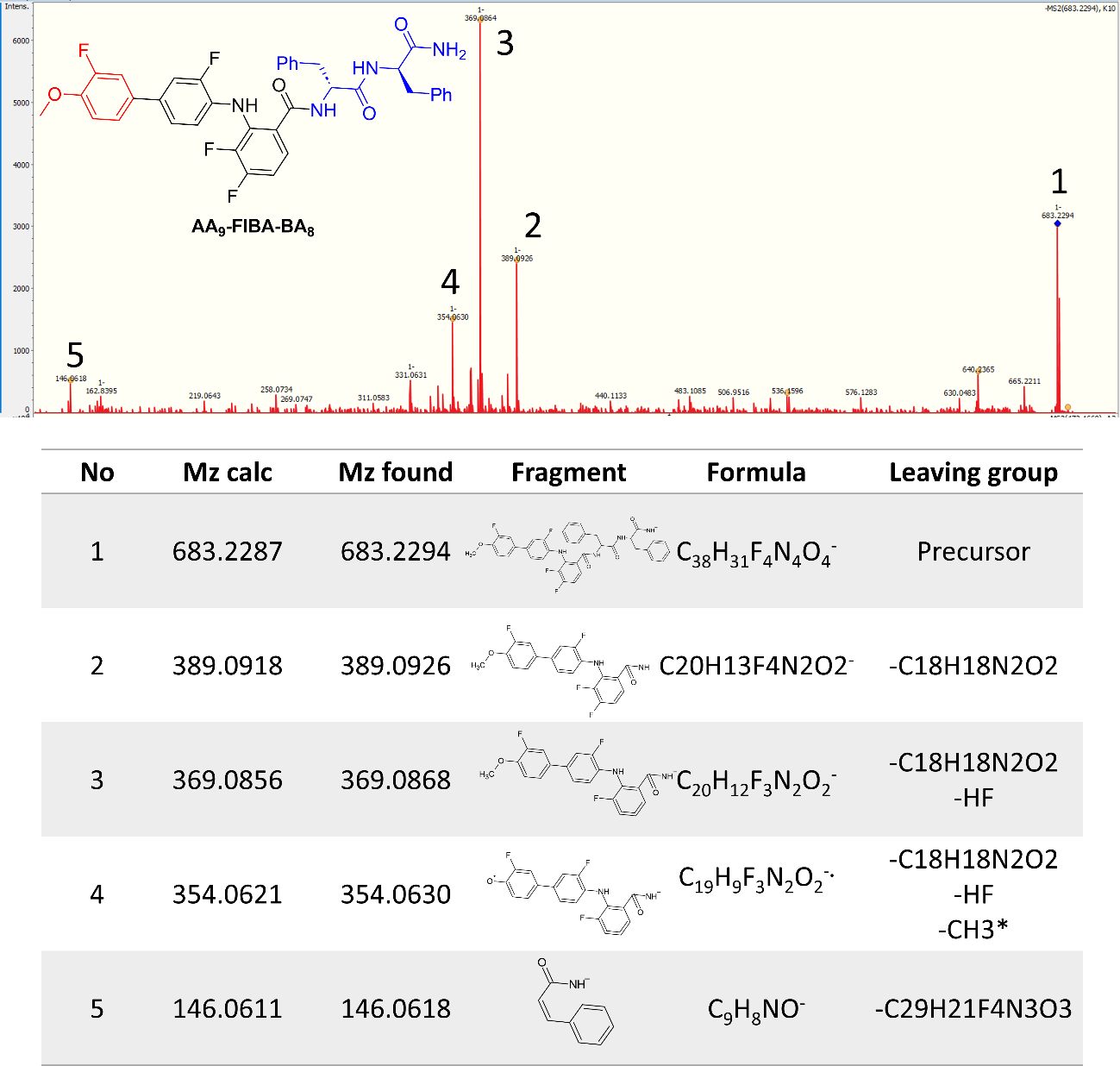


**Figure S13**. MS/MS spectrum obtained from isolation and fragmentation of the [M-H]^-^ ion of a compound **208**, **AA_9_‑FIBA‑BA_8_**, (top) with full fragment analysis (bottom).

# **Results of the on‑chip and target plate analysis**


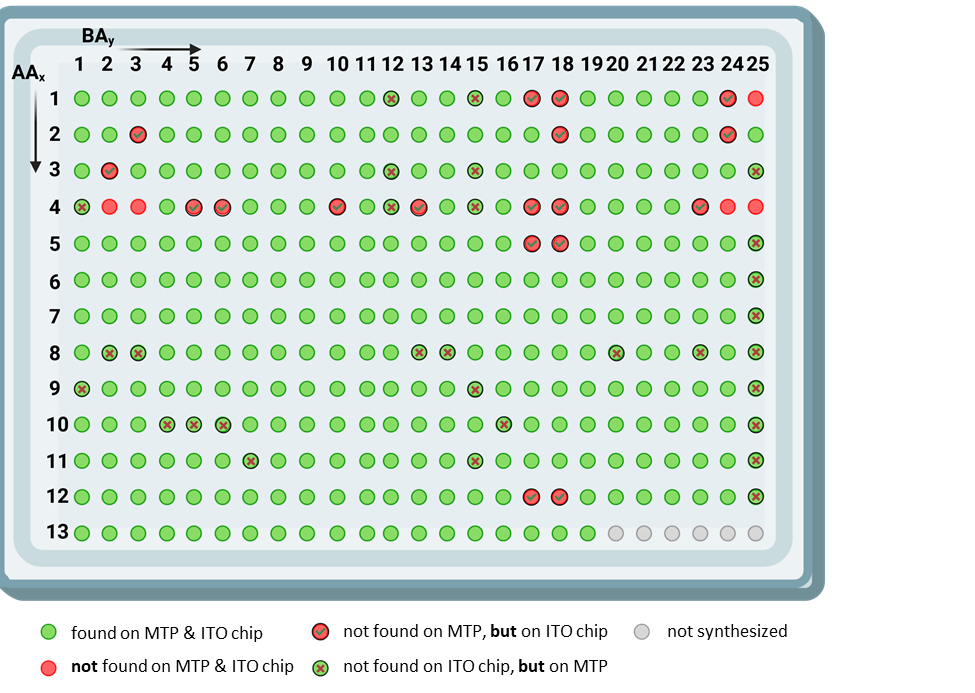


**Figure S14**. Overview of the successful identification of the compound measured either on the MALDI target plate (MTP) or the ITO-modified chip.

# **Biological Screening**

# **Identified Hit Compounds**

**Figure S15.** Structures of the 46 compounds identified in the screening that showed a significant increase in cell death as compared to the untreated cells. The compounds contain either AA_2_ (compounds 26-50) or AA_3_ (compounds 51-75).

# **Library Screening with HT‑29 cells**

A

B

C

D

E

**Figure S16.** Normalized screening results of all 320 synthesized compounds on the 1.4 mm sized Nanodroplet Array substrate. The normalized cell death was calculated using the negative control as zero and positive control (mirdametinib, 10 µM) as 100 % induction of cell death. Error bars show the standard deviation of three replicates. (A) Compounds 1-75 containing AA_1_, AA_2_ and AA_3_. (B) Compounds 76-150 containing AA_4_, AA_5_ and AA_6_. (C) Compounds 151 – 225, containing AA_5_, AA_6_ and AA_7_. (D) Compounds 226 – 300, containing AA_8_ and AA_9_. (E) Complete overview of the screening results.

**Figure S17.** Comparison of the initial screening results and a reproduction of the compound library.

# **Determination of the IC50 in the dose‑response measurements**

**Table S4.** Fit parameters for the dose response measurements.


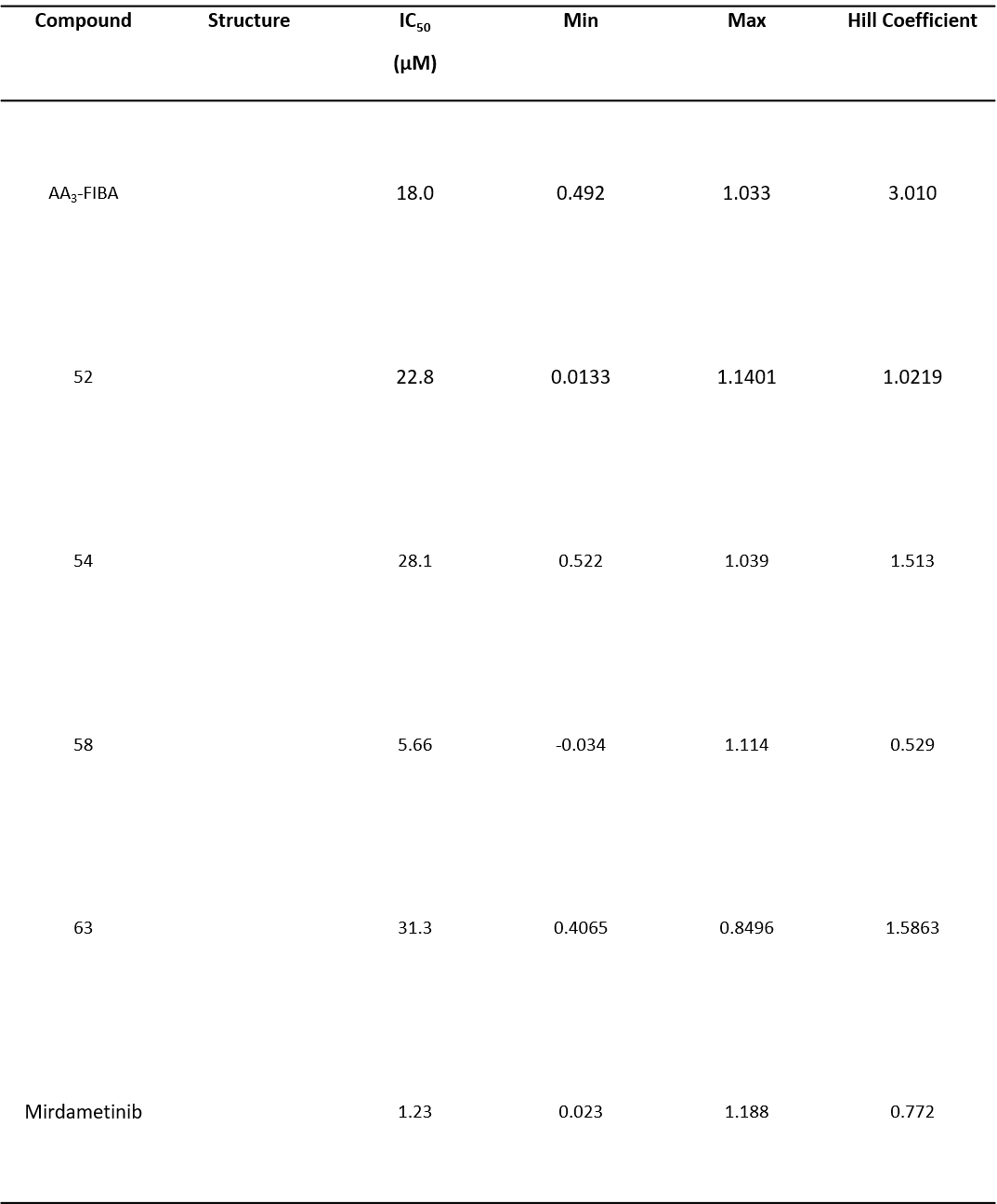


# **Molecular docking simulation**


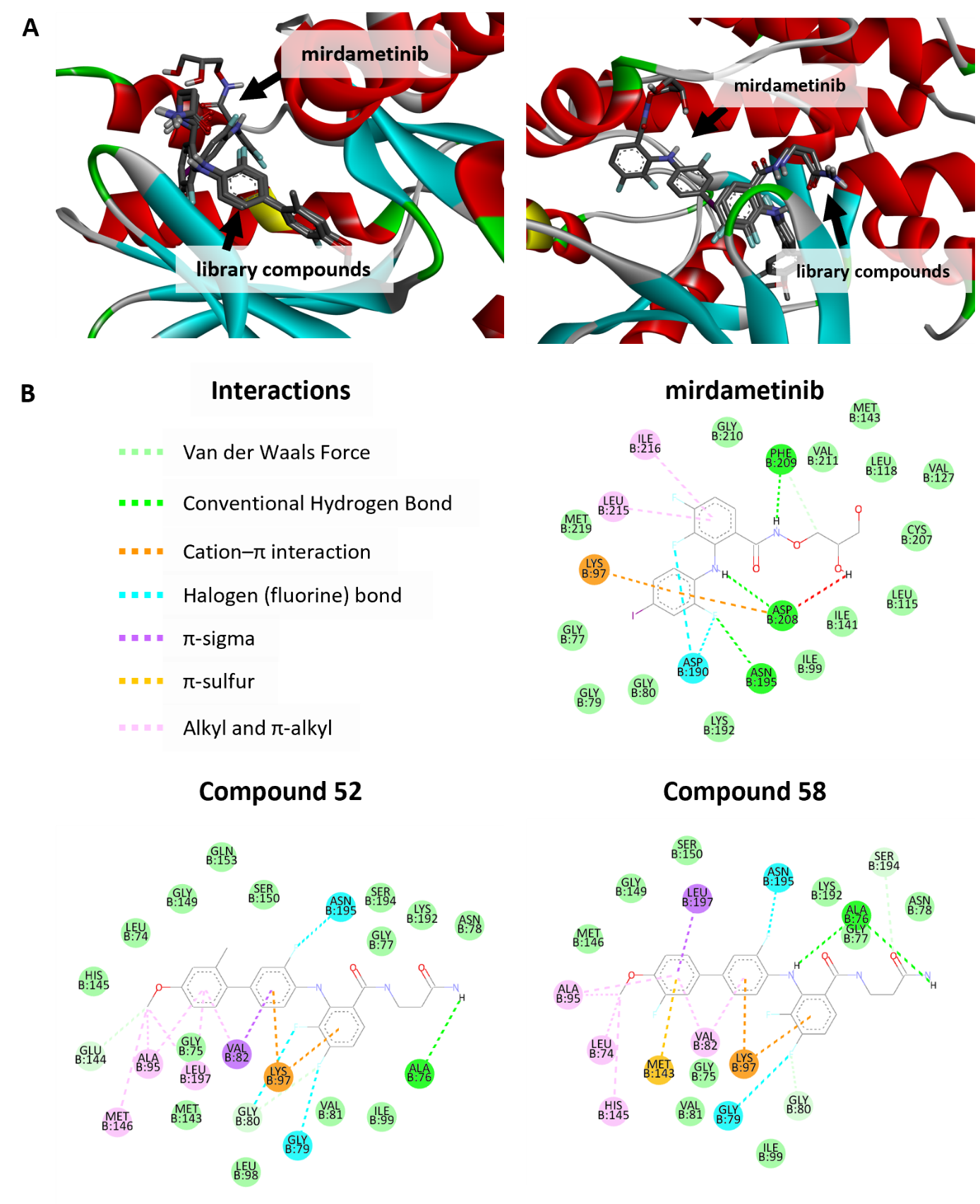


**Figure S18.** Molecular docking simulation of the control drug mirdametinib and two of the library compounds **52** and **58** with MEK1 (PDB: 6v2w). A distinctive change in orientation and additional favorable interactions for the modified compounds can be observed as compared to the FDA approved drug. Simulations were performed with <https://www.swissdock.ch/>. **(A)** Visualization of the orientation of the docked molecules inside of the binding pocket of MEK1. **(B)** 2D interaction map of mirdametinib, compound 52 and 58 with the amino acid residues inside of the protein. The different types of the non‑covalent interactions present in the three simulations are colored individually. The docking and 2D interaction was visualized using Discovery Studio Visualizer.

# **Exemplary LC-MS spectra of library compounds synthesized on the Nanodroplet Array with 2.8 mm diameter spot sizes.**

*

*

*

*

A

B

C

D

**Figure S19.** Exemplary extracted UV-chromatogram from compounds synthesized on the 2.8 mm sized Nanodroplet Array substrate. (A) Library compound **296**, **AA_12_-FIBA-BA_20_**, (B) compound **320**, **AA_13_-FIBA-BA_20_**, (C) compound **243**, **AA_10_-FIBA-BA_18_**, (D) compound **20**, **AA_1_-FIBA-BA_20_**. The main product peak is marked with a green asterisk in each chromatogram.

# **Exemplary mass spectra of library compounds synthesized on pattern with 900 µm diameter**


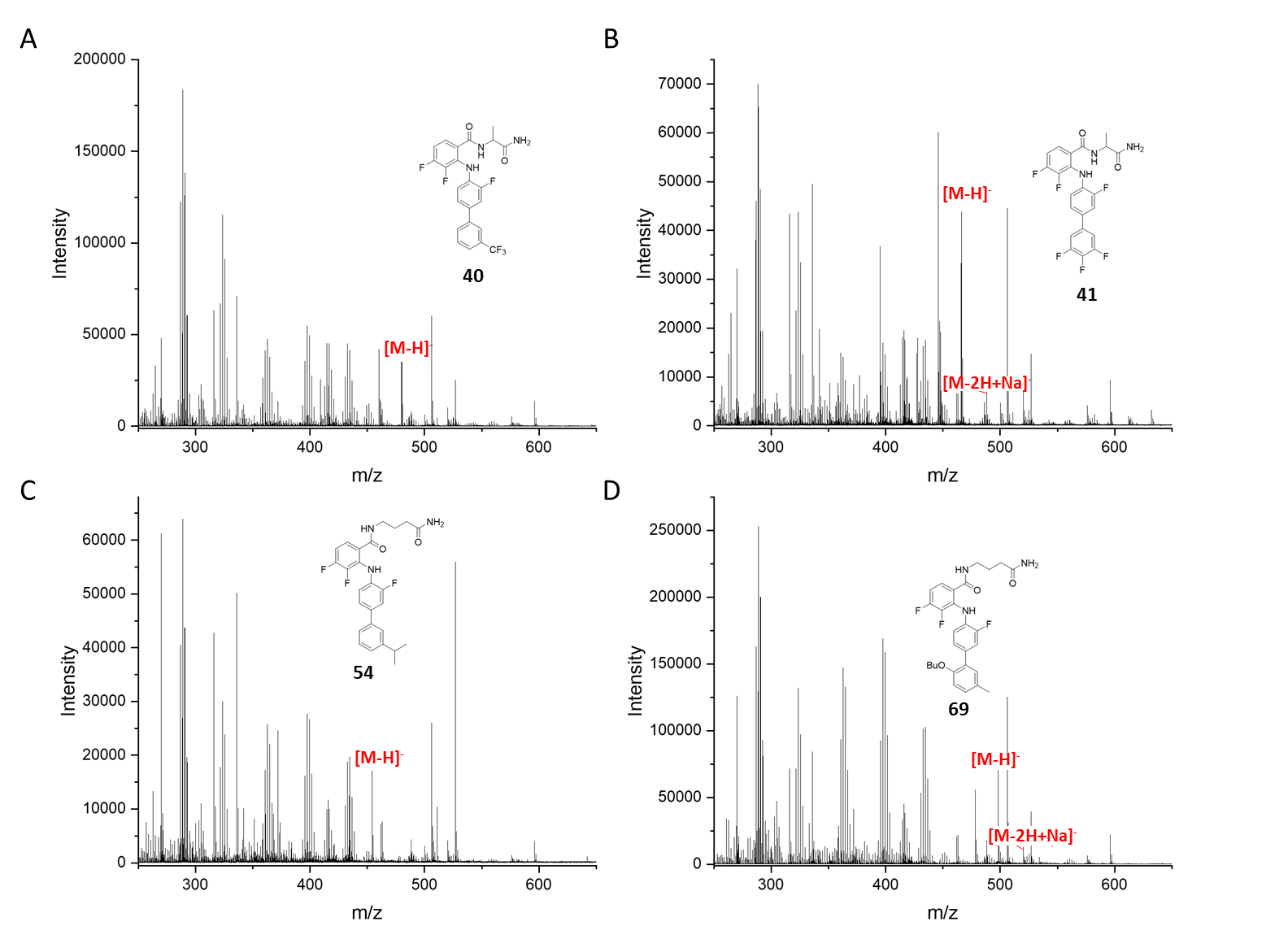


**Figure S20.** Exemplary mass spectra of compounds synthesized on the 1.4 mm sized Nanodroplet Array substrate. (A) Library compound **40**, **AA_2_-FIBA-BA_15_**, (B) compound **41**, **AA_2_-FIBA-BA_16_**, (C) compound **54**, **AA_3_-FIBA-BA_4_**, (D) compound **69**, **AA_3_-FIBA-BA_19_**. The compounds were measured in negative mode and the identified ion is marked in each spectrum.


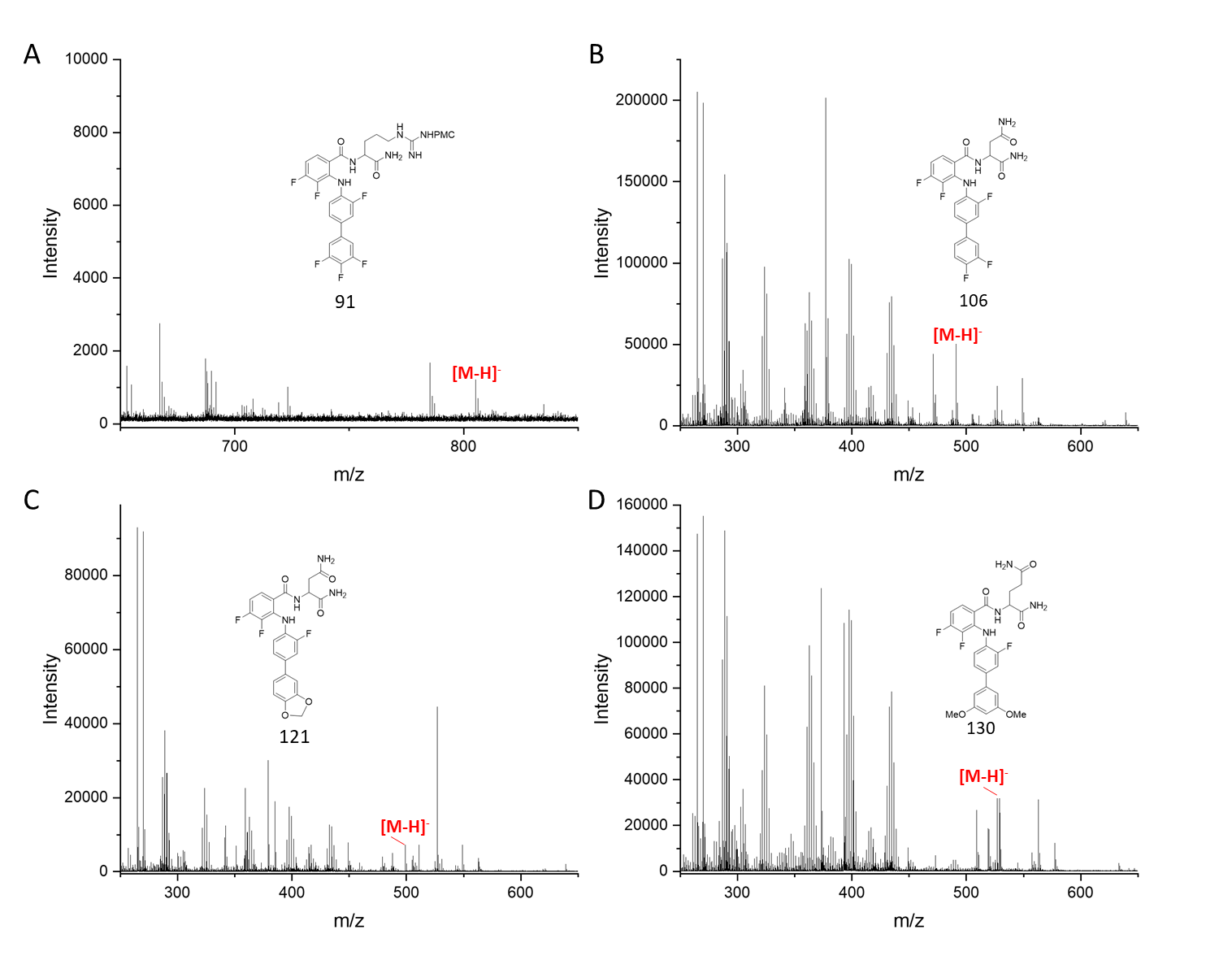


**Figure S21.** Exemplary mass spectra of compounds synthesized on the 1.4 mm sized Nanodroplet Array substrate. (A) Library compound **91**, **AA_2_-FIBA-BA_15_**, (B) compound **106**, **AA_2_-FIBA-BA_16_**, (C) compound **121**, **AA_3_-FIBA-BA_4_**, (D) compound **130**, **AA_3_-FIBA-BA_19_**. The compounds were measured in negative mode and the identified ion is marked in each spectrum.


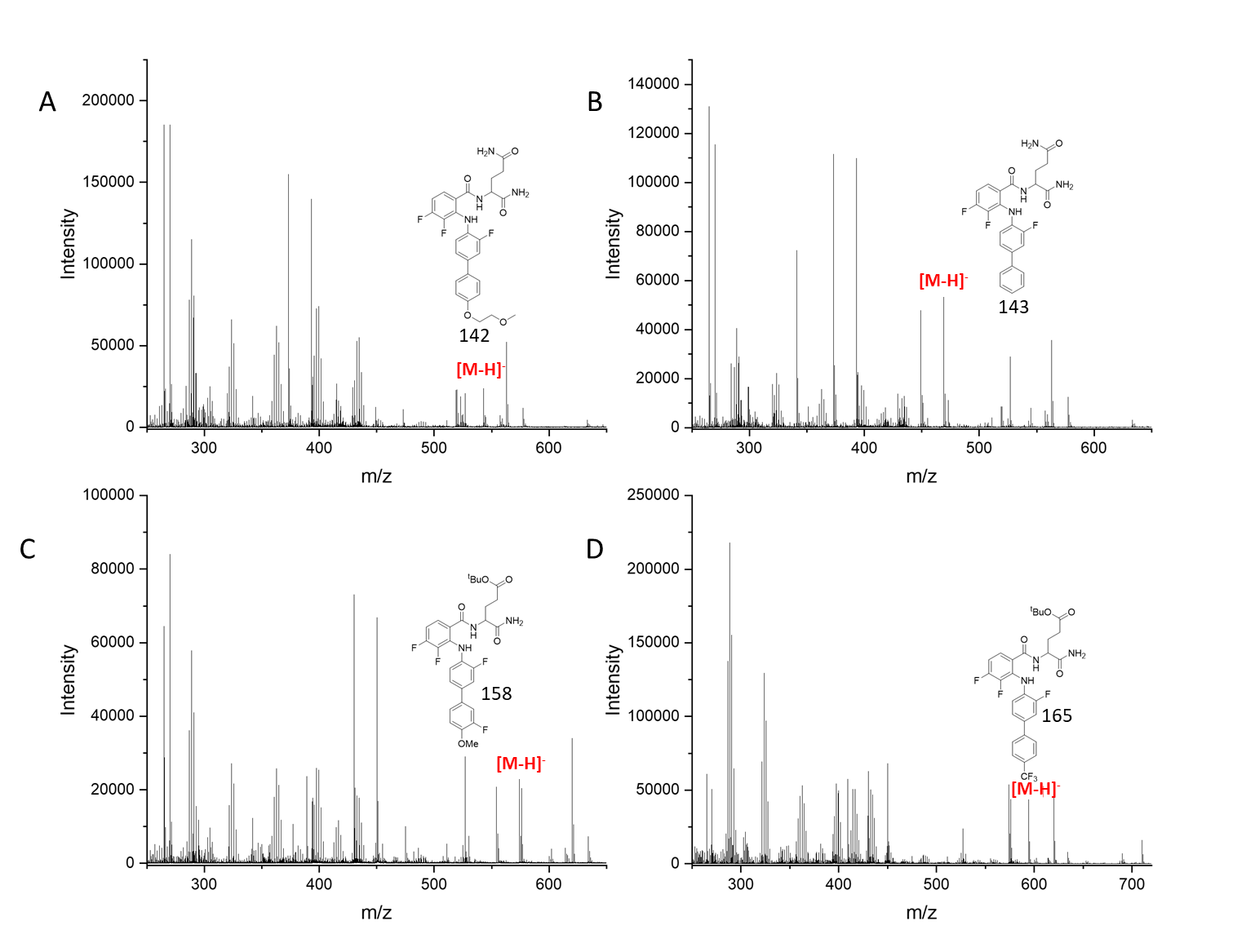


**Figure S22.** Exemplary mass spectra of compounds synthesized on the 1.4 mm sized Nanodroplet Array substrate. (A) Library compound **142**, **AA_2_-FIBA-BA_15_**, (B) compound **143**, **AA_2_-FIBA-BA_16_**, (C) compound **158**, **AA_3_-FIBA-BA_4_**, (D) compound **165**, **AA_3_-FIBA-BA_19_**. The compounds were measured in negative mode and the identified ion is marked in each spectrum.


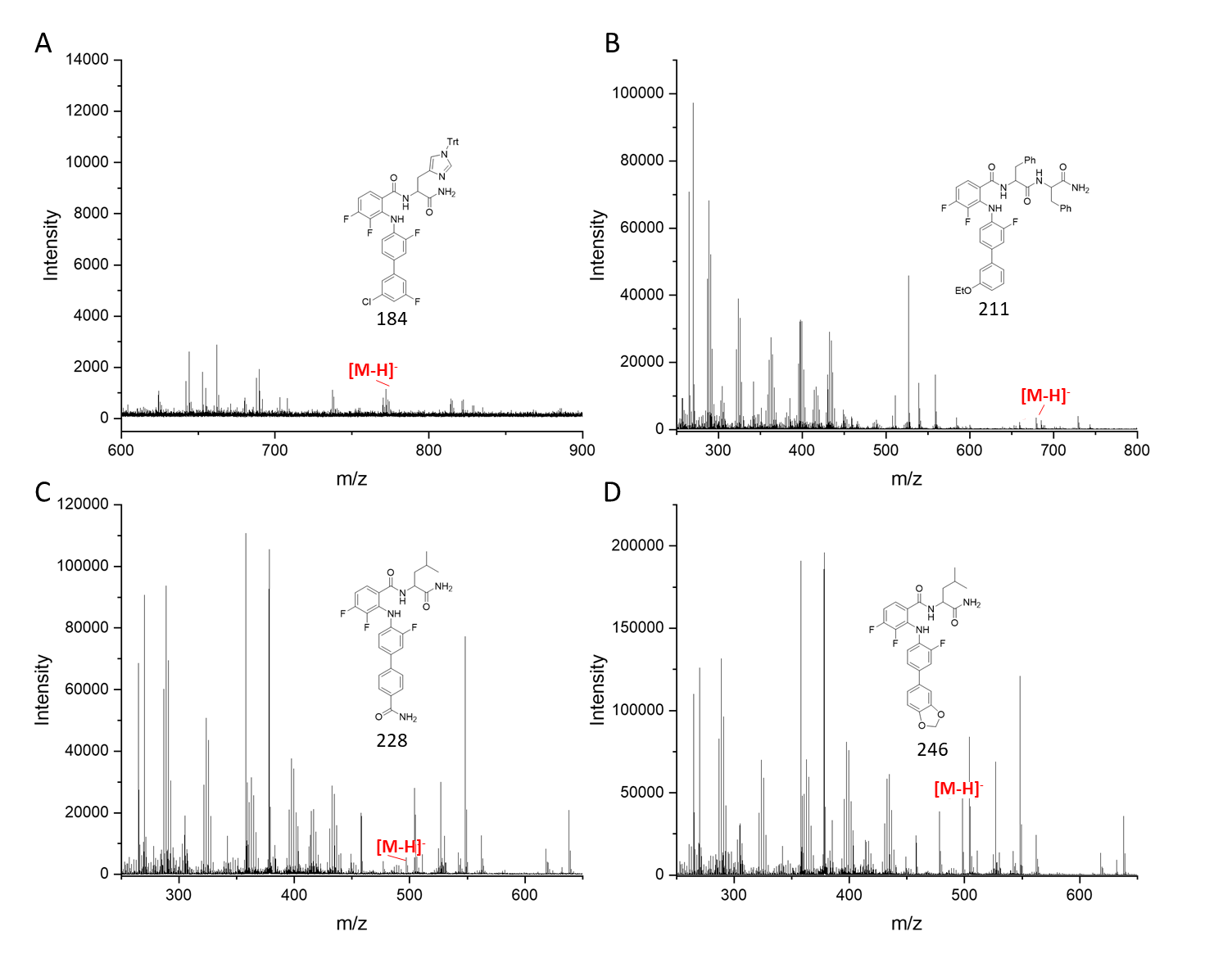


**Figure S23.** Exemplary mass spectra of compounds synthesized on the 1.4 mm sized Nanodroplet Array substrate. (A) Library compound **184**, **AA_2_-FIBA-BA_15_**, (B) compound **211**, **AA_2_-FIBA-BA_16_**, (C) compound **228**, **AA_3_-FIBA-BA_4_**, (D) compound **246**, **AA_3_-FIBA-BA_19_**. The compounds were measured in negative mode and the identified ion is marked in each spectrum.


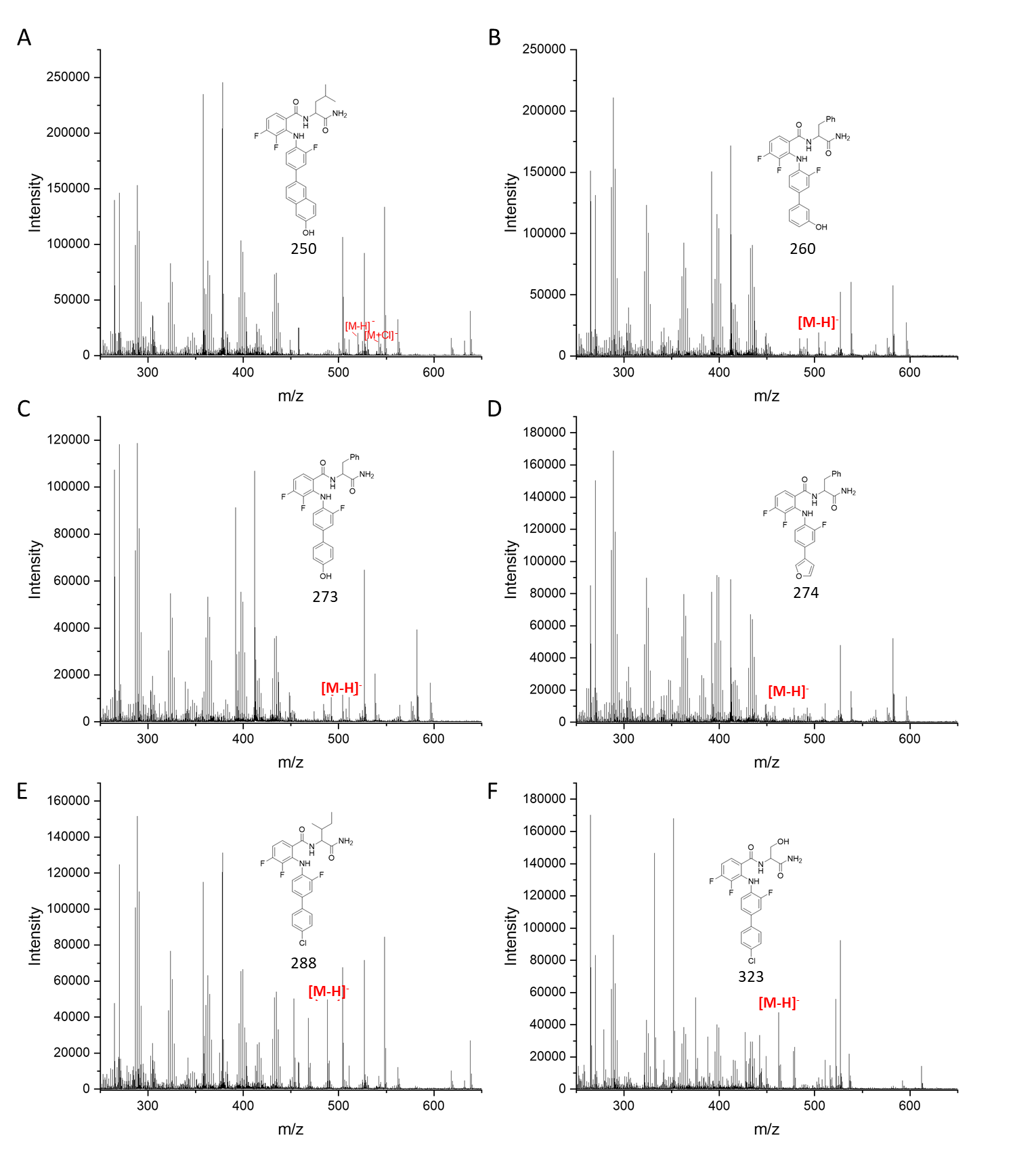


**Figure S24.** Exemplary mass spectra of compounds synthesized on the 1.4 mm sized Nanodroplet Array substrate. (A) Library compound **250**, **AA_2_-FIBA-BA_15_**, (B) compound **260**, **AA_2_-FIBA-BA_16_**, (C) compound **273**, **AA_3_-FIBA-BA_4_**, (D) compound **274**, **AA_3_-FIBA-BA_19_**, (E) compound 288, (F) compound 323. The compounds were measured in negative mode and the identified ion is marked in each spectrum.

# **Analysis of individually synthesized compounds in flask**

# **(S)-N-(1-amino-1-oxopropan-2-yl)-3,4-difluoro-2-((2-fluoro-4-iodophenyl)amino) benzamide (IC 2)**


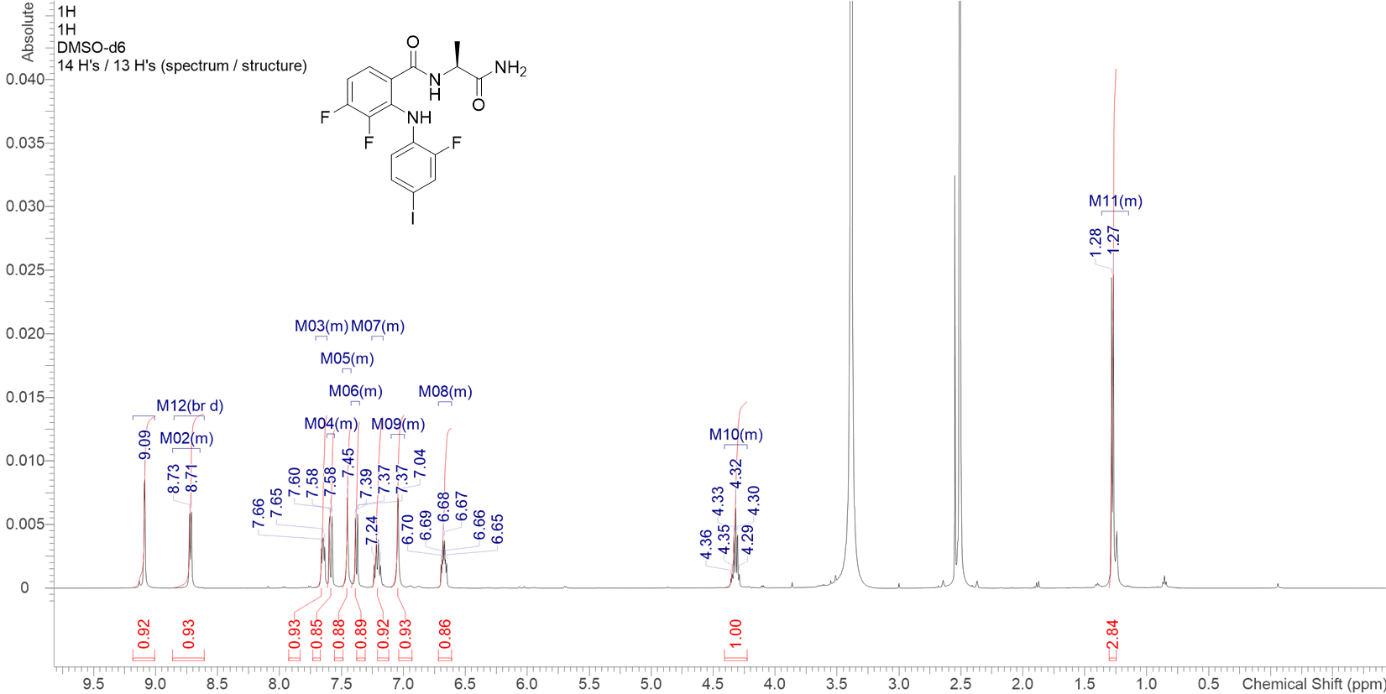


**Figure S25**. ^1^H-NMR spectra of **IC 2**.


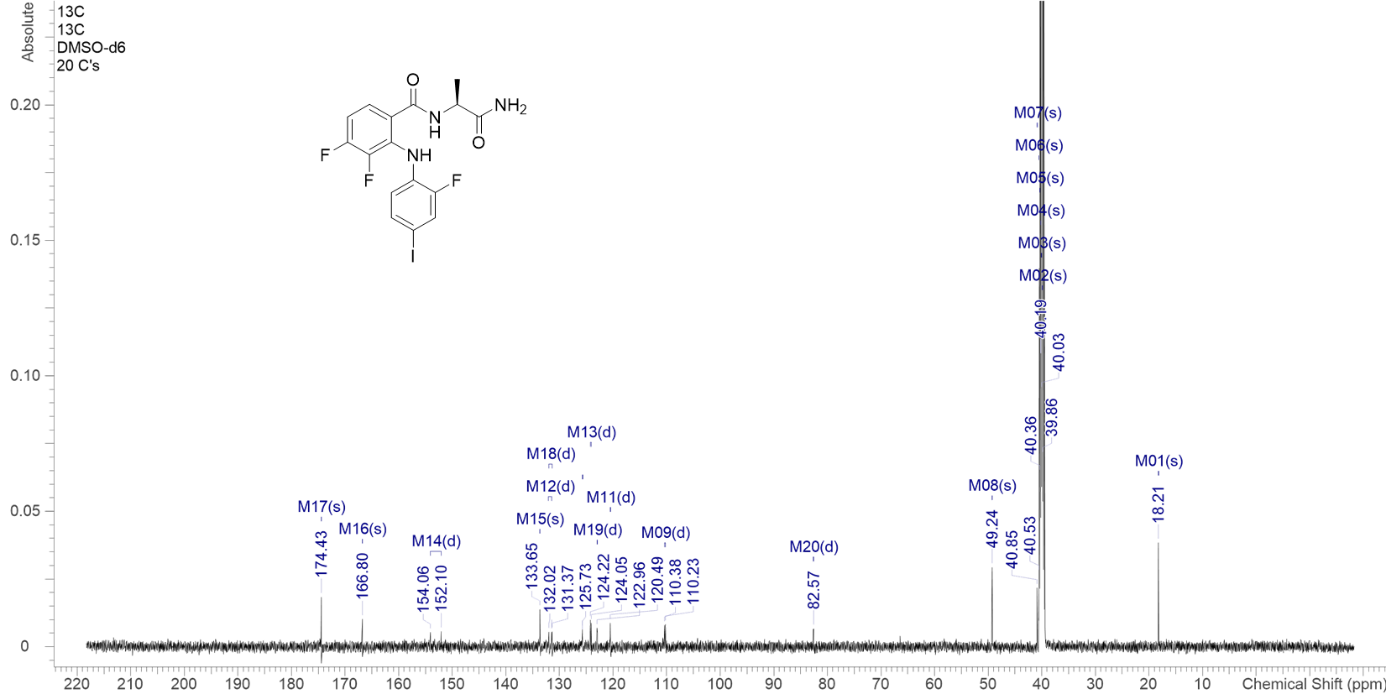


**Figure S26**. ^13^C-NMR spectra of **IC 2**.

[M+H]^+^

*

[M+Na]^+^

**Figure S27.** Mass spectrum and UV spectrum from LC-MS measurement of **IC 2**.

- 1. **(S)-N-(1-amino-1-oxopropan-2-yl)-3,4-difluoro-2-((3-fluoro-[1,1'-biphenyl]-4-yl)amino) benzamide (43, IC 3)**


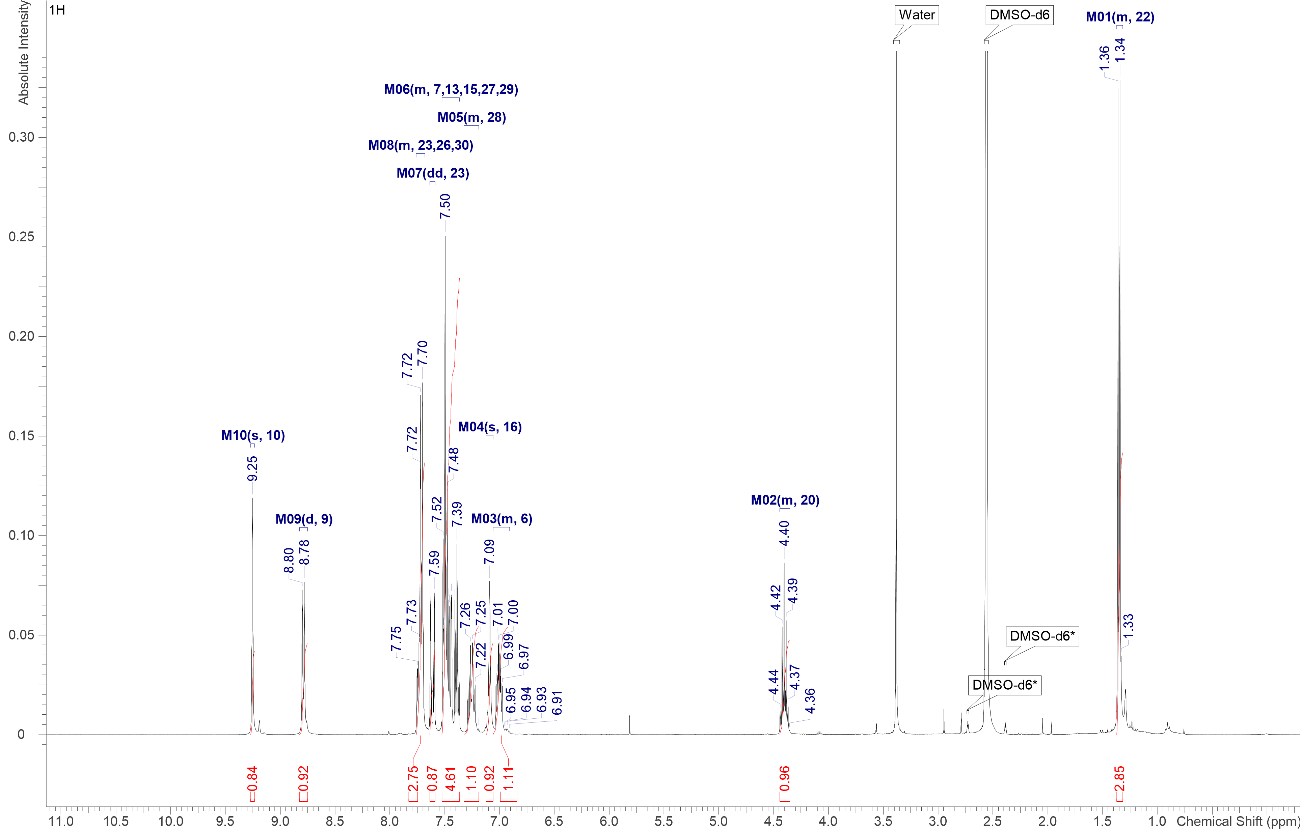


**Figure S28**. ^1^H-NMR spectra of **IC 3**.


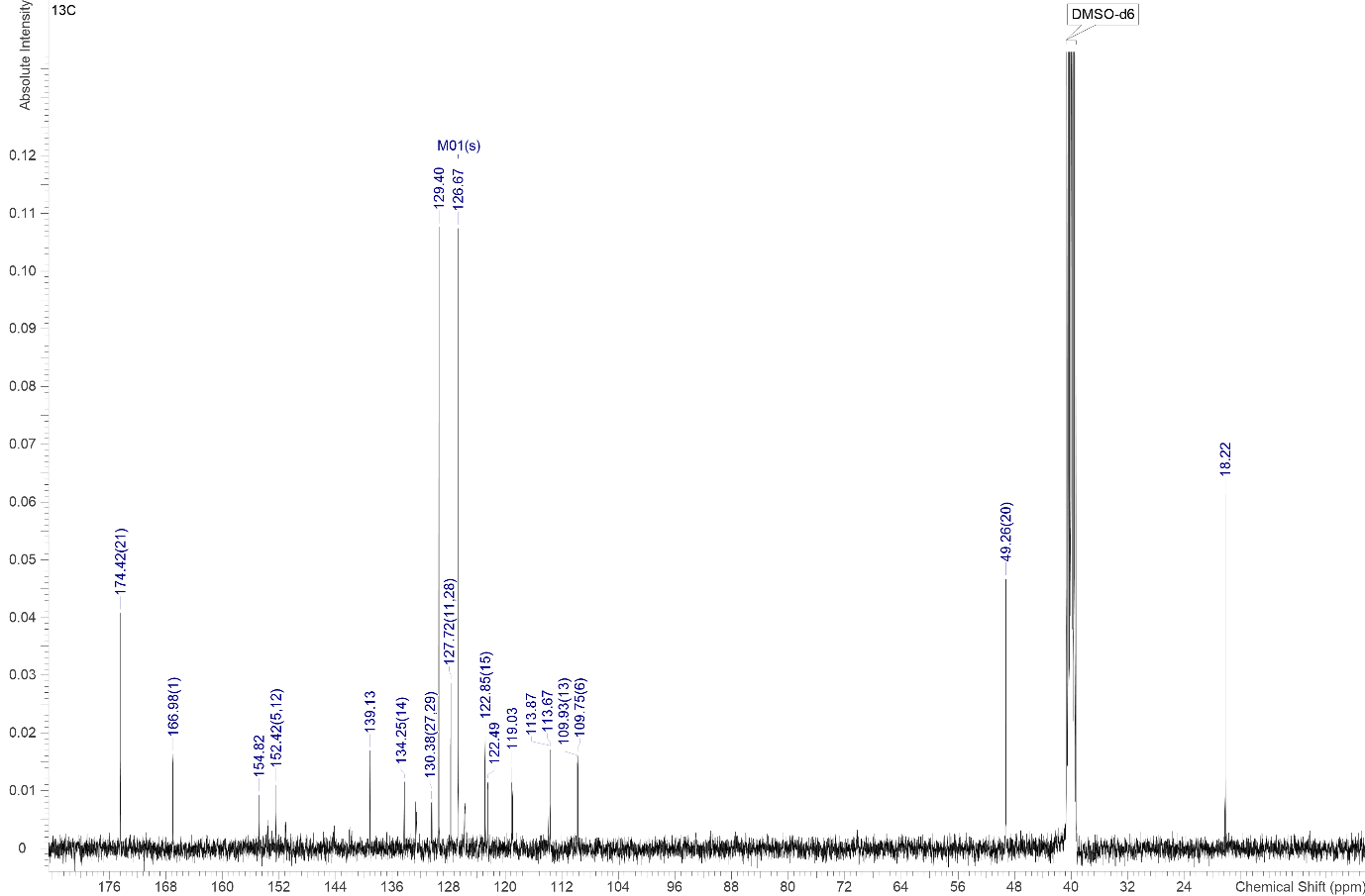


**Figure S29.** ^13^C-NMR spectra of **IC 3**.

[M+H]^+^

*

[M+Na]^+^

**Figure S30.** Mass spectrum and UV spectrum from LC-MS measurement of **IC 3**.

# **N-(3-amino-3-oxopropyl)-3,4-difluoro-2-((2-fluoro-4-iodophenyl)amino)benzamide (AA_3_-FIBA)**


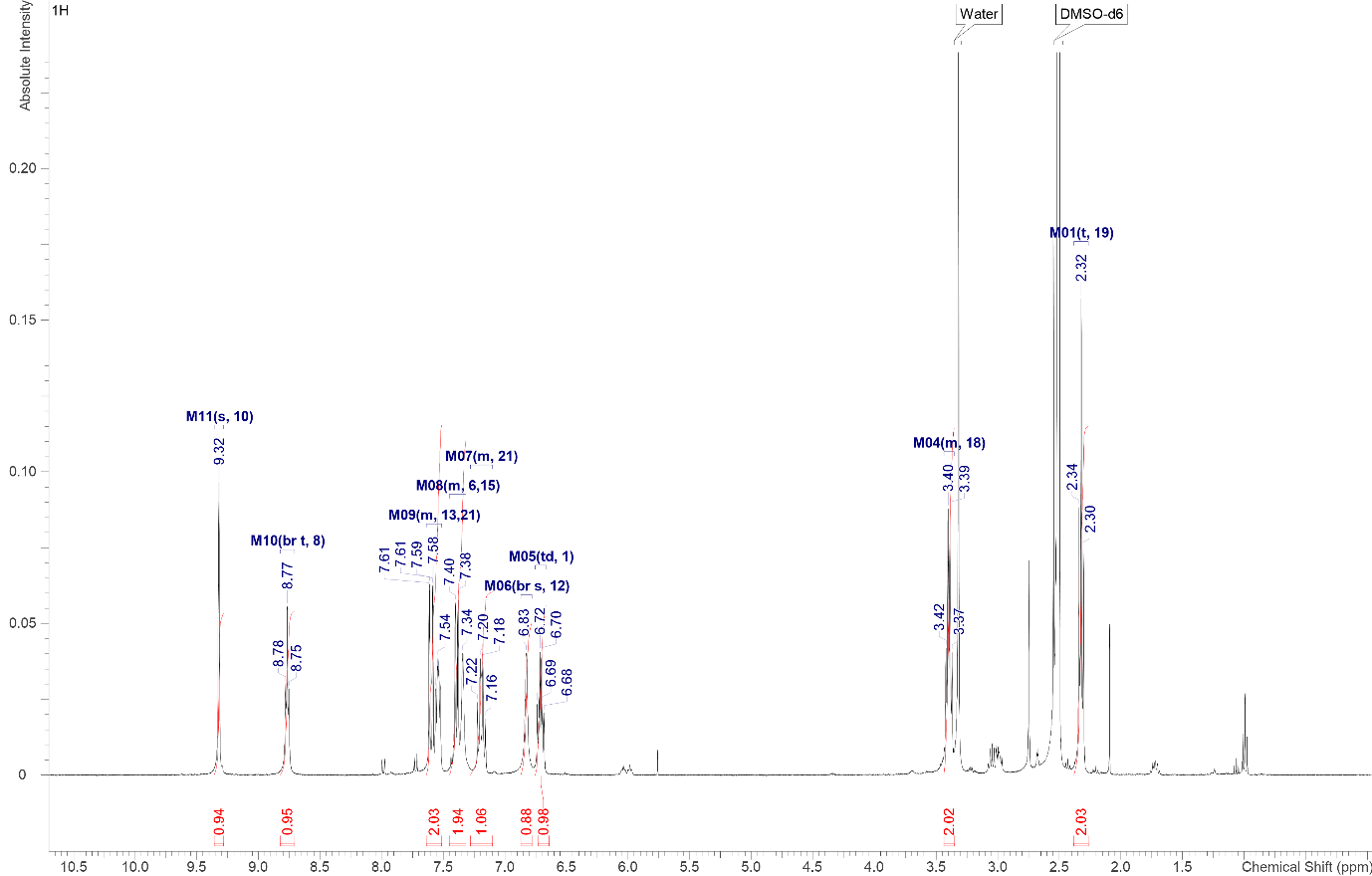


**Figure S31.** ^1^H-NMR spectra of N-(3-amino-3-oxopropyl)-3,4-difluoro-2-((2-fluoro-4-iodophenyl)amino)benzamide (AA_3_-FIBA).

**
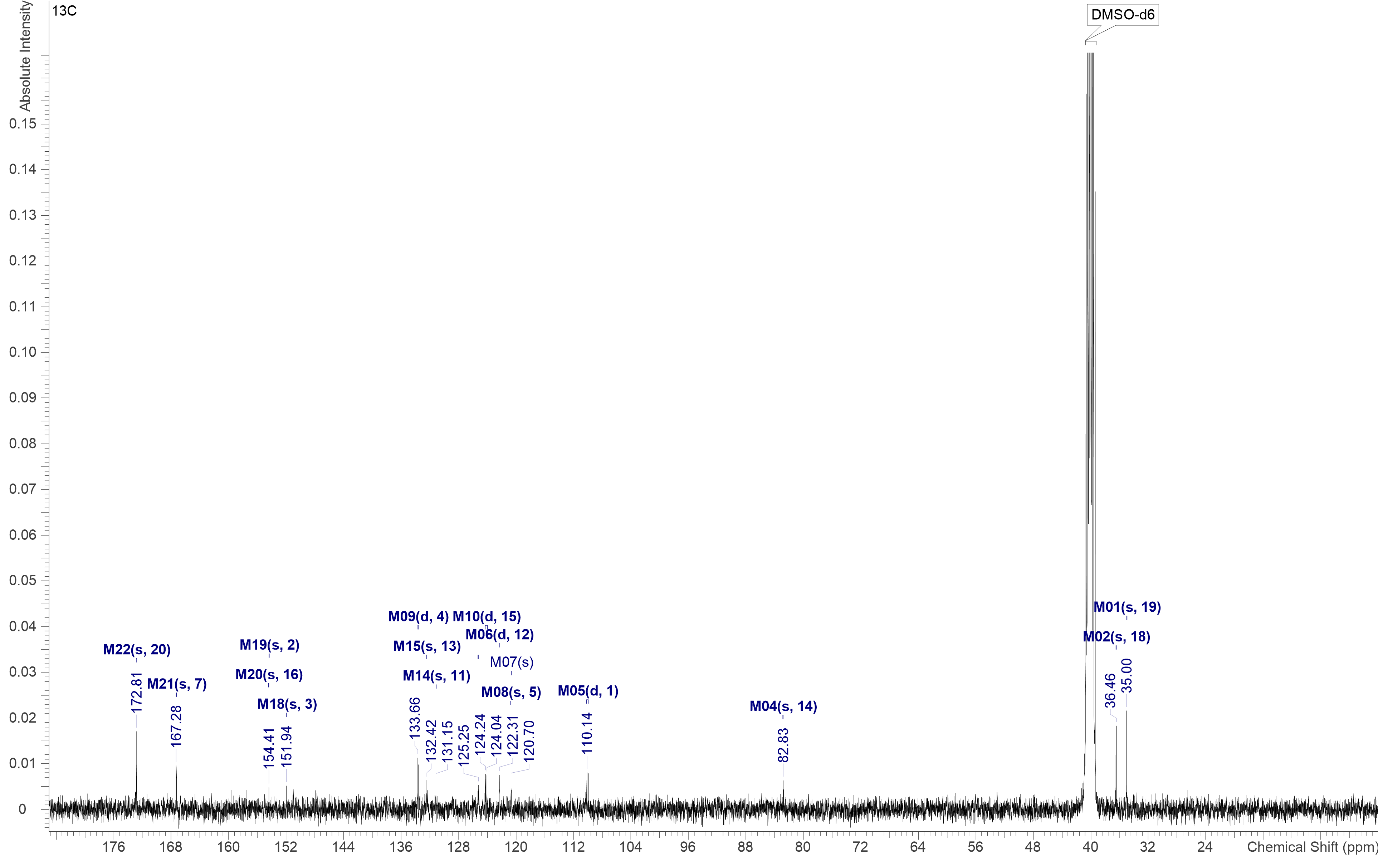
**

**Figure S32.** ^13^C-NMR spectra of N-(3-amino-3-oxopropyl)-3,4-difluoro-2-((2-fluoro-4-iodophenyl)amino)benzamide (AA_3_-FIBA).

[M+H]^+^

*

**Figure S33.** Mass spectrum and UV spectrum from LC-MS measurement of N-(3-amino-3-oxopropyl)-3,4-difluoro-2-((2-fluoro-4-iodophenyl)amino)benzamide (AA_3_-FIBA).

# **N-(3-amino-3-oxopropyl)-3,4-difluoro-2-((3-fluoro-4'-methoxy-2'-methyl-[1,1'-biphenyl]-4‑yl)amino)benzamide (Compound 52)**


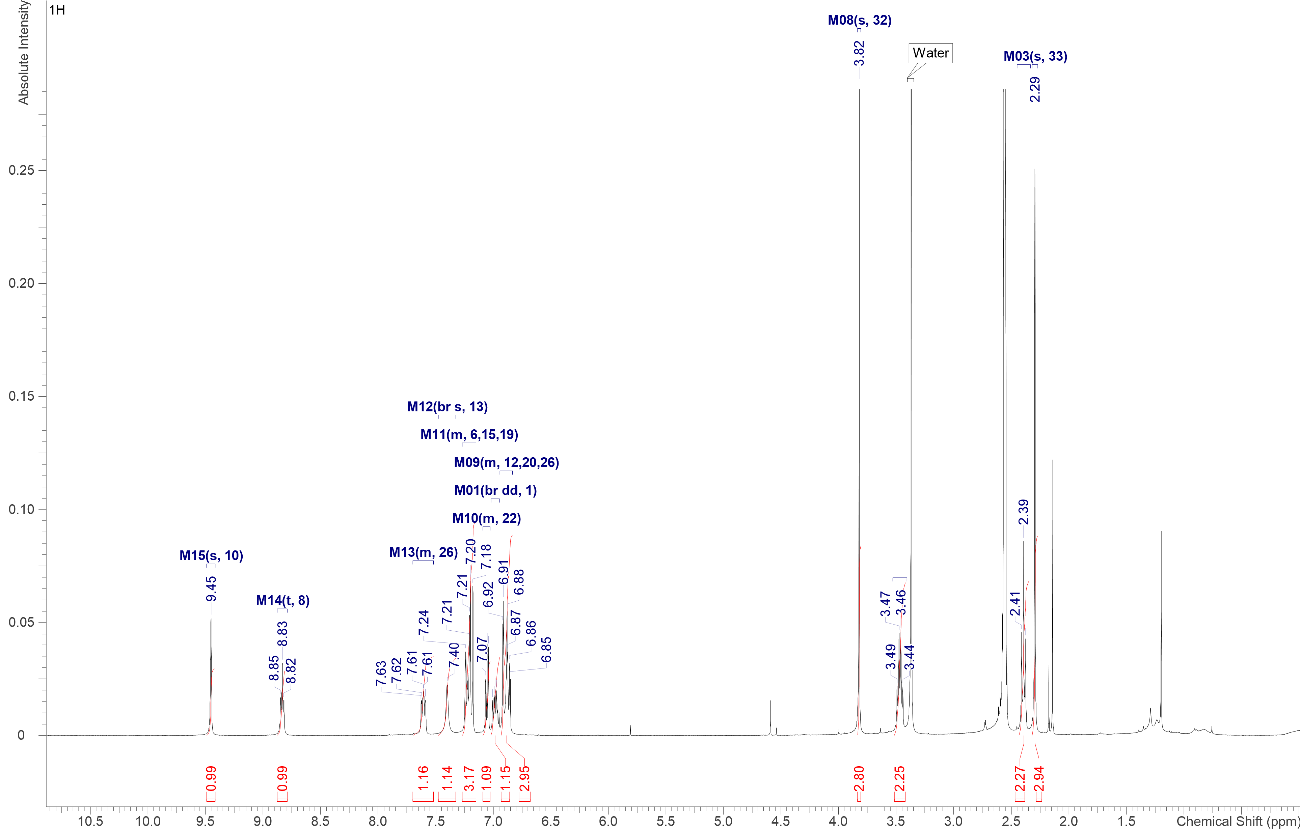


**Figure S34.** ^1^H-NMR spectra of compound **52**.


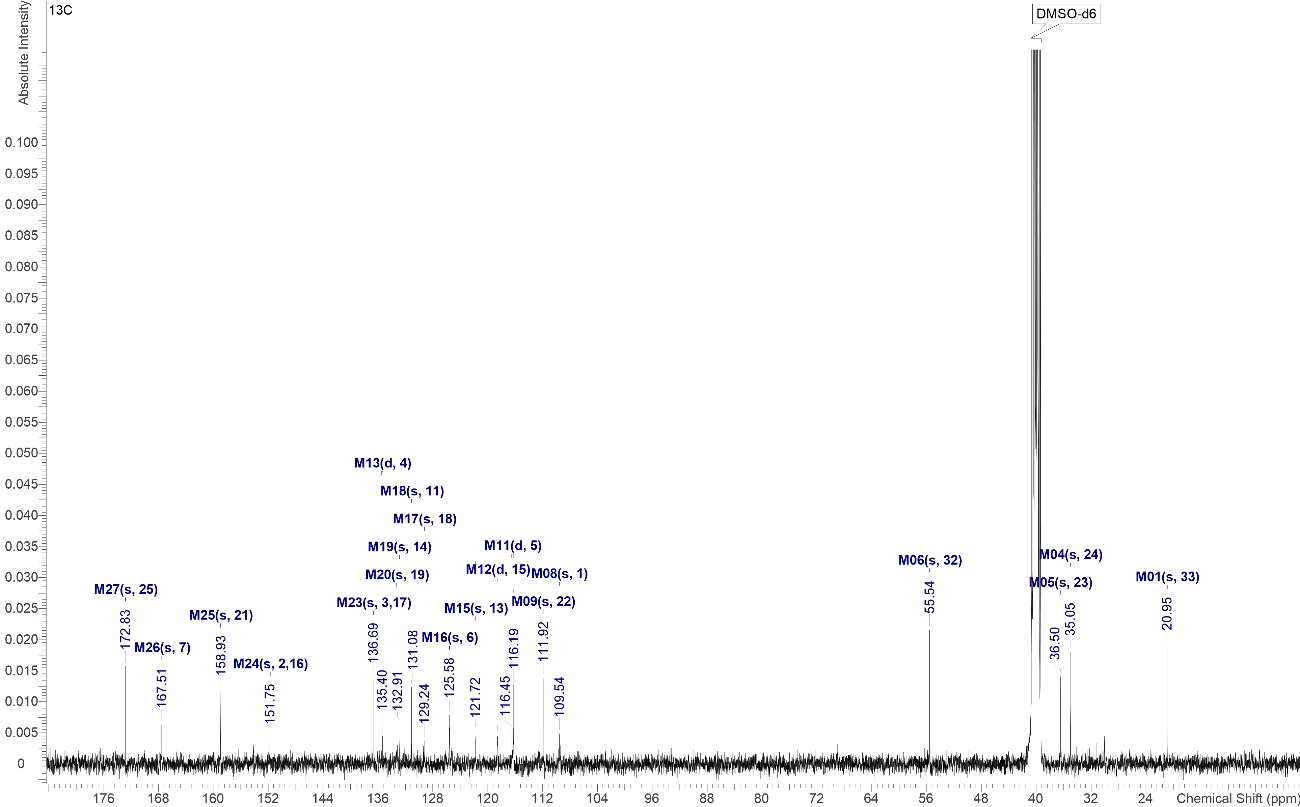


**Figure S35**. ^13^C-NMR spectra of compound **52**.

[M+H]^+^

*

**Figure S36.** Mass spectrum and UV spectrum from LC-MS measurement of compound **52**.

- 1. **N-(3-amino-3-oxopropyl)-3,4-difluoro-2-((3-fluoro-3'-isopropyl-[1,1'-biphenyl]-4-yl)amino)benzamide (Compound 54)**


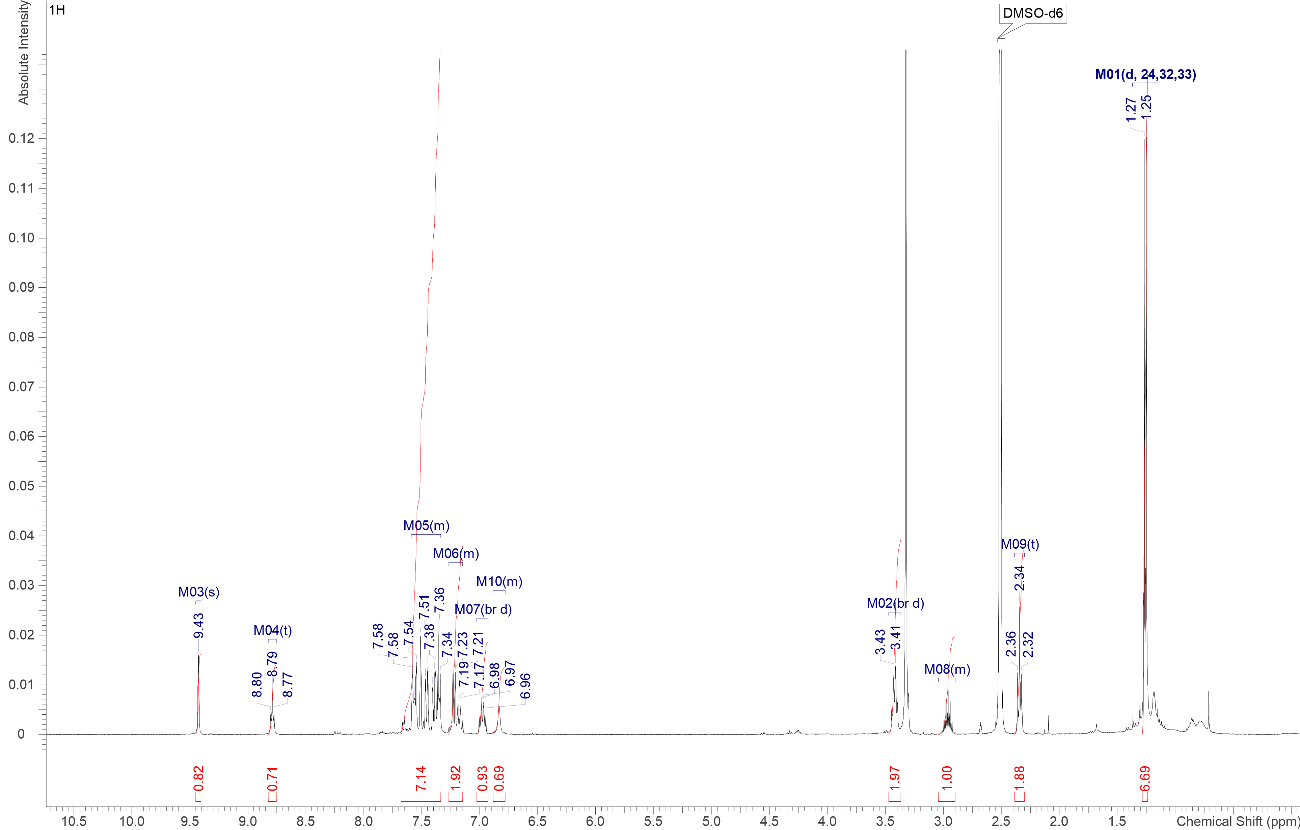


**Figure S37.** ^1^H-NMR spectra of compound **54**.


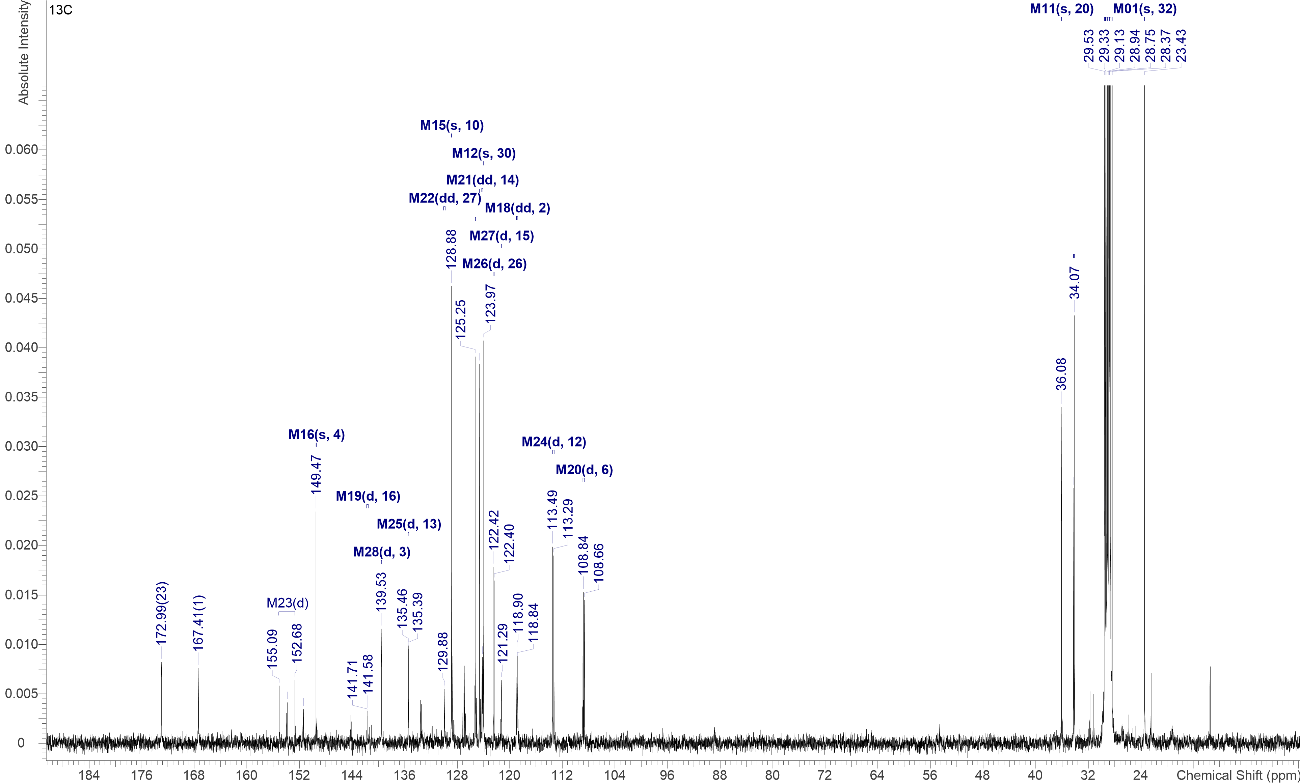


**Figure S38**. ^13^C-NMR spectra of compound **54**.

[M+H]^+^

*

**Figure S39.** Mass spectrum and UV spectrum from LC-MS measurement of compound **54**.

# **N-(3-amino-3-oxopropyl)-2-((3,3'-difluoro-4'-methoxy-[1,1'-biphenyl]-4-yl)amino)-3,4-difluorobenzamide (Compound 58)**


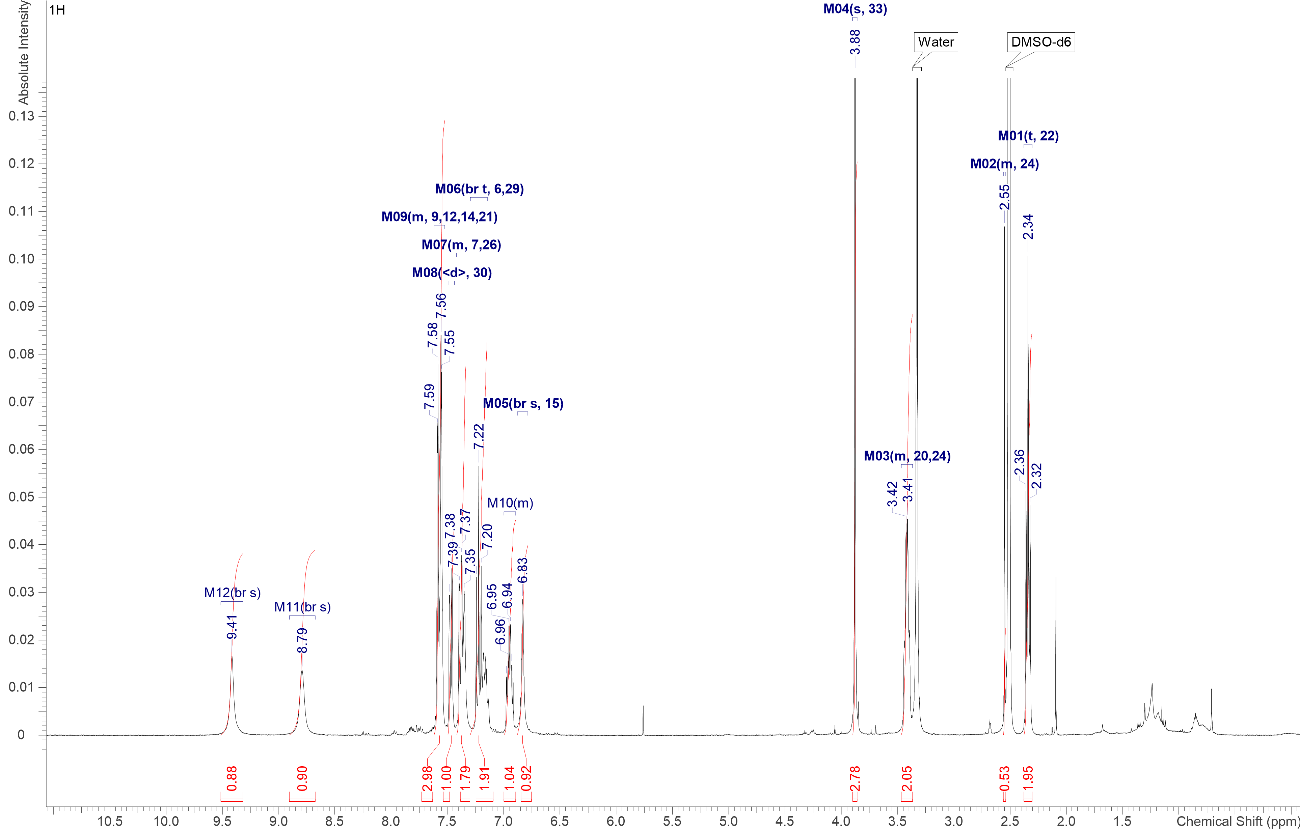


**Figure S40.** ^1^H-NMR spectra of compound **58**.


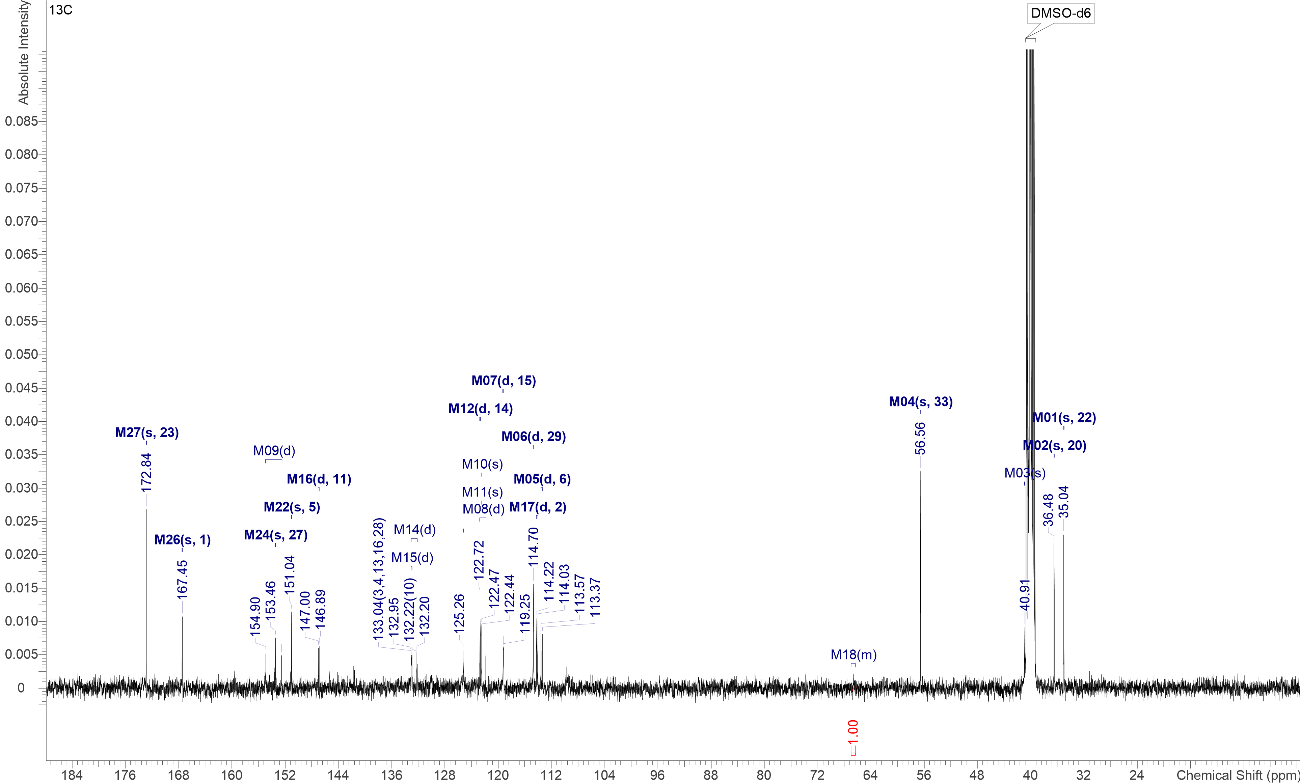


**Figure S41.** ^13^C-NMR spectra of compound **58**.

[M+H]^+^

*

**Figure S42.** Mass spectrum and UV spectrum from LC-MS measurement of compound **58**.

# **N-(3-amino-3-oxopropyl)-2-((4'-chloro-3-fluoro-[1,1'-biphenyl]-4-yl)amino)-3,4-difluorobenzamide (Compound 63)**


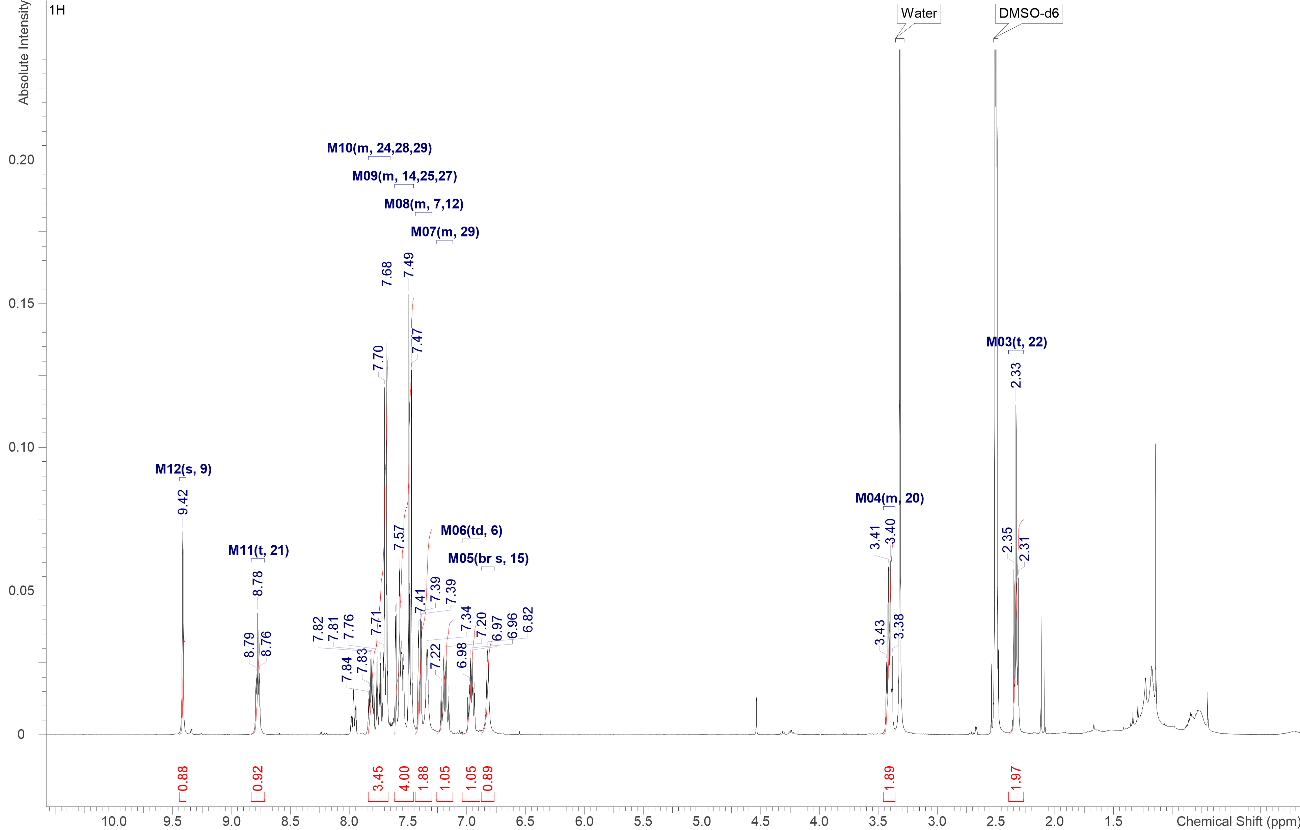


**Figure S43.** ^1^H-NMR spectra of compound **63**.


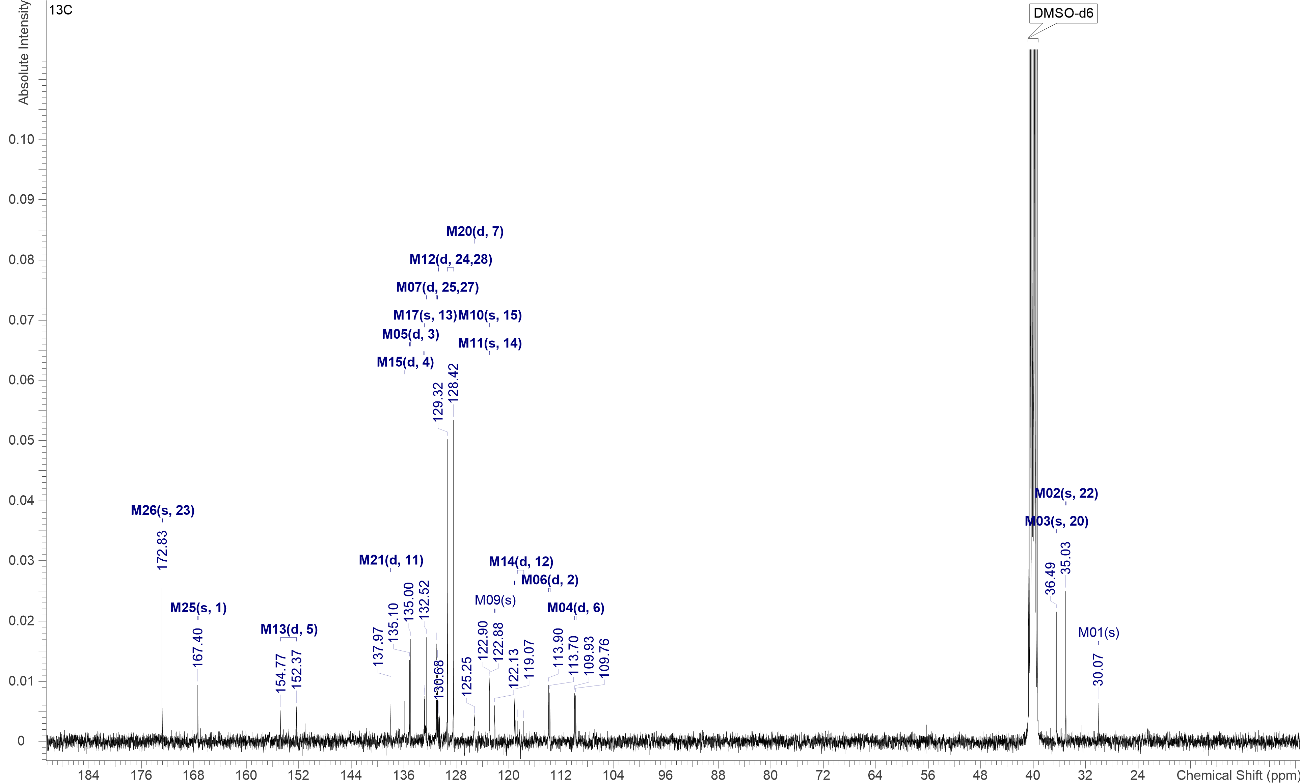


**Figure S44.** ^13^C-NMR spectra of compound **63**.

[M+H]^+^

*

**Figure S45.** Mass spectrum and UV spectrum from LC-MS measurement of compound **63**.

- 1. **N-(3-amino-3-oxopropyl)-2-((2'-butoxy-3-fluoro-5'-methyl-[1,1'-biphenyl]-4-yl)amino)-3,4-difluorobenzamide (Compound 69)**


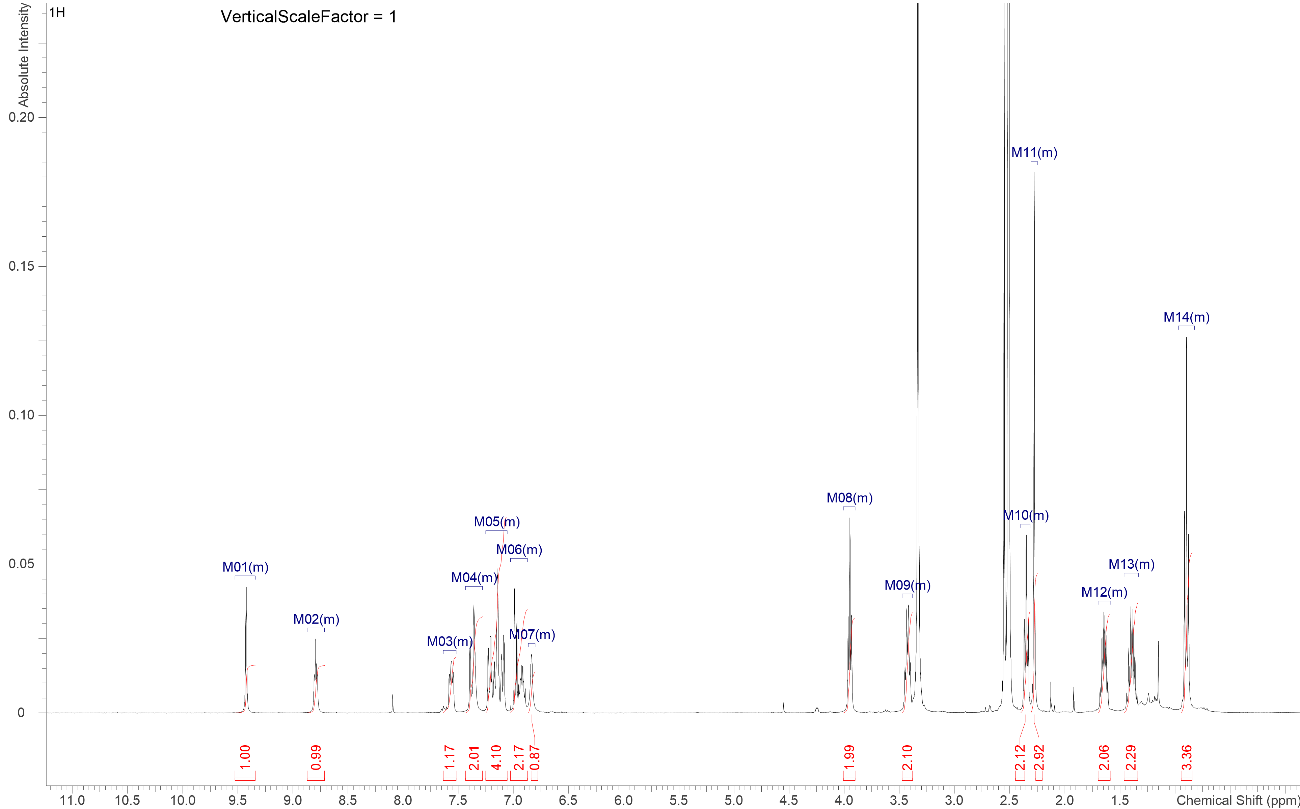


**Figure S46.** ^1^H-NMR spectra of compound **69**.


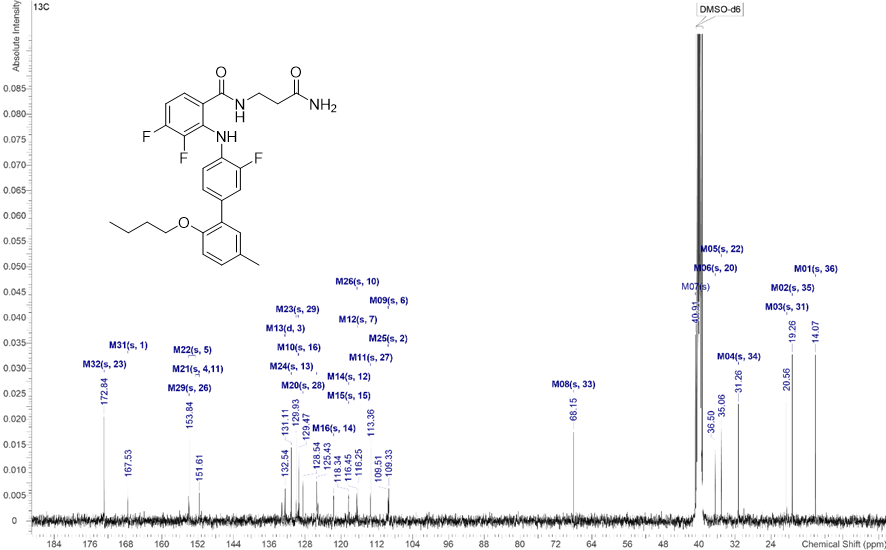


**Figure S47.** ^13^C-NMR spectra of compound **69**.

[M+H]^+^

*

**Figure S48.** Mass spectrum and UV spectrum from LC-MS measurement of compound **69**.

- 1. **N-(3-amino-3-oxopropyl)-3,4-difluoro-2-((3-fluoro-4'-hydroxy-[1,1'-biphenyl]-4-yl)amino)benzamide (Compound 73)**


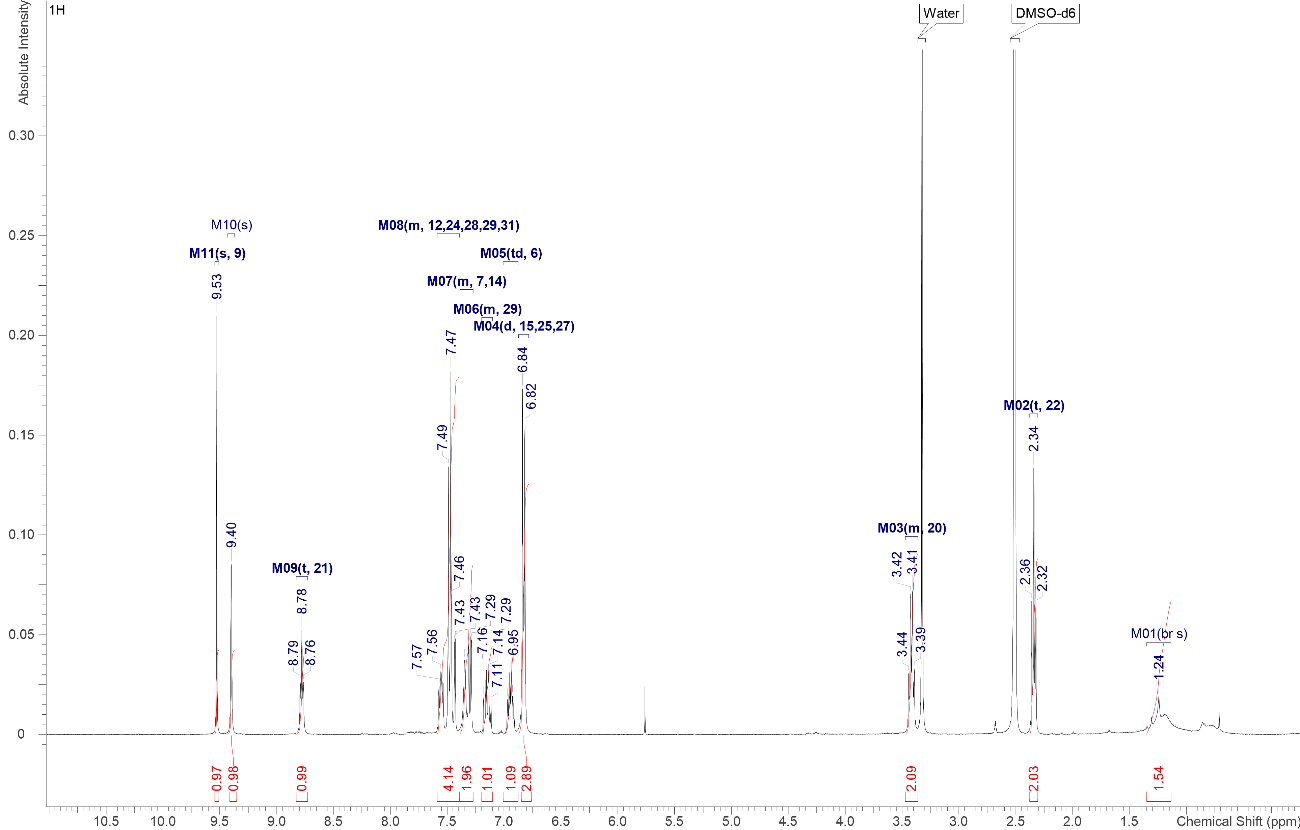


**Figure S49.** ^1^H-NMR spectra of compound **73**.


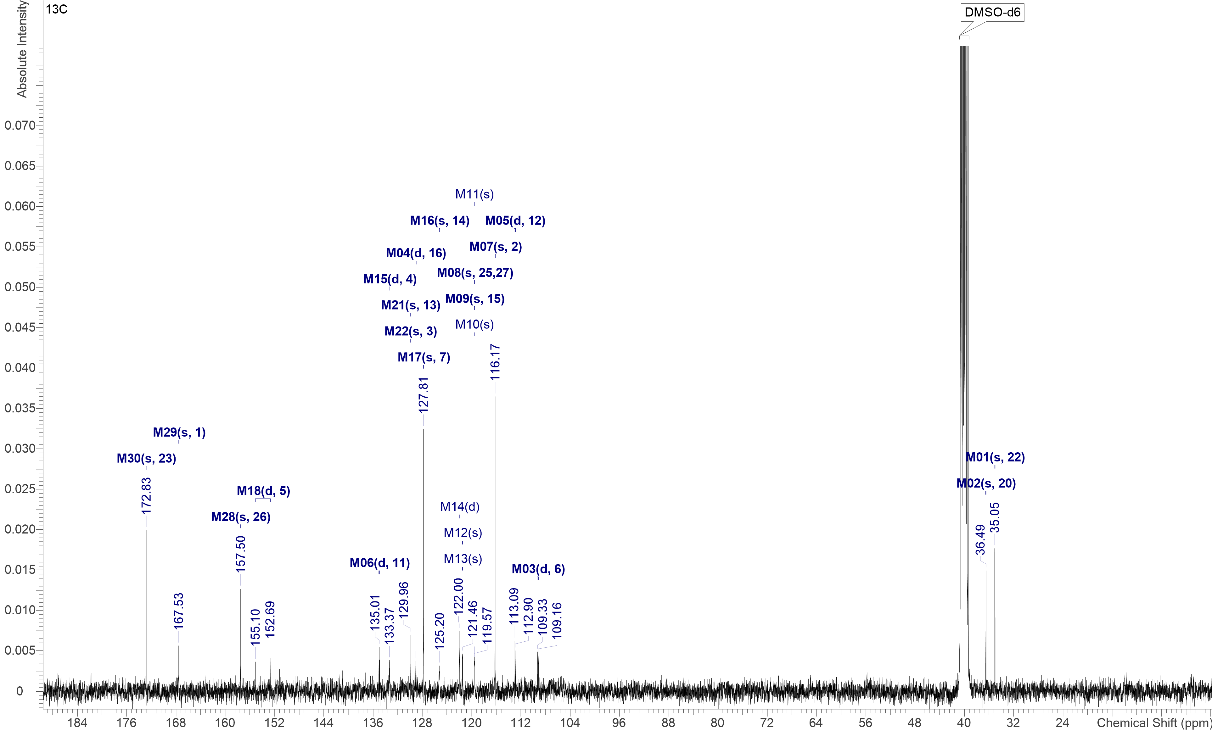


**Figure S50.** ^13^C-NMR spectra of compound **73**.

[M+H]^+^

*

**Figure S51.** Mass spectrum and UV spectrum from LC-MS measurement of compound **73**.
